# Synthesis of Population Trends Reveals Seascape‐Wide Reorganisation of Biodiversity From Microalgae to Birds

**DOI:** 10.1111/gcb.70298

**Published:** 2025-06-18

**Authors:** Anika Happe, Kasper J. Meijer, Jan‐Claas Dajka, Oscar Franken, Holger Haslob, Laura L. Govers, Michael Kleyer, Annebelle C. M. Kok, Lucie Kuczynski, Kertu Lõhmus, Sancia E. T. van der Meij, Han Olff, Lena Rönn, Alexey Ryabov, Anne F. Sell, David W. Thieltges, Britas Klemens Eriksson, Helmut Hillebrand

**Affiliations:** ^1^ Institute for Chemistry and Biology of the Marine Environment (ICBM), School of Mathematics and Science Carl von Ossietzky Universität Oldenburg Oldenburg Germany; ^2^ Groningen Institute for Evolutionary Life‐Sciences University of Groningen Groningen AG the Netherlands; ^3^ Helmholtz‐Institute for Functional Marine Biodiversity at the University of Oldenburg [HIFMB] Oldenburg Germany; ^4^ Alfred Wegener Institute, Helmholtz‐Centre for Polar and Marine Research [AWI] Bremerhaven Germany; ^5^ Department of Coastal Systems NIOZ Royal Netherlands Institute for Sea Research Den Burg the Netherlands; ^6^ Thünen Institute of Sea Fisheries Bremerhaven Germany; ^7^ Institute of Biology and Environmental Sciences, School of Mathematics and Science Carl von Ossietzky Universität Oldenburg Oldenburg Germany; ^8^ UMR ENTROPIE, Lucie Kuczynski IRD, IFREMER, CNRS University of La Reunion, University of New Caledonia Noumea New Caledonia; ^9^ Naturalis Biodiversity Center Leiden CR the Netherlands; ^10^ Lower Saxony Water Management, Coastal and Nature Protection Agency (NLWKN, Brake‐Oldenburg) Oldenburg Germany

**Keywords:** biodiversity assessment, conservation, ecosystem functioning, monitoring, North Sea, temporal population trends, Wadden Sea, winners and losers

## Abstract

Many monitoring programs aim to understand regional biodiversity patterns in relation to global and regional conservation targets, using either community‐wide biodiversity metrics to describe the community status or trends of pre‐selected “key” species as biodiversity change indicators. However, the former often lacks information on which species are changing, and the latter is heavily skewed towards specific taxa, potentially overlooking changes in other, functionally important taxa. We gathered an extensive set of monitoring data with over 3000 population trends (ranging from 5 to 91 years in duration) for a wide range of taxa across the Wadden Sea. We combined a systematic and quantitative categorization of population trends (weighted vote count) with a meta‐analysis on different taxonomic levels. This allowed the first cross‐taxa synopsis of species declines and increases and determined their directionalities throughout time. Our meta‐analysis showed an overall decrease in population size for fish, zooplankton, and plant species, while birds showed an overall increase. However, these increases mask recent negative trends within specific bird groups since the late 1990s. In contrast, fish populations exhibited declines over the entire monitoring period. Species with declining populations (losers) were phylogenetically related, whereas species with increasing populations (winners) represented various organismal groups. Directionality and onsets of change in population trends were temporally synchronized throughout several groups, such as bivalves, fish, and birds, and may provide warning signals for future local extinctions in these taxa. Our analysis moves beyond typical indicator species by including the entire species inventory of the system. Basal trophic levels of aquatic ecosystems, such as zooplankton and phytoplankton, are often missing from policy assessments but are among the most important organism groups for ecosystem functioning. Here, we show that without additional monitoring effort, a systematic analysis of population trends adds to our understanding of trophic and compositional restructuring of ecosystems.

## Graphical abstract

We present a systematic and quantitative analysis of population trends to capture the seascape‐wide reorganisation of biodiversity from microalgae to birds. First, we categorised population trends and found over one third of populations in the Wadden Sea to change, that is, increase or decrease. A time‐specific analysis identified critical time points of changes in the trend direction (e.g., from neutral to negative) and found that the populations within over 50% of all classes follow similar temporal patterns. Finally, we identified winners and losers across multiple taxonomic levels to help guide targeted conservation efforts.

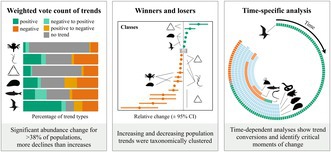

## 1 Introduction

The global biodiversity crisis is an increasing concern as shifts in species ranges and relative abundances not only restructure biodiversity but also impact ecosystem functioning and human well‐being (Pecl et al. 2017). However, as biodiversity comprises aspects of genetic, taxonomic, phylogenetic, and ecosystem diversity, unifying and generalizing this multifacetedness (Pereira et al. 2013) in biodiversity assessments is complex. Also, diversity can be captured on different scales as in alpha (within‐sample diversity), beta (between‐sample diversity), and gamma (regional) diversity. Moreover, biodiversity metrics do not always reflect ecologically meaningful processes, and some only provide a limited perspective of changes (Santini et al. 2017). For instance, the widely used species richness metric is sensitive to sampling effort and taxonomic resolution and does not align with rates of compositional turnover (Hillebrand et al. 2018). Simpson and Shannon diversity specifically capture both species richness and evenness (Hillebrand et al. 2018), but do not intuitively scale with species gain and loss (Roswell et al. 2021). To overcome these conceptual issues of traditional biodiversity metrics, the Hill number series (Hill 1973) provides a simple but more robust and logically reasonable approach to biodiversity by focusing more on dominant species and taking abundances into account (Chase and Knight 2013; Roswell et al. 2021; Antonucci Di Cavalho et al. 2023).

Analysing temporal trends with alpha diversity measures (e.g., species richness or the effective number of species) has provided insights into biodiversity dynamics (Dornelas et al. 2014; Rishworth et al. 2020), but this approach has limitations for inferring ecological mechanisms or informing policy. Ecologically, changes in local (i.e., alpha) biodiversity reflect only net changes in species number and not identity (Hillebrand et al. 2018). These changes, or the lack thereof, may not accurately reflect the actual changes in ecosystem properties and processes if, for instance, the declining species are replaced by functionally similar or different colonisers (Hillebrand et al. 2018; Eriksson and Hillebrand 2019). Also, analyses of changes in richness are strongly affected by the concept of extinction debt, when extinction is delayed after habitat deterioration (Tilman et al. 1994). Imbalances in temporal occurrences of colonisations and extinctions can bias biodiversity trends for decades (Jackson and Sax 2010; Kuczynski et al. 2023). More fundamentally, local species extirpation is the final step of decline, which is ideally detected much earlier.

An alternative approach is summarising population trends across species, which estimates population declines and increases. For example, the Living Planet Index (LPI) assesses trends in the relative abundance of vertebrate populations. However, the LPI has been criticised for not being an accurate, fully representative biodiversity indicator for planetary health; besides the fact that it only assesses vertebrates, it has been questioned as being sensitive to a multitude of mathematical assumptions (Jaspers 2020). For example, extreme individual trends can disproportionately influence aggregated indices (Finn et al. 2023). Moreover, the LPI relies on a geometric mean of trends across species (Loh et al. 2005) and does not specifically aim to identify increasing populations (winners) alongside decreasing populations (losers) (Finn et al. 2023). Here, we propose a synthesis of population trends within and across taxonomic groups to fully capture the ecosystem‐wide reorganisation of biodiversity.

**FIGURE 1:**
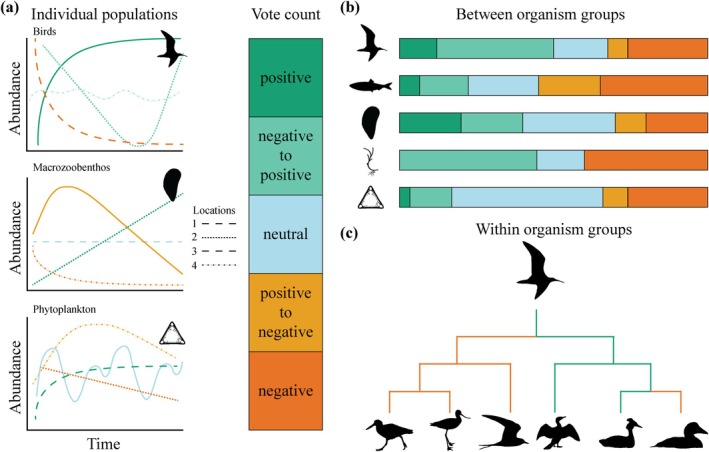
Conceptual figure of (a) individual populations temporally fluctuating in population response. The different panels represent theoretical examples of abundance trends of spatially separated populations per organism group. Different types of trends can be delineated here. Population trends can differ (b) between and (c) within organism groups. This figure does not represent actual data or results.

In long‐term monitoring data sets, species populations may fluctuate over time due to natural or anthropogenic perturbation events (Figure 1). Such fluctuations in spatially separated locations may cause populations of the same species to respond differently across regions and ecological settings (Figure 1a). However, spatial differences are often not incorporated within population assessments, although understanding the timing of changes on larger scales may help to understand the drivers behind population trends. Closely connected populations might show similar trends (Folmer et al. 2014), while opposite trends in spatially separated populations might cancel each other out, resulting in an overall neutral trend. Our approach uses a comparison of statistical models to assign each time series (population trend of each species at individual measurement stations) to one of five defined trend types (Figure 1b). Targeted meta‐analyses enable the calculation of overall trends across and within taxonomic groups (Figure 1c), while time‐specific trend analyses help identify critical points of change.

We use the Wadden Sea as a case study to highlight the benefits of a systematic and quantitative analysis of population trends to assess ecosystem reorganisation. Spanning the North Sea coastal regions of the Netherlands, Germany, and Denmark, the Wadden Sea is the world's largest connected sedimentary intertidal system (Kloepper et al. 2022). Its diverse habitats and high productivity support over 100 wetland bird species, over 150 fish species, three marine mammal species, and numerous benthic invertebrates (Kloepper et al. 2022). The Wadden Sea was declared a UNESCO World Heritage Site in 2009 and hosts Natura 2000 sites designated under the European Birds and Habitats Directives (Directive 2009/147/EC; Directive 92/43/EEC) as well as a range of National Parks. The area is managed as separate units, corresponding to different countries and protection zones, whereas, in reality, it is one connected seascape. Therefore, assessing the conservation status of the Wadden Sea benefits from a holistic area‐wide assessment of population trends. Ecological change is monitored in the Trilateral Monitoring and Assessment Programme (TMAP) as well as in national and international monitoring programmes. To date, the analysis of these data has focused on trends in traditional biodiversity metrics (e.g., richness) or selected populations (CWSS 2008; Kloepper et al. 2022), but a synthesis of these trends remains lacking.

We present generalised population trends across a wide range of organism groups and trophic levels throughout the Wadden Sea. We describe the temporal reorganisation of biodiversity in the Wadden Sea and identify clear winners and losers among taxonomic groups. Finally, we discuss how the added benefits of the approach provide a more holistic overview of the population dynamics in the Wadden Sea and thus facilitate targeted conservation practices.

## 2 Materials and Methods

### 2.1 Data Collection

**FIGURE 2:**
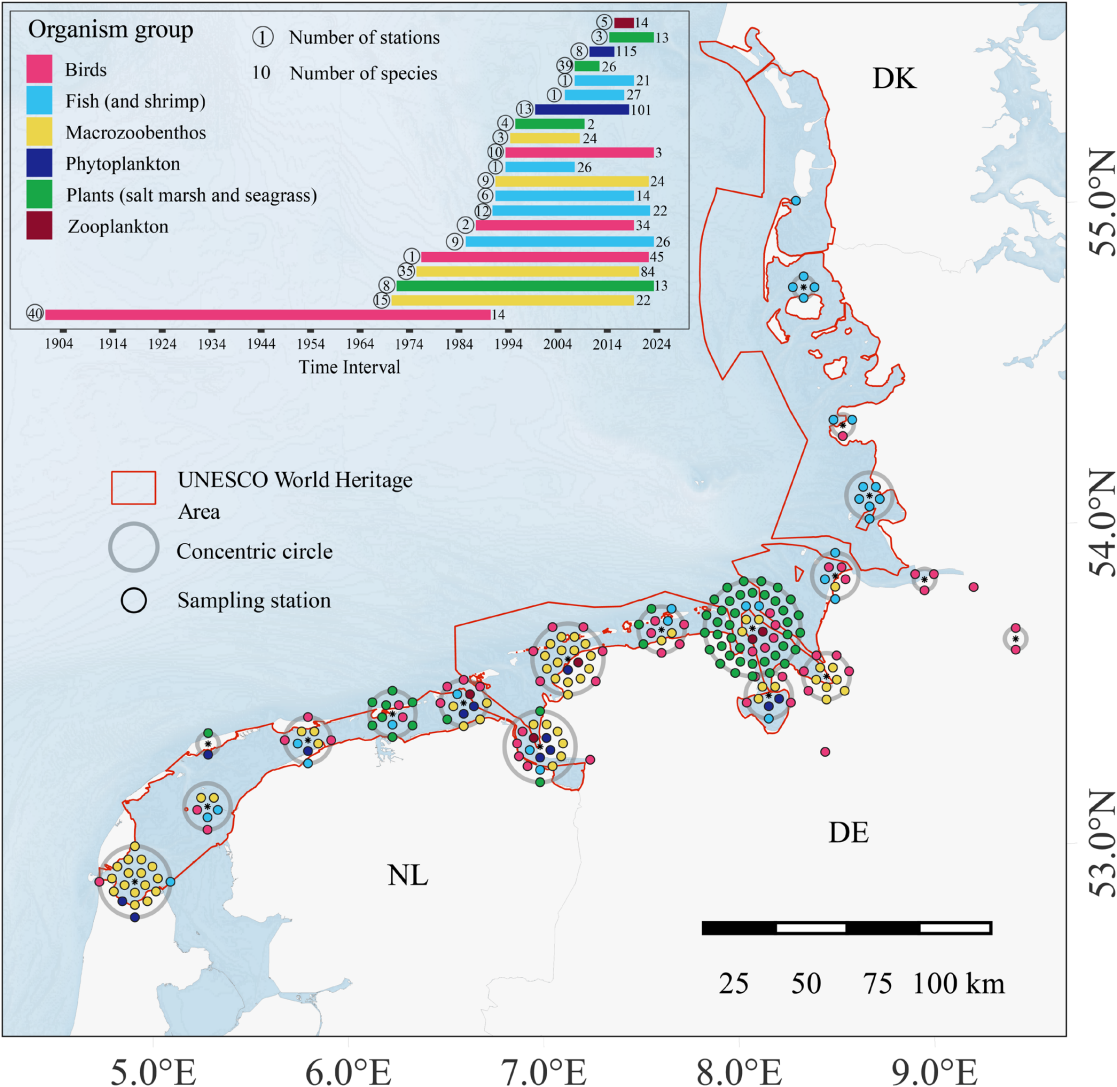
Overview of monitoring stations and periods for each organism group. Points reflect individual stations for the respective group (colour) within the radius of the concentric circle. The red outline indicates the official UNESCO World Heritage boundaries. Map lines delineate study areas and do not necessarily depict accepted national boundaries.

We combined 20 data sets from national monitoring programs and scientific studies, covering 401 species covering microalgae to birds throughout the Wadden Sea (Figure 2). Monitoring periods of the datasets ranged from 5 to 91 years (with a median of 30 years). Data on birds were collected on the Dutch parts from Netwerk Ecologische Monitoring (www.sovon.nl) and DeltaMilieu Projecten (Sluijter et al. 2023) and the German parts from Lower Saxony National Park monitoring efforts (Kleefstra et al. 2022). Fish data from the entire Wadden Sea were downloaded from the ICES database on trawl surveys (ICES, n.d.; www.ices.dk), supplemented with additional survey data from Thünen Institute of Sea Fisheries for the German parts (ICES DYFS and survey database: GASEEZ—German Autumn Survey in the Exclusive Economic Zone). Macrozoobenthos data were obtained from a long‐term sampling program of the Royal Netherlands Institute for Sea Research on the tidal flat Balgzand (Beukema and Dekker 2020) and national monitoring programs (MWTL) collected by Rijkswaterstaat in the Netherlands (Van der Jagt et al. 2023) and in Germany (Rishworth et al. 2020; Dajka et al. 2022). Phytoplankton data were derived from Dutch and German monitoring programs as described in Antonucci Di Cavalho et al. (2023). The phytoplankton data also include two taxa of cyanobacteria. Zooplankton data originated from the Lower Saxony Water Management, Coastal and Nature Protection Agency (NLWKN). Finally, plant data were obtained from long‐term saltmarsh studies on the islands of Schiermonnikoog (Olff et al. 1997), Spiekeroog (Balke et al. 2017; Lõhmus et al. 2020) and Mellum (Kleyer et al. 2014) and a national seagrass monitoring program in the Dutch Wadden Sea (Folmer 2015). For a more detailed description of the study site, see Supporting Information and Kloepper et al. (2017).

We standardised all taxon names across datasets, checked synonyms, and updated their taxonomic classification based on WoRMS Editorial Board (2024) and AlgaeBase (Guiry and Guiry 2024). When needed, primary taxonomic literature was consulted to reflect the most recent classification of the species (Table S1).

### 2.2 Statistical Analysis

#### 2.2.1 Fitting Population Trends

We conducted all statistical analyses in R version 4.3.1 (R Core Team 2023) and all data and code are provided in Happe and Meijer et al. (2025). We first aggregated all monitoring data to annual means per location. Only species recorded for at least 5 years and present in at least 50% of the monitoring period were included in subsequent analyses to avoid biases by infrequent observations of extreme population differences. We then fitted population trends separately for each species at each location using linear and second‐order polynomial regressions with year as the predictor and abundance measure as the response variable. Since data distributions can differ, Gaussian, Poisson, and negative binomial models were applied to all species. Abundance data was ln(x + 1) transformed for the Gaussian models, and values were rounded off to the nearest integer for Poisson and negative binomial models. We tested Gaussian and Poisson models for normality and homoscedasticity of the residuals. Poisson models were also checked for overdispersion. In the case of overdispersion, negative binomial models were used. The Akaike information criterion (AIC) was used to select the most parsimonious model in case multiple models indicated significant trends and met model assumptions. *p*‐values and AIC were used to choose between the best‐fitting trends (linear or second‐order polynomial). Nine trend types were identified based on the coefficients of the best model (Figure S1). A unimodal trend (as negative to positive or positive to negative) was assigned if the polynomial model was the most parsimonious, coefficients had opposite signs (negative and positive) and the mode fell within the observation period, verified by the MOStest (Mitchell‐Olds and Shaw 1987). Accelerating or decelerating, positive or negative trends were assigned in case the polynomial model did not meet these requirements but was the most parsimonious (Figure S1). Linear negative or positive trends were concluded in case the linear model was the most parsimonious. Neutral trends were assigned if no significant annual trend was detected despite conforming to model assumptions (Figure S1).

To evaluate the probability of detecting non‐neutral trends, we performed a binomial analysis using monitoring duration as a predictor variable. To account for pseudo‐replication, sampling station and species were included as separate random factors. Only neutral and non‐neutral trends that met model assumptions were included. Additionally, we performed a binomial analysis to assess the probability of finding a model that meets assumptions using the same predictor and random factors to determine whether linear model assumptions hold for longer time series.

#### 2.2.2 Weighted Vote Count

To assess the relative distribution of trend directions, we grouped positive directional trends (positive accelerating, positive decelerating and positive linear) and negative directional trends (negative accelerating, negative decelerating and negative linear) into ‘positive’ and ‘negative’ trend types, respectively. We then weighed the relative distribution of the types of trends by the number of years with observations (Wirth et al. 2024) across all population trends or separated by ecosystem components. Species representation in the meta‐analyses reflects their representation in the dataset, that is, species monitored at more locations have higher representation. The total number of monitoring locations for each species is provided in Table S1.

#### 2.2.3 Meta‐Analyses on Multiple Organisational Levels

To test for significant overall trends within and across ecosystem components, we conducted subsequent meta‐analyses in which we only included the population trends that fitted a linear regression without violating assumptions (2298 out of 3058 trends, 75%), regardless of whether polynomial predictors, Poisson or negative binomial error distributions, lowered AIC. For the meta‐analyses, we used multi‐level random‐effects models using the ‘*rma.mv*’ function from the ‘metafor’ package (Viechtbauer 2010). The slope of the linear regression was used as the effect size, and the squared standard errors as the corresponding sampling variance, giving more weight to reliable trends, typically from longer time series. Station identity was included as a random effect, and the model was fitted using the restricted maximum likelihood (REML) estimation method for unbiased variance component estimates under random effects. To extract group‐specific trends, the ecosystem component was added as a moderator variable in a following meta‐analysis. To determine overall directionality across all ecosystem components (i.e., slopes differing from zero) and account for the cancellation of positive and negative trends, we repeated the ecosystem‐wide meta‐analysis using the absolute values of the slopes.

To identify taxonomic groups as winners and losers, we repeated the meta‐analysis using the slopes as the effect size (as described above) but added the taxonomic rank class as a moderator variable in a model without intercept. This provided class‐wide effect sizes with 95% confidence intervals (CIs); if a CI did not overlap with 0, the entire class either increased or declined in population sizes. Class rank was chosen to get a deep enough insight into structural changes while ensuring sufficient entries per group (Table S3). We also performed the same analyses on group‐specific subsets of the dataset on genus, family, and order level to get an insight into specific genera, families, or orders driving class‐level trends. A dendrogram was created using the *‘ggtree’* function from the ‘ggtree’ package (Yu 2022) to visualize the winners and losers across taxonomic ranks. The branches were colored based on the meta‐analysis results, with positive or negative trends assigned if the CI did not overlap 0.

#### 2.2.4 Time‐Specific Analysis

To analyse the timing of trend directionality, we used coefficients from the most parsimonious models to calculate the annual rate of change in each population trend using the derivative as a proxy for the slope of the population trend in each year. With this rate of change (negative, positive or equal to zero), we assigned a yearly trend direction (negative, positive, or neutral) for each population during the monitoring period. Neutral trends were assigned if the rate equalled zero or if no trend was detected in the first step. Thereby, each population has three possible states each year. We applied multinomial random logit models using the ‘*mblogit’* function from the ‘mclogit’ package (Elff 2022) to estimate the probability of each state for different taxonomic groups over time. These models estimate the probability of one state (here, positive or negative trends, respectively) against a reference state (here, a neutral trend). A binomial distribution was used in case only two types of trends were found for a taxonomic group, where then the probability of one state was modelled against the probability of the second state. Species and locations were included as random effects to account for repeated measures. Only models where the variable ‘year’ significantly predicted the trend state were considered. AIC was used to determine whether a second‐order polynomial should be included in the model. We calculated 95% CIs by bootstrapping the estimated values. Estimated probabilities and CIs for positive and negative states were compared to deduce predominant trend directions. The state with the highest probability was considered the predominant state unless both were below 50%, in which case a neutral trend was assigned. If the probability and the CIs overlapped, the trend was considered cancelled out by equal positive and negative trends (see Figure S2 for an example).

## 3 Results

### 3.1 Trends and Weighted Vote Count

We analysed a total of 3058 population trends, of which 1862 showed no clear directionality, 355 were positive, 104 shifted from negative to positive, 167 shifted from positive to negative, and 570 were negative. Thus, population trends across all organism groups show a much higher percentage of significant trends (38.2% i.e., the share of directional trends in the total number of trends) than predicted by chance (*p*‐value and type I error alpha = 5%), with more negative than positive trends at the end of the monitoring period (737 and 459, respectively).

**FIGURE 3:**
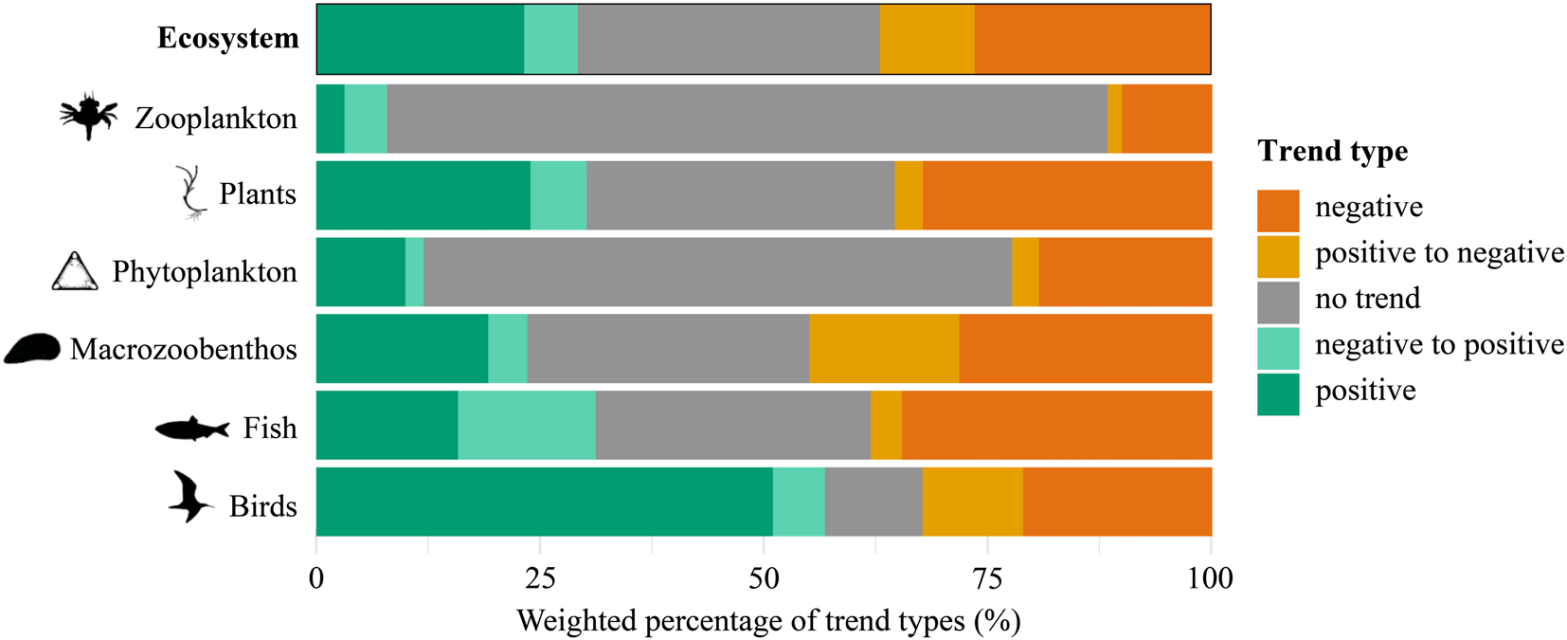
The weighted vote count as the percentage of each trend type in all ecosystem components together (top row) and separately (for the groups specified on the left axis) is weighted by the number of years with observations. Numbers of trends and species per organism group: Zooplankton (56 trends, 14 species), plants (205 trends, 33 species), phytoplankton (1111 trends, 161 species), macrozoobenthos (938, 96 species), fish (382 trends, 40 species), and birds (350 trends, 57 species). The organism group of “Plants” includes salt marsh plants and seagrasses. Table S2 provides an overview of the number of entries in each taxonomic level and organism group.

Weighting the trend types by the number of observation years increased the proportion of significant trends to 66.2% across organism groups (Figure 3). The proportion of positive and negative trends was roughly similar (Figure 3), and consequently, the overall slope of the quantitative meta‐analysis was close to zero (Table 1). When using absolute slopes (i.e., the direction of signs removed), the overall slope showed a highly significant deviation from 0 (Table 1), reflecting the large weighted proportion of significant trends. When separating the meta‐analysis for organism groups, marine birds showed significantly positive trends in population size overall. In contrast, zooplankton, fish, and plants displayed a negative directionality of population trends. No consistent trend was found for phytoplankton and macrozoobenthos.

**TABLE 1:** Top: Meta‐analysis output testing the slope (All) or absolute slope (All (absolute)) of the linear regression from all populations as the effect size with squared standard errors as the corresponding sampling variance and the station identifier as a random effect; Bottom: Meta‐analysis output testing the signed slope of the linear regression moderated by the ecosystem components at the class level.

	Estimate	SE	Zval	CI, lower	CI, upper	*p*
All (absolute)	0.0632	0.0031	20.5501	0.0571	0.0692	< 0.0001*
All	−0.0052	0.0044	−1.1979	−0.0137	0.0033	0.2309
Phytoplankton	−0.0071	0.0104	−0.6820	−0.0275	0.0133	0.4953
Zooplankton	−0.0468	0.0200	−2.3378	−0.0861	−0.0076	0.0194*
Macrozoobenthos	0.0043	0.0046	0.9409	−0.0047	0.0133	0.3468
Fish	−0.0150	0.0049	−3.0428	−0.0247	−0.0053	0.0023*
Birds	0.0243	0.0060	4.0437	0.0125	0.0360	< 0.0001*
Plants	−0.0468	0.0200	−2.3378	−0.0563	−0.0356	< 0.0001*

*Note:* The category “plants” includes salt marsh plants and seagrasses.

* Significance level of < 0.05.

The probability of finding a significant trend for a population increased with the monitoring duration (Binomial regression, B = 0.015, 95% CI = [0.007, 0.022], *χ* ^*2*^ = 15.47, *p* < 0.001; Figure S3a). However, the probability of detecting a trend using linear regression that fits model assumptions under Gaussian, Poisson, or negative binomial error distributions, including the possibility of a second‐order polynomial, decreases with monitoring duration (Binomial regression, B = −0.048, 95% CI = [−0.060, −0.036], *χ* ^*2*^ = 65.13, *p* < 0.001; Figure S3b).

### 3.2 Meta‐Analysis: Winners vs. Losers

**FIGURE 4:**
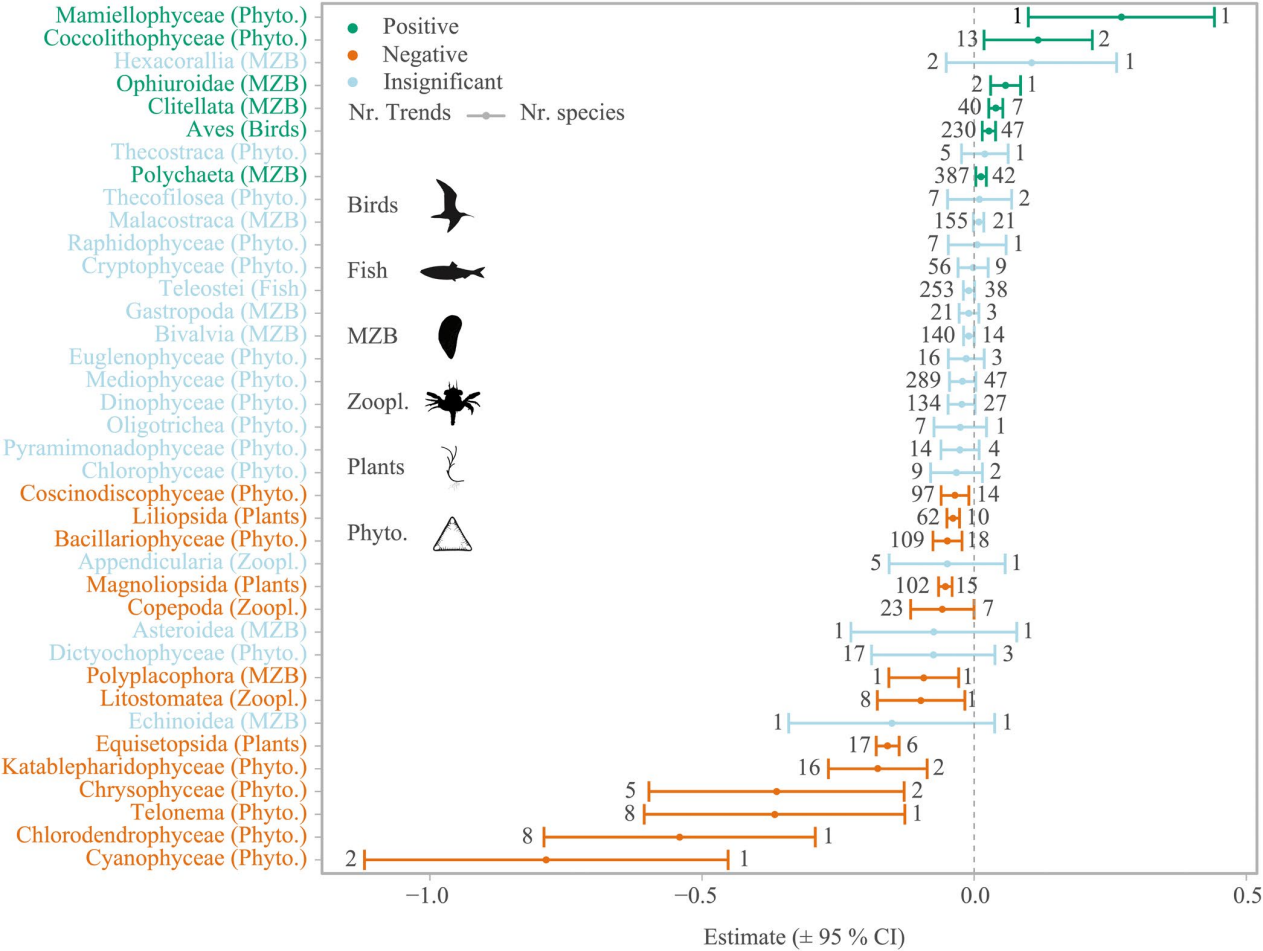
Estimates and 95% confidence intervals (CI) derived from the meta‐analysis to identify winners (green) and losers (orange) for classes. Blue indicates no significant difference from 0. The abbreviations refer to phytoplankton (*Phyto*.), macrozoobenthos (MZB), and zooplankton (Zoopl.). The number left of the lower CI indicates the number of trends in the analysis, and the number to the right of the upper CI indicates the number of species. Significance (*p* < 0.05) is indicated by CI not crossing 0. Detailed figures for birds (Figure S5), zooplankton (Figure S6) and macrozoobenthos (Figure S7) on family level can be found in the supplements.

**FIGURE 5:**
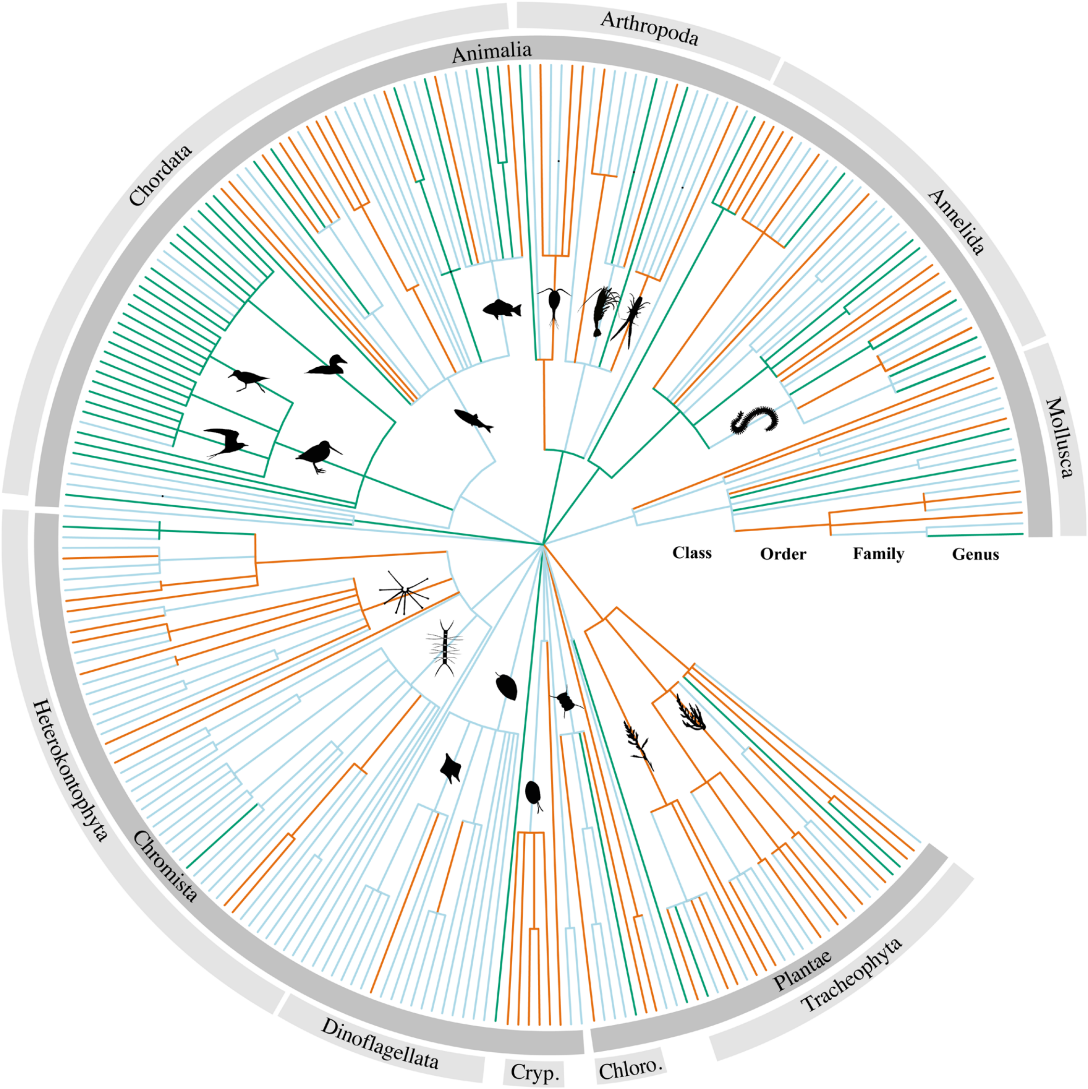
Dendrogram of the meta‐analysis results (coloured branches). The colour indicates an overall significantly positive trend (green), negative trend (orange) or a non‐significant overall trend (blue). The dark grey circle indicates the kingdom, and the light grey circle indicates the phylum. Abbreviations are as follows: Cryptophyta (Cryp.) and Chlorophyta (Chloro.). The dendrogram does not represent phylogenetic distances. Figure S3 shows the dendrogram with genus labels. The estimates and 95% confidence intervals for each taxonomic level are presented in Table S3.

Identifying winners and loser class ranks reveals a high abundance of primary producers on the loser side (estimate and CI_max_ < 0), with 10 out of 13 significantly negative classes belonging to either plants or phytoplankton, whereas phytoplankton represents the five classes with the most negative trends (Figure 4). Negative trends appear to group taxonomically for all classes within the phylum Tracheophyta (vascular plants) and all families within the class Cryptophyceae (unicellular flagellates) (Figure 5). In the kingdom Animalia, negative trends are found in several genera (11/32) of the class Teleostei (fish). The class Aves (birds), however, displays positive trends for most genera (24/28) (Figure 5). The phyla Annelida (segmented worms), Arthropoda, and Mollusca present a more balanced pattern with winners and losers within the same classes and orders (Figure 5). The macrozoobenthos class Polyplacophora (chitons) and the two zooplankton classes Litostomatea (here consisting only of the ciliate *Mesodinium rubrum* ) and Copepoda belong to the losers, whereas the three macrozoobenthos classes Ophiuroidae (brittle stars), Clitella, and Polychaeta (both annelid worms) were identified as winners. On the family level, we find more nuance in which families perform well or poorly (see Supporting Information).

### 3.3 Temporal Trend Analysis

**FIGURE 6:**
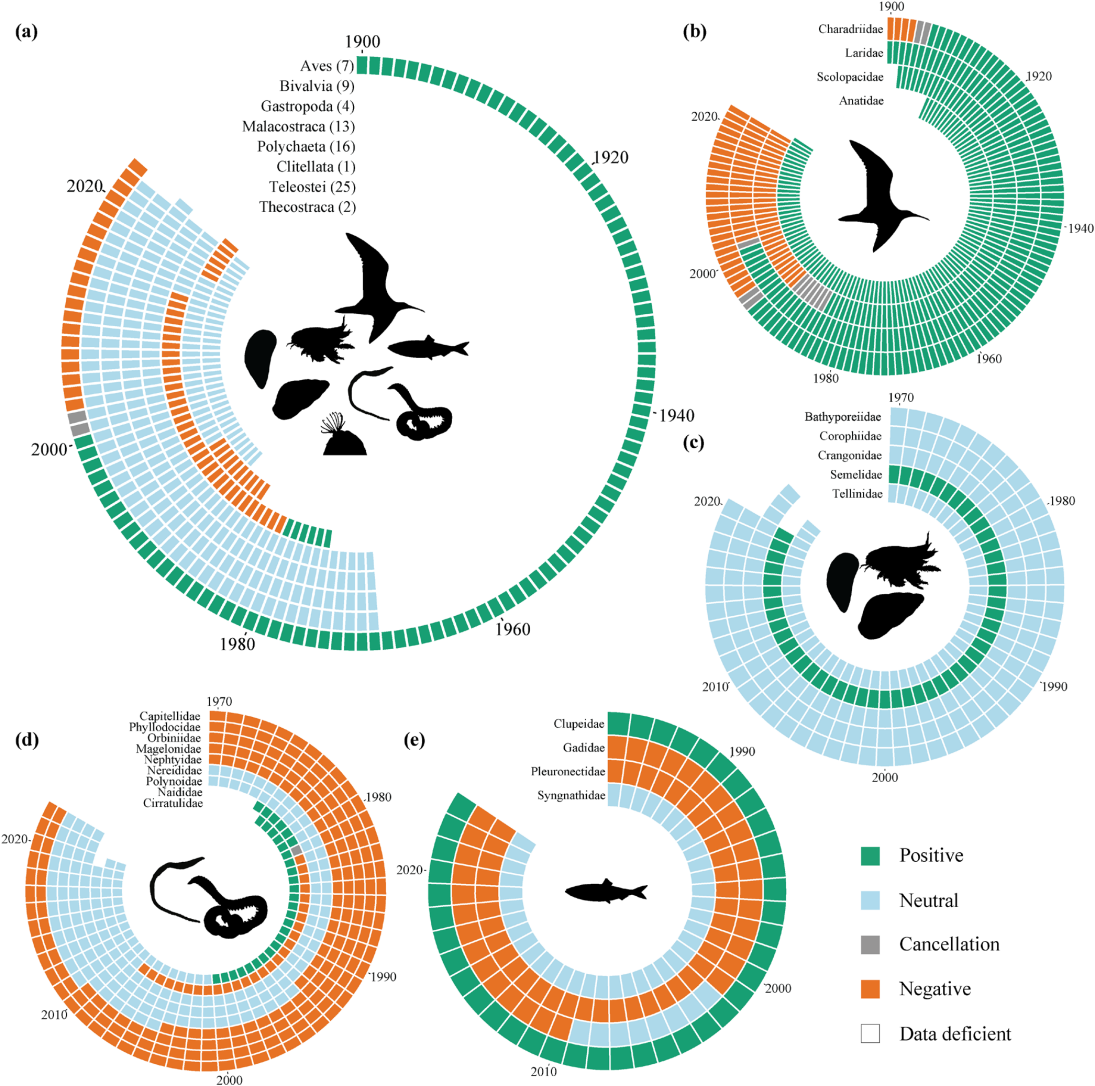
Significant temporal population trends for animals. Trend timeline on (a) class‐level aggregation (number of families within the class in brackets), (b) families within the Aves class, (c) families within the classes Bivalvia, Gastropoda and Malacostraca, (d) families within the classes Clitellata and Polychaeta, (e) families within the Teleostei class. Trend timelines are only shown for taxa where “year” was a significant predictor for the trend in multinomial models. No families within the Thecostraca class had significant models (Table S3). A cancellation is assigned if equal positive and negative trends cancel each other out.

**FIGURE 7:**
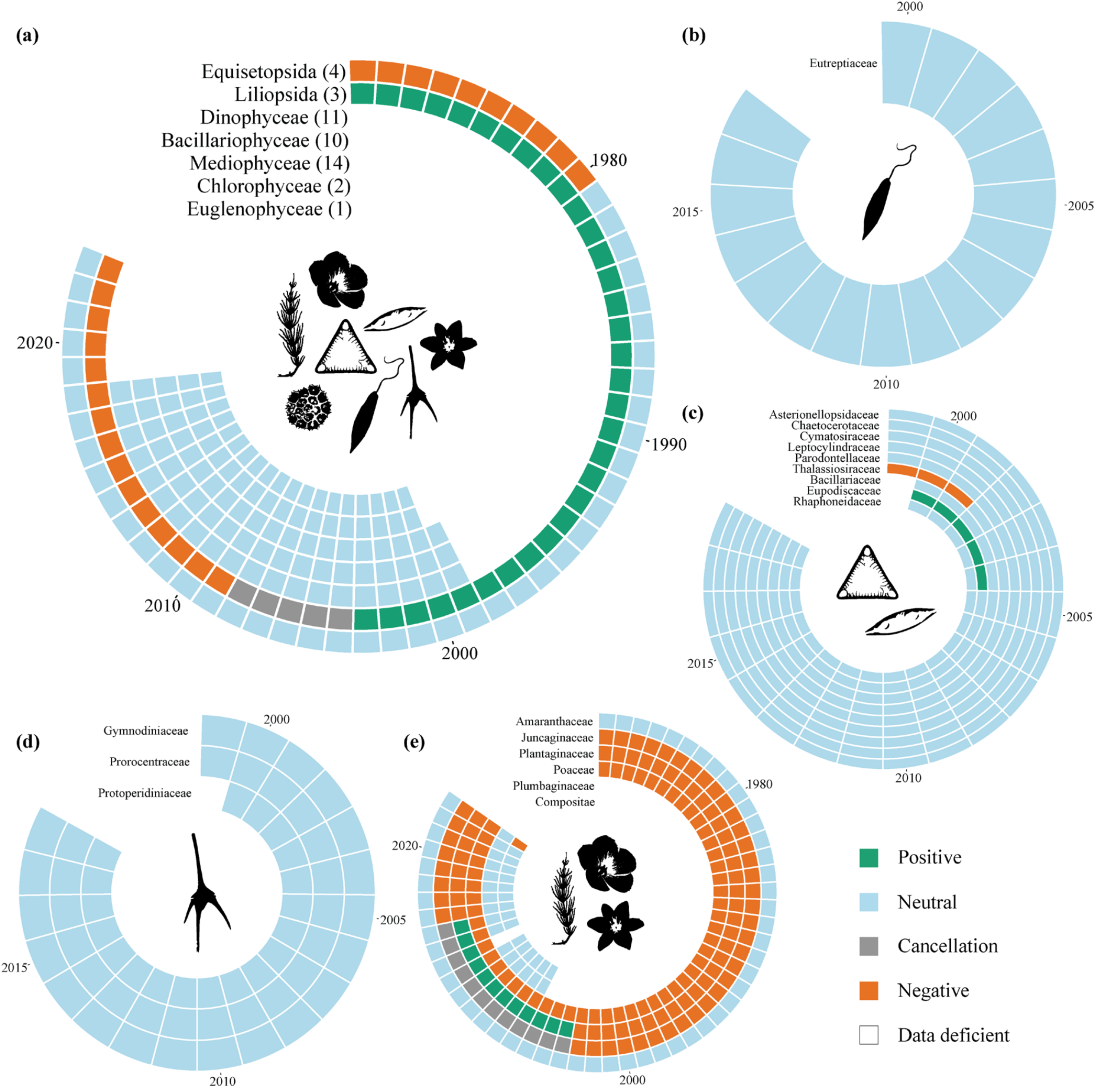
Significant temporal population trends for plants and phytoplankton. Trend timelines on (a) class‐level aggregation (number of families within the class in brackets), (b) families within the Euglenophyceae class, (c) families within the Bacillariophyceae and Mediophyceae classes, (d) families within the Dinophyceae class, (e) families within the classes Equisetopsida, Liliopsida and Magnoliopsida. Trend timelines are only shown for taxa where “year” was a significant predictor for the trend in multinomial models. No families within the Chlorophyceae classes had significant models (Table S3). A cancellation is assigned if equal positive and negative trends cancel each other out.

Trends and directional changes of trends for 16 out of 32 analysed classes were temporally synchronised over all populations within (Figures 6 and 7; Table S3). The birds (Aves) shift from initially positive to negative trends with a turning point in trend direction around the mid‐1990s to early 2000s. Within the Aves, trends for four of seven families were temporally synchronised (Figure 6b; Table S3). Most families show similar patterns as on class level, specifically for the families Scolopacidae (e.g., sandpipers and snipes), Charadriidae (e.g., plovers and lapwings), and Laridae (including gulls and terns) (Figure 6b). In contrast, the Anatidae (ducks, geese, and swans) remain positive overall (Figure 6b). The Thecostraca (barnacles, two families), Malacostraca (malacostracan crustaceans, 16 families), Gastropoda (five families), and Bivalvia (nine families) show overall consistently neutral trends (Figure 6a). Within these classes, the Semelidae (clams) diverged from the class trend and instead showed mostly positive trends (Figure 6c). Temporal trends within the class Polychaeta were neutral overall (Figure 6a) but showed high variability between families. While most families shifted from negative to neutral trends, the Cirratulidae shifted from positive to neutral, whereas the Phyllodocidae (paddle worms) and Capitellidae remained consistently negative (Figure 6d). Lastly, fish (Teleostei) showed negative trends overall, with an intermediate period of neutral trends (Figure 6a). Temporal trends could be predicted significantly for four out of ten families (Figure 6e; Table S3). The Syngnathidae (represented by pipefishes, *Syngnathus* spp.) remain neutral, the Pleuronectidae (righteye flounders) showed negative trends, and the Clupeidae (herrings) remained positive within the monitored time series (Figure 6e). The Gadidae (codfish) showed largely negative trends except for a neutral period between 2002 and 2009 (Figure 6e).

Trends remained largely neutral within the Bacteria, Chromista, and Protozoa (Figure 7a), which also were consistently reflected within their families (Figure 7a–d). Only the Plantae showed variable trends over time. The Equisetopsida (horsetails, five families) showed neutral trends over time, whereas Liliopsida (including grasses, three families) shifted from a positive to a negative trend in the early 2000s (Figure 7a). Within these classes, Poaceae (grasses) were negative overall, similar to Plantaginaceae (plantains) but had an intermediate positive period, while Plumbaginaceae (leadworts) and Amaranthaceae remained neutral across time (Figure 7e). The Compositae also showed neutral trends until its shift into a negative trend in the last monitoring year (Figure 7e).

## 4 Discussion

Our systematic generalisation of 3058 populations across six ecosystem components revealed a continued reorganisation of marine biodiversity in the Wadden Sea. We find general declines for zooplankton, fish, and plants, whereas birds exhibited an overall positive trend in population size. Many of these overarching organism groups exhibit both positive and negative trends of smaller taxonomic units within the same group. Moreover, a temporal trend analysis revealed shifts in trend signs over time, a result often obscured by general trend analysis. For example, the generally positive trend for marine birds masks the drastic shift to a population decline between the late 1990s and early 2000s, giving early warning signals for local species extinctions. The clear directionality of changes in several ecosystem components and the temporal changes of trend directions allow for the identification of both winners and losers on multiple taxonomic resolutions to guide conservation efforts and better understand the reorganisation of biodiversity.

### 4.1 Population Trends and Biodiversity Changes

Our results generally align with the individual species trends reported in the quality status reports (QSR) for the Wadden Sea produced by the trilateral monitoring and assessment programme (TMAP) (Kloepper et al. 2022). However, a holistic biodiversity assessment for the Wadden Sea through the TMAP is complicated as species groups are assigned to different expert groups. This causes varying methodological approaches to determining population trends depending on the species group. For example, generalized additive models (GAMs) are grouped by tidal basins or regions to assess trends for macrozoobenthos and fish. In contrast, for breeding birds, the mean annual rate of population change and for migratory birds, a “flexible trend” obtained via TrendSpotter is used (Visser 2004; Kloepper et al. 2017, 2022). In addition, not all species groups are represented equally within these assessments. Following the water framework directive (WFD), marine phytoplankton is currently not assessed on a community level by TMAP, which only considers phytoplankton via chlorophyll‐a as a biomass indicator for eutrophication and water quality and blooms of the indicator species *Phaeocystis* sp. (Kloepper et al. 2022). Moreover, microphytobenthos, an important driver of the intertidal food web (Christianen et al. 2017), is currently not monitored (Wirth et al. 2024). This monitoring gap has also resulted in microphytobenthos not being included within our data sets. Still, our results indicate structural changes by a decrease in various phytoplankton classes, with potential functional implications at the base of the food web. A standardized approach categorizing population trends across taxonomic groups allows for more direct comparisons of biodiversity reorganization in all trophic levels.

The temporal trend analysis reveals shifts in trend directions between and within organism groups otherwise overshadowed by dominant trends. For example, the meta‐analysis classifies birds as winners, whereas the temporal trend analysis on the family level shows that this classification only holds until the early 2000s. The probabilistic direction of their trend switched from positive to negative in this period. The decline in Scolopacidae (e.g., sandpipers and snipes) since 1992 was followed by a decline in Charadriidae (e.g., oystercatchers, plovers, and lapwings) in 1996 and subsequently in Laridae (e.g., gulls, and terns) in 2003. Pressures that led to these changes could be linked to pressures in breeding or wintering areas for migratory species but may be exacerbated by changes within the Wadden Sea. Drastic declines in food resources, for example crashes in local intertidal mussel and cockle populations (Herlyn and Millat 2000; Imeson and Van den Bergh 2006) likely reinforced negative trends of shellfish‐eating birds in the 1990s (Beukema et al. 2015). For breeding birds, the number of declining species rises (Kloepper et al. 2022) likely resulting from poor breeding success (Van der Jeugd et al. 2014) due to increased predation risk (e.g., by mustelids, and racoons) and increasing intensity and frequency of heavy flooding (Van de Pol et al. 2010; Kloepper et al. 2022). Incorporating temporal analysis of changes within a time series allows for identifying critical time points of drastic reorganization.

Population trends heavily depend on the spatial scale at which populations are followed. On a global scale, trends tend to become muddled (Johnson et al. 2024). However, too small scales may give inaccurate perspectives of systemic changes. For example, Beukema and Dekker (2020) conclude that Wadden Sea‐wide decreases in the Baltic clam *Macoma balthica* and increases in the sand gaper *Mya arenaria* are likely caused by trophic changes and warming based on data from the tidal flat Balgzand. However, using Wadden Sea‐wide monitoring data, we find decreasing trends for both species. Still, even the entire Wadden Sea ecosystem might be too small‐scale to contextualize pressures driving population trends, especially for migratory fish and birds with additional pressures along their migration pathways or overwintering habitats (Shaw 2016). In this context, it is important to consider the scale at which a population is defined and monitored. For example, the local populations of migratory birds in our analyses represent a subset of a larger global population, while macrozoobenthos comprises smaller local meta‐populations forming a larger population within the connected ecosystem. The pressures acting upon these populations also vary, where highly mobile and migratory species are more susceptible to changes over larger spatial scales, and less mobile species are more subject to local pressures (Runge et al. 2014). These pressures can also change throughout ontogeny and drive the observed population trends.

The Wadden Sea is an important nursery area for many fish (Kloepper et al. 2022; van der Veer et al. 2022), but adult and juvenile populations undergo different pressures. Although the function of the Wadden Sea as a nursery for fish has stabilised over the last decade after declining since the 1980s (van der Veer et al. 2022; Kloepper et al. 2022), we still find declining trends among many fish families. For example, the flatfish families Pleuronectidae (righteye flounders) and Soleidae (true soles; represented by one species: *Solea solea* ) that use the Wadden Sea during juvenile life stages (van der Veer et al. 2022) were identified as clear losers, even though a species‐specific analysis in Kloepper et al. (2022) has shown that the decrease in juvenile flatfish species has recently levelled off. Whether this is driven by local pressures or by pressures off‐shore remains unclear (van der Veer et al. 2022). Identifying the pressures behind the observed population trends is beyond the scope of this research, but it is an obvious next step in order to also implement targeted management interventions.

Interestingly, we find that losers are phylogenetically related, whereas winners are more heterogeneously spread across organism groups. Shared life history strategies within organism groups might explain this phylogenetically related decline. Moreover, life history traits associated with both winners and losers might give insights into generalizations of characteristics that make certain species groups more vulnerable to population decline (Chichorro et al. 2019). For example, we find declines for larger‐bodied, predatory, and long‐lived fish species (e.g., *Gadus morhua* , *Trachurus trachurus* ), for which declines might be caused from outside the Wadden Sea but also larger‐bodied polychaetes and bivalves such as *Ampharete* sp. and *Mya* sp. Moreover, our meta‐analysis indicates that the reorganization of macrozoobenthos is largely driven by the strong increase of alien species, such as the Ostreidae (Pacific oysters, *Magallana gigas*) and the Pharidae (American jackknife clams, *Ensis leei*) (Kloepper et al. 2022). In contrast, groups containing native species, such as the Tellinidae ( *Macoma balthica* and *Fabulina fabula* ), are revealed as clear losers. In general, we find a positive trend for the Polychaeta, whereas the Bivalvia remain neutral. This overall shift in macrozoobenthic communities towards a more polychaete‐dominated community was also described by Eriksson et al. (2010) who found these shifts to be also reflected by higher trophic levels in shifts in bird communities. However, this general increase in Polychaeta is not uniform across all families. The polychaete families showing the strongest declines, such as the Ampharetidae, Magelonidae, and Polynoidae, are mostly less mobile or sessile carnivores, filter feeders, or subsurface deposit feeders (Fauchald and Jumars 1979). In contrast, those showing positive trends, such as the Pectinariidae and Cirratulidae, are highly mobile surface deposit feeders or carnivores (Fauchald and Jumars 1979). Less mobile species are more susceptible to sediment dynamics and natural bottom perturbations (Meijer et al. 2023), which impose similar stress gradients as human‐induced bottom perturbations like bottom trawling and promote carrion feeding species (van Denderen et al. 2015; McLaverty et al. 2024). Shifts in functional groups of macrozoobenthic species might have been induced by the increasing intensity of human‐induced bottom perturbations in the system (Kloepper et al. 2017, 2022). Notably, we find losers among functionally important species for the stability of coastal ecosystems, such as seagrasses and salt marsh plants (Mcleod et al. 2011). Combining functional and taxonomic aspects to identify winners and losers may highlight urgent conservation needs for threatened ecosystem functions and their associated species, thus allowing for early management interventions to preserve ecological integrity.

### 4.2 Methodological Considerations

The inaccessibility of monitoring data from governmental programs and scientific institutions remains a major obstacle to large‐scale analyses of population trends (Eriksson and Hillebrand 2019). Although the recently developed BioTIME database (Dornelas et al. 2018) collects global biodiversity time series data as a community‐led and open‐source approach, many relevant time series are still missing. This limitation is also mirrored in our analyses, where, despite considerable efforts, we could not access broad data for the Danish part of the Wadden Sea and other known monitoring programs. Moreover, the apparent spatial clustering of sampling stations might introduce a bias through spatial autocorrelation of population trends (Folmer et al. 2014), especially for more mobile species. Marine mammals are one such highly mobile species group that is also missing within our dataset. Only three species of marine mammals occur in the Wadden Sea, of which the harbour porpoise is more a guest than a resident. The population trends of both the harbour and grey seal populations are detailed in the QSR (Unger et al. 2022). The report highlights the recovery of harbour and grey seal populations following severe declines caused by large‐scale hunting in the mid‐20th century. Currently, both seal species present the highest population sizes since the beginning of monitoring. Nevertheless, in addition to the detailed trends on marine mammals in the QSR, we can conclude drastic changes for many species groups in the Wadden Sea based on the current data.

Our findings indicate that the probability of finding a population trend increases with the length of the time series, and thus, short time series may fail to detect changes. This is in line with previous studies on species diversity trends for which the duration‐related bias was shown to underestimate diversity loss (Zhang et al. 2021; Kuczynski et al. 2023). Minimum periods to reliably detect changes are estimated to range between 5 and 30 years, depending on the taxonomic group, with generally a minimum of 10 years (White 2019). Still, these have been estimated from vertebrate populations, and invertebrate species with faster life cycles require shorter periods (Rueda‐Cediel et al. 2015; Wauchope et al. 2019). Moreover, the detection of trends from short‐term time series is quite reliable in terms of sign direction, though the scale of the trend does require longer periods to be estimated reliably (Wauchope et al. 2019). The time series used in the present study have an average length of 32 years but also include three time series shorter than 10 years. These comprise a time series on salt marsh plants, one on phytoplankton, and a short time series of 5 years for the only available zooplankton data. This reflects the general lack of area‐wide monitoring for this species group until recent efforts (Jak and Slijkerman 2023). However, even average durations do not capture important structural changes such as the increasing eutrophication until the 1980s (Kloepper et al. 2017), which is known to have dramatically decreased seagrass meadows (Burkholder et al. 2007) and influenced phytoplankton community structure (Philippart et al. 2000; van Beusekom et al. 2019). Species with fast life cycles, like phytoplankton, have high within‐year variability that might mask long‐term structural changes. To overcome this, we have aggregated the community data to annual means, similar to Antonucci Di Cavalho et al. (2023). Nevertheless, asynchronous sampling by different programmes for species with fast life cycles complicates comparisons. Additionally, a common limitation in time series data is the lack of information on rare species whose capturing highly depends on the sampling effort (Bunge and Fitzpatrick 1993; Chase and Knight 2013) and cannot be reliably incorporated in trend assessments. This is also partly due to changes in taxonomic expertise over the years and between programs. We have standardised taxonomy between the datasets, but this does not remove initial misidentification, which might influence generalisations beyond rare species and influence long‐term trends. To overcome this, we have limited the analyses to higher‐level taxonomic groups and have excluded rare species from our analysis based on the criterion that a species must have been present in five data points to calculate regressions. These limitations highlight the need for standardised monitoring methods across borders and validation of taxonomic resolution (Antonucci Di Cavalho et al. 2023).

This systematic assessment of population trends complements biodiversity analyses focusing on classic diversity metrics or selected species. IUCN Red List and LPI assessments are specifically aimed at identifying populations of conservation concern. However, IUCN Red List and LPI assessments often focus on key or endangered species and do not aim to identify winners alongside losers (Finn et al. 2023). Additionally, the LPI assessment focuses solely on vertebrate populations (Loh et al. 2005), ignoring functionally important parts of the ecosystem, such as invertebrates and primary producers, which often are more directly affected by environmental change (Behrenfeld et al. 2006; Prather et al. 2013). While diversity metrics and population trends of selected species are easily communicated, they may overlook changes in functionally important but neglected groups, resulting in distorted perceptions of the effects of biodiversity change (Lamb et al. 2009). Our analysis does not replace other assessments, as it comes with its own advantages and disadvantages (Table 2). However, using the Wadden Sea as a case study, we show that this systematic and normalized approach can provide important additional insights.

**TABLE 2:** Comparison of assessment approaches.

Approach	Advantages	Disadvantages
Classical biodiversity assessment: e.g., species richness, Shannon or Simpson indices	Directly comparable between areas and applicable to situations without abundance data	Does not give insight into turnover of communities or which species respond to changes (Hillebrand et al. 2018) Susceptible to immigration/extinction debt (Jackson and Sax 2010)
Assessment of Trilateral Wadden Sea Monitoring Programme (TMAP): Mostly based on selected species trends	Comprehensive assessment of many components of the Wadden Sea ecosystem (holistic view)	Differences in assessment strategy between taxonomic groups (and thus, expert groups), no direct comparison of changes possible Some taxonomic groups are overlooked (e.g., phytoplankton communities, zooplankton) Selected species trends (e.g., for birds) mask the change in neglected species
IUCN Red List of Species: Analysis and categorisation of extinction risk based on population size and geographic range	Practical value: Aids in informing conservation priorities (Hoffmann et al. 2008)	Only available for a few taxonomic groups, high number of species classified as “Data Deficient” which may neglect them from conservation planning (Borgelt et al. 2022) Time consuming process requiring a lot of data Classically focuses on “losers” rather than “winners” (although improvements are on the way) (Finn et al. 2023) Criteria are not universally applicable across ecological contexts, thus inconsistencies between national and global assessments may arise which need to be connected with special care (Karam‐Gemael et al. 2020) Static categories (rather than a dynamic assessment)
Living Planet Index (LPI): Population trends for assessing conservation status of selected species groups	Can be used on multiple spatial scales e.g., national, regional, and international (Currie et al. 2022)	Only considers vertebrates (Loh et al. 2005) Focus on “losers” and missing “winners” (Finn et al. 2023), thus no holistic assessment Extreme population trends and random fluctuations can skew global LPI (Buschke et al. 2021)
Generalisation of population trends: (with time‐specific analysis)	Holistic view on diversity in terms of including all taxonomic groups (some of which are underrepresented in current assessments) Allows direct comparisons between taxonomic groups Allows for identification of “winners and losers” Role of species identities can be interpreted (e.g., non‐native species) Identification of critical time points of re‐organisation Dynamic assessment	Depends on spatial scale, monitoring effort and taxonomic resolution of monitoring Probability of finding a trend depends on the length of the time series (as shown in Figure S3) Excludes rare species Trends are dependent on baselines. Introduced species can only increase whereas species at carrying capacity can only stay neutral or decrease

## 5 Conclusions

In contrast to static conservation categories (e.g., IUCN), population trend analyses offer a dynamic assessment of biodiversity change that can serve as early warning signals for local extinctions (Finn et al. 2023). Except for marine mammals and specific microbes (microphytobenthos, bacteria), the assessment presented here covers all species groups that comprise the Wadden Sea ecosystem and identifies both winners and losers across time. Losers are phylogenetically related, hinting at shared life history traits that may explain vulnerabilities to environmental change. Non‐native species are identified as winners, especially among the macrozoobenthos. Moreover, the majority of losers are phytoplankton, which are currently not addressed in monitoring programs beyond bulk biomass and thus overlooked from a species perspective in assessment strategies. Therefore, signs of biodiversity change may be represented by less charismatic organism groups not usually included in management schemes. Finally, our assessment reveals clear losses in the functionality of the ecosystem, indicated by population declines in fish groups that use the Wadden Sea as a nursery area, bird groups that use the Wadden Sea as a feeding and breeding area, and plant groups that stabilize the coastline. This work can thus serve as a stepping stone for further analyses focusing on functional or food web perspectives and quantitatively linking pressures with the shown biological reorganization. This holistic approach captures the dynamic and interconnected nature of seascapes and provides a near‐complete representation of the regional biodiversity status that goes beyond the assessment of key indicator species, which may help to guide ecosystem‐wide conservation and management strategies.

## Author Contributions

**Anika Happe:** conceptualization, data curation, formal analysis, methodology, visualization, writing – original draft, writing – review and editing. **Kasper J. Meijer:** conceptualization, data curation, formal analysis, methodology, visualization, writing – original draft, writing – review and editing. **Jan‐Claas Dajka:** methodology, writing – review and editing. **Oscar Franken:** methodology, writing – review and editing. **Holger Haslob:** data curation, methodology, writing – review and editing. **Laura L. Govers:** writing – review and editing. **Michael Kleyer:** data curation. **Annebelle C. M. Kok:** methodology, writing – review and editing. **Lucie Kuczynski:** writing – review and editing. **Kertu Lõhmus:** data curation. **Sancia E. T. van der Meij:** data curation, methodology, writing – review and editing. **Han Olff:** data curation, methodology, writing – review and editing. **Lena Rönn:** data curation, writing – review and editing. **Alexey Ryabov:** methodology, writing – review and editing. **Anne F. Sell:** data curation, methodology, writing – review and editing. **David W. Thieltges:** writing – review and editing. **Britas Klemens Eriksson:** conceptualization, data curation, methodology, writing – review and editing. **Helmut Hillebrand:** conceptualization, data curation, methodology, writing – review and editing.

## Conflicts of Interest

The authors declare no conflicts of interest.

## Supporting information


**Figure S1.** (A) Schematic workflow for the vote count of the trends. (B) Schematic workflow for the chosen error distribution for the models used in the vote count.
**Figure S2.** Schematic overview of the temporal trends classification. The probability of positive or negative trends is always considered against the probability of the trend being neutral. In case the probability of both a negative and positive trend is below 0.5, the overall trend is neutral. In case the probabilities are higher than 0.5, the overall trend is that with the highest probability. In case both trend probabilities overlap, there is an equal probability of positive and negative trends which cancel each other out.
**Figure S3.** (A) Probability of detecting a trend with increasing monitoring time; (B) Probability of a model meeting assumptions of a Gaussian, Poisson, or negative binomial error distribution using linear or polynomial regression with increasing monitoring time.
**Figure S4.** Dendrogram of the meta‐analysis results (coloured branches). The colour indicates an overall significantly positive trend (green), negative trend (orange), or a non‐significant overall trend (blue). The labels refer to the genus of the branch. The estimates and 95% confidence intervals for each taxonomic level are presented in Tables S4–S8.
**Figure S5.** Winners and losers in the class of Aves (birds). Estimates and 95% confidence intervals (CI) were derived from the meta‐analysis to identify winners (green) and losers (orange) on the phylogenetic level of family. The significance (*p* < 0.05) is indicated by CI not crossing the dashed line at 0.
**Figure S6.** Winners and losers in the ecosystem component of zooplankton. Estimates and 95% confidence intervals (CI) were derived from the meta‐analysis to identify winners (green) and losers (orange) on the phylogenetic level of family. Significance (*p* < 0.05) is indicated by CI not crossing the dashed line at 0.
**Figure S7.** Winners and losers in the ecosystem component of macrozoobenthos. Estimates and 95% confidence intervals (CI) were derived from the meta‐analysis to identify winners (green) and losers (orange) on the phylogenetic level of family. Significance (*p* < 0.05) is indicated by CI not crossing the dashed line at 0.
**Figure S8.** Winners and losers in the ecosystem component of fish. Estimates and 95% confidence intervals (CI) were derived from the meta‐analysis to identify winners (green) and losers (orange) on the phylogenetic level of family. Significance (*p* < 0.05) is indicated by CI not crossing the dashed line at 0.
**Table S1.** Taxonomic information on all taxa included in the datasets and the number of sampling stations for which each taxa was recorded.
**Table S2.** Number of entries for each ecosystem component and phylogenetic level. Entries identified only to genus level were included as “Genera sp.” at species level. The ecosystem component of “Plants” includes both salt marsh plants and seagrasses. Macroinvertebrates show a lower number of genera than families as three genera could not be assigned. Abbreviations are as follows: Phytoplankton (Phytopl.), macrozoobenthos (MZB), zooplankton (Zoopl.).
**Table S3.** Multinomial model output for the temporal trends. Taxa are ordered by taxonomic level. In case the polynomial model was a better fit, 2nd order positive and negative coefficients are also given. Significant *p*‐values (*p* < 0.05) are indicated in bold.
**Table S4.** Meta‐analysis results used to assign the winner and loser status on phylum level. The meta‐analysis was run on the full dataset.
**Table S5.** Meta‐analysis results used to assign the winner and loser status on class level. The meta‐analysis was run on the full dataset.
**Table S6.** Meta‐analysis results used to assign the winner and loser status on order level. The meta‐analysis was run on the full dataset. MZB stands for macrozoobenthos. The subsets of the meta‐analyses were grouped as follows: (i) plants, (ii) phytoplankton, (iii) fish, (iv) birds and (v) macrozoobenthos and zooplankton.
**Table S7.** Meta‐analysis results used to assign the winner and loser status on family level. The meta‐analysis was run on the full dataset. MZB stands for macrozoobenthos. The subsets of the meta‐analyses were grouped as follows: (i) plants, (ii) phytoplankton, (iii) fish, (iv) birds and (v) macrozoobenthos and zooplankton.
**Table S8.** Meta‐analysis results used to assign the winner and loser status on genus level. The meta‐analysis was run on the full dataset. MZB stands for macrozoobenthos. The subsets of the meta‐analyses were grouped as follows: (i) plants, (ii) phytoplankton, (iii) fish, (iv) birds and (v) macrozoobenthos and zooplankton.
**Table S9.** List of PANGAEA data sources for stownet fish data counts in the Eastfrisian Wadden Sea.

## Data Availability

The data and code that support the findings of this study are openly available in the dataverseNL (DANS) repository at https://doi.org/10.34894/LJNSYR. Long‐term saltmarsh data were obtained from DataverseNL at https://doi.org/10.34894/FASXGR (Schiermonnikoog), and https://doi.org/10.5061/dryad.gxd2547z3 (Mellum and Spiekeroog). Phytoplankton monitoring data were obtained from Zenodo at https://doi.org/10.5281/zenodo.8192381. Macrozoobenthos data were obtained from the Balgzand long‐term monitoring program via NIOZ at https://doi.org/10.25850/nioz/7b.b.sh. A list of the sources of fish data (stow net) data used in this study can be found in Table S9. Demersal Young Fish Survey (DYFS) data were obtained from the ICES DATRAS database at https://gis.ices.dk/geonetwork/srv/metadata/87ea4d4d‐6798‐4c06‐b001‐7b59ad1ea916. Macrozoobenthos data were obtained from MUDAB at https://geoportal.bafg.de/MUDABAnwendung/ (Germany) and AquaDesk at https://live.aquadesk.nl (Dutch Rijkswaterstaat MWTL). Phytoplankton data were obtained from MUDAB at https://geoportal.bafg.de/MUDABAnwendung/. Zooplankton data were obtained from the Lower Saxony Water Management Agency at https://geoportal.bafg.de/MUDABAnwendung. Bird data for the Netherlands were obtained from Netwerk Ecologische Monitoring at https://stats.sovon.nl/stats/gebied/1000001.
