# Supplementary material for: Synthesis of Population Trends Reveals Seascape‐Wide Reorganisation of Biodiversity From Microalgae to Birds
Source: Glob Chang Biol. 2025 Jun 18;31(6):e70298. doi: 10.1111/gcb.70298 (PMC12175116; doi:10.1111/gcb.70298)
Supplement: Supplementary file 1 — Figure S1. (A) Schematic workflow for the vote count of the trends. (B) Schematic workflow for the chosen error distribution for the models used in the vote count. Figure S2. Schematic overview of the temporal trends classification. The probability of positive or negative trends is always considered against the probability of the trend being neutral. In case the probability of both a negative and positive trend is below 0.5, the overall trend is neutral. In case the probabilities are higher than 0.5, the overall trend is that with the highest probability. In case both trend probabilities overlap, there is an equal probability of positive and negative trends which cancel each other out. Figure S3. (A) Probability of detecting a trend with increasing monitoring time; (B) Probability of a model meeting assumptions of a Gaussian, Poisson, or negative binomial error distribution using linear or polynomial regression with increasing monitoring time. Figure S4. Dendrogram of the meta‐analysis results (coloured branches). The colour indicates an overall significantly positive trend (green), negative trend (orange), or a non‐significant overall trend (blue). The labels refer to the genus of the branch. The estimates and 95% confidence intervals for each taxonomic level are presented in Tables S4–S8. Figure S5. Winners and losers in the class of Aves (birds). Estimates and 95% confidence intervals (CI) were derived from the meta‐analysis to identify winners (green) and losers (orange) on the phylogenetic level of family. The significance (p < 0.05) is indicated by CI not crossing the dashed line at 0. Figure S6. Winners and losers in the ecosystem component of zooplankton. Estimates and 95% confidence intervals (CI) were derived from the meta‐analysis to identify winners (green) and losers (orange) on the phylogenetic level of family. Significance (p < 0.05) is indicated by CI not crossing the dashed line at 0. Figure S7. Winners and losers in the ecosystem component of macro [file GCB-31-e70298-s001.docx]

# Supplementary material to: Synthesis of population trends from microalgae to birds captures sea-scape wide biodiversity change

Authors:

Anika Happe*^1^, Kasper J. Meijer*^2^, Jan-Claas Dajka^3,4,1^, Oscar Franken^5,2^, Holger Haslob^6^, Laura L. Govers^2,5^, Michael Kleyer^7^, Annebelle C.M. Kok^2^, Lucie Kuczynski^1,8^, Kertu Lõhmus^7^, Sancia E.T. van der Meij^2,9^, Han Olff^2^, Lena Rönn^10^, Alexey Ryabov^1,4^, Anne F. Sell^6^, David W. Thieltges^5,2^, Britas Klemens Eriksson**^2^ and Helmut Hillebrand**^1,3,4^

* Anika Happe and Kasper J. Meijer should be considered joint first author

** Britas Klemens Eriksson and Helmut Hillebrand should be considered joint senior Affiliations:

1) Institute for Chemistry and Biology of the Marine Environment (ICBM), School of Mathematics and Science, Carl von Ossietzky Universität Oldenburg, Ammerländer Heerstraße 114-118, 26129 Oldenburg, Germany

2) Groningen Institute for Evolutionary Life-Sciences, University of Groningen, Nijenborgh 7, 9747 AG Groningen, The Netherlands

3) Helmholtz-Institute for Functional Marine Biodiversity at the University of Oldenburg [HIFMB], Ammerländer Heerstrasse 231, 26129 Oldenburg

4) Alfred Wegener Institute, Helmholtz-Centre for Polar and Marine Research [AWI], Bremerhaven

5) Department of Coastal Systems, NIOZ Royal Netherlands Institute for Sea Research, Den Burg, The Netherlands

6) Thünen Institute of Sea Fisheries, Herwigstraße 31, 27572 Bremerhaven, Germany

7) Institute of Biology and Environmental Sciences, School of Mathematics and Science, Carl von Ossietzky Universität Oldenburg, Ammerländer Heerstraße 114-118, 26129 Oldenburg, Germany

8) UMR ENTROPIE, Lucie Kuczynski IRD, IFREMER, CNRS, University of La Reunion, University of New Caledonia, Noumea, New Caledonia

9) Naturalis Biodiversity Center, Darwinweg 2, 2333 CR Leiden, The Netherlands

10) Lower Saxony Water Management, Coastal and Nature Protection Agency (NLWKN, Brake-Oldenburg), Im Dreieck 12, 26127 Oldenburg, Germany

##

## Detailed description of the study system

The Wadden Sea is the world’s largest tidal flat system and extends along the coasts of the Netherlands, Germany and Denmark (Fig. 2a). It is characterised by strong tides and water exchange, high turbidity, variations in salinity, and a high seasonality in temperature (Heron et al., 2020; Rönn et al., 2023). The Dutch Wadden Sea Conservation Area and the German Wadden Sea National Parks of Lower Saxony and Schleswig-Holstein were designated as a UNESCO World Heritage site in 2009. This was later followed by the Hamburg Wadden Sea National Park in 2011 and the Danish Wadden Sea in 2014. The Wadden Sea is of international significance due to its pivotal role as a staging, wintering and breeding area for many migratory birds and as nursery grounds for many fish species (Boere & Piersma, 2012; Wolff, 2013) and brown shrimp, *Crangon crangon* (Kuipers and Dapper, 1984). The Wadden Sea finds legal protection through the Natura 2000 framework within Europe (Bastmeijer et al., 2023). However, the actual implementation and efficacy of this protection is more complex (Bastmeijer et al., 2023; Meijer et al., 2024).

The main anthropogenic activities in the Wadden Sea include tourism, fisheries focused on the North Sea brown shrimp (*Crangon crangon*), the blue mussel (*Mytilus edulis*) and the common cockle (*Cerastoderma edule*), and shipping as well as its proximity to intensely used shipping routes towards major ports and the Kiel Canal (Kloepper et al., 2024). Further, regional stressors encompass increasing temperature, accelerated sea-level rise, and an increase in extreme temperature events with impacts on the hydromorphodynamic development of the islands, halligen, deltas, and back-barrier areas (Kloepper et al., 2024) and subsequently for the ecosystems of the associated habitats. Additionally, eutrophication remains one of the biggest challenges for this coastal ecosystem, although the annual nutrient loadings show a decreasing trend since the 1990s (Kloepper et al., 2024; Rönn et al., 2023).

### References

Bastmeijer, K., Boerema, L., Gilissen, H. K., Kistenkas, F., Miltenburg, L., van Rijswick, M., Trouwborst, A., Verschuuren, J., & Zwier, W. (2023). *De Europees- en internationaalrechtelijke status van de Waddenzee. Een analyse van de relevantie van EU-richtlijnen en internationale verdragen voor de bescherming en het beheer van de Waddenzee met een doorkijk naar de Nederlandse implementatie*. Waddenacademie.

Boere, G. C., & Piersma, T. (2012). Flyway protection and the predicament of our migrant birds: A critical look at international conservation policies and the Dutch Wadden Sea. *Ocean & Coastal Management*, *68*, 157–168. [https://doi.org/10.1016/j.ocecoaman.2012.05.019](NULL)

Heron, S. F., Day, J. C., Zijlstra, R., Engels, B., Weber, A., Marencic, H., & Busch, J. A. (2020). Climate Risk Assessment for Wadden Sea World Heritage property: Application of the Climate Vulnerability Index – Outstanding Universal Value (OUV) Vulnerability (Workshop report). Common Wadden Sea Secretariat (CWSS), Wilhelmshaven, Germany.

Kloepper S., Bostelmann A., Bregnballe T., Busch J.A., Buschbaum C., Deen K., Domnick A., Gutow L., Jensen K., Jepsen N., Luna S., Meise K., Teilmann J. & van Wezel A. (2024) Wadden Sea Quality Status Report. Common Wadden Sea Secretariat, Wilhelmshaven, Germany. Downloaded 22.05.2024. qsr.waddensea-worldheritage.org

Kuipers, B.R., & Dapper, R. (1984). Nursery function of Wadden Sea tidal flats for the brown shrimp *Crangon crangon*. *Marine Ecology Progress Series*, *17*, 171–181.

Meijer, K. J., Franken, O., Witte, S., Holthuijsen, S. J., van der Heide, T., Govers, L. L., & Olff, H. (2024). Hotspots in peril: Misalignment of conservation efforts and ecological values in a shallow coastal sea. *People and Nature*, *00*, 1–20. [https://doi.org/10.1002/pan3.10757](NULL)

Rönn, L. and others 2023. Harmonisation of the phytoplankton assessment in the German and Dutch Wadden Sea. Interreg V A project “Wasserqualität - Waterkwaliteit” - synthesis report. Report prepared on behalf of NLWKN and Rijkswaterstaat, Oldenburg/Lelystad, 2023.

Wolff, W. J. (2013). Ecology of the Wadden Sea: Research in the past and challenges for the future. *Journal of Sea Research*, *82*, 3–9. [https://doi.org/10.1016/j.seares.2013.03.006](NULL)

Meta-analysis: Winners and losers on family level

Negative trends in the fish (Teleostei) are especially found in the families Gadidae (cods) and Pleuronectidae (righteye flounders). Threskiornithidae (spoonbills) and Phalacrocoracidae (cormorants) families show the strongest positive trends within the Aves (Fig. S5). The only zooplankton family with a significantly positive trend direction is Oithonidae (small cyclopoid copepods). In contrast, Centropagidae, Paracalanidae (two classes of calanoid copepods), and Mesodiniidae (with the ciliate species *Mesodinium rubrum*) show clear negative trends (Fig. S6). The macrozoobenthos (Fig. S7) shows winners for diverse groups such as cumaceans (Bodotriidae), oysters (Ostreidae; specifically the Pacific oyster *Magallana gigas*), razor or jackknife clams (Pharidae), thoracotreme crabs (Varunidae, only represented by *Hemigrapsus takanoi*) but also annelids (Naididae, Pectinariidae, Cirratulidae) and echinoderms (Ophiuridae). Nevertheless, for many of these groups also losers were found (Fig. S7), such as for polychaetes (e.g., Ampharetidae, Nephtyidae, Magelonidae), molluscs (e.g., Retusidae, Tonicellidae, Tellinidae) and certain crustaceans (Mysidae).

###


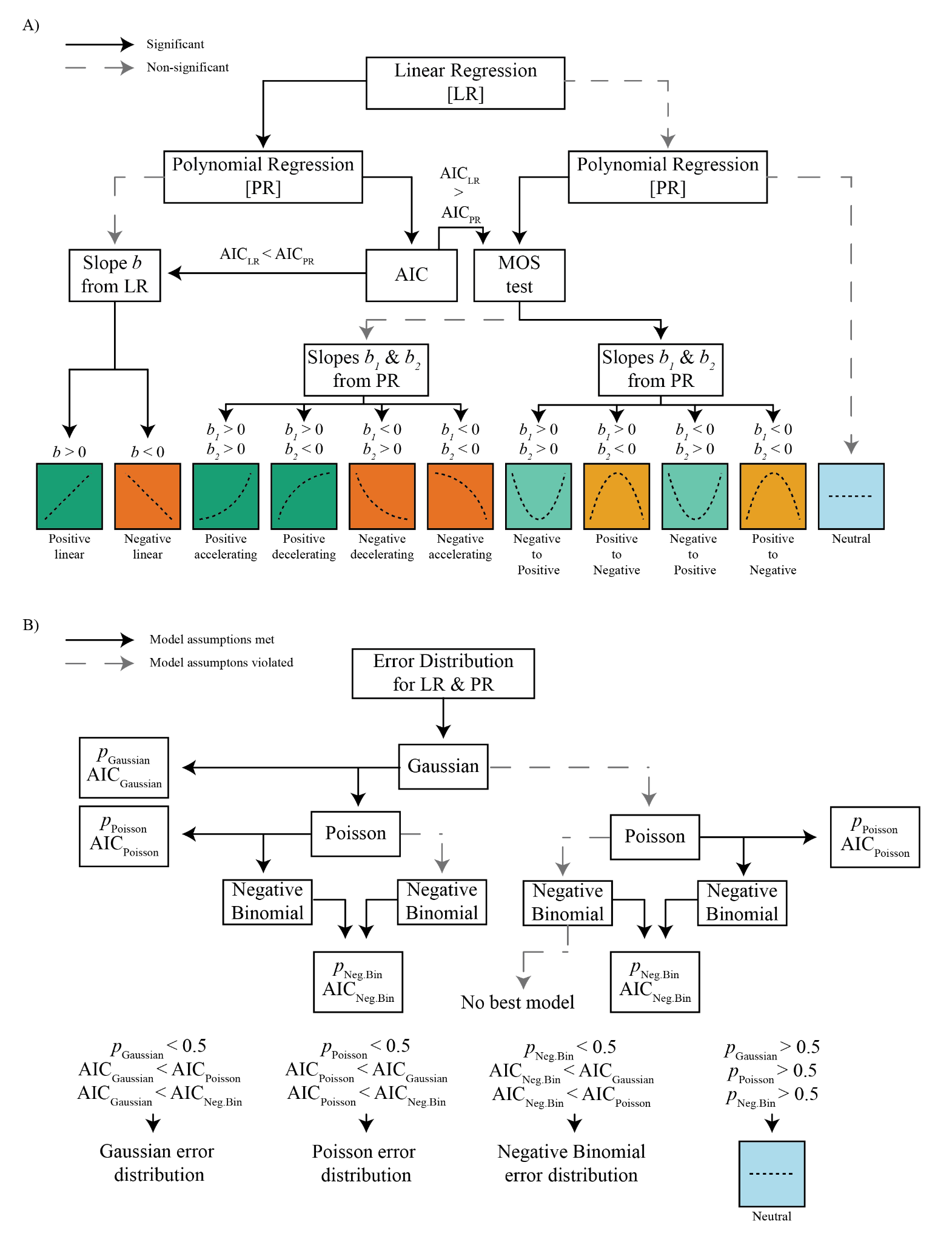


#### **Fig. S1.** A) Schematic workflow for the vote count of the trends. B) Schematic workflow for the chosen error distribution for the models used in the vote count

####
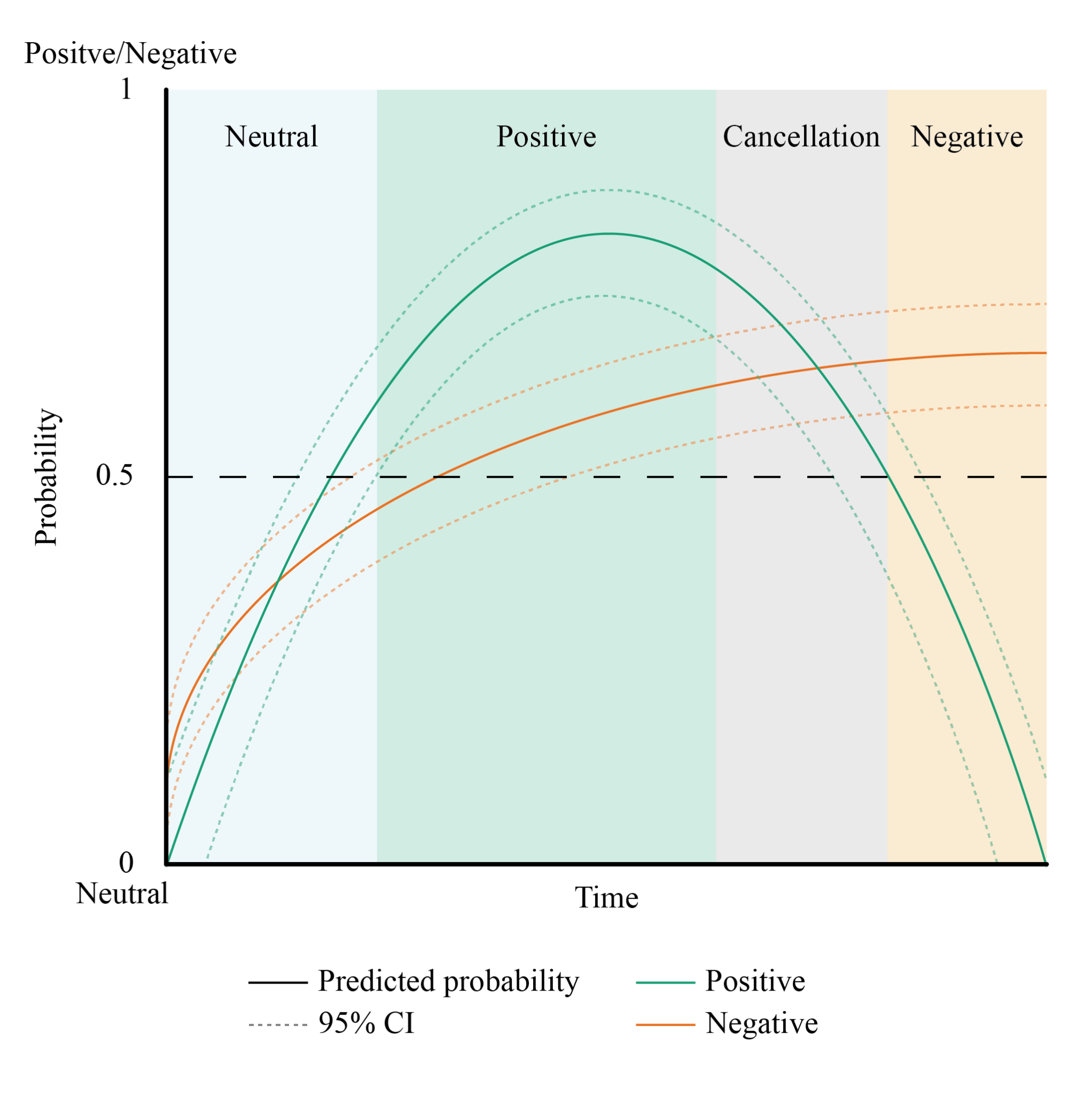
**Fig. S2.** Schematic overview of the temporal trends classification. The probability of positive or negative trends is always considered against the probability of the trend being neutral. In case the probability of both a negative and positive trend is below 0.5, the overall trend is neutral. In case the probabilities are higher than 0.5, the overall trend is that with the highest probability. In case both trend probabilities overlap, there is an equal probability of positive and negative trends which cancel each other out.

#### **
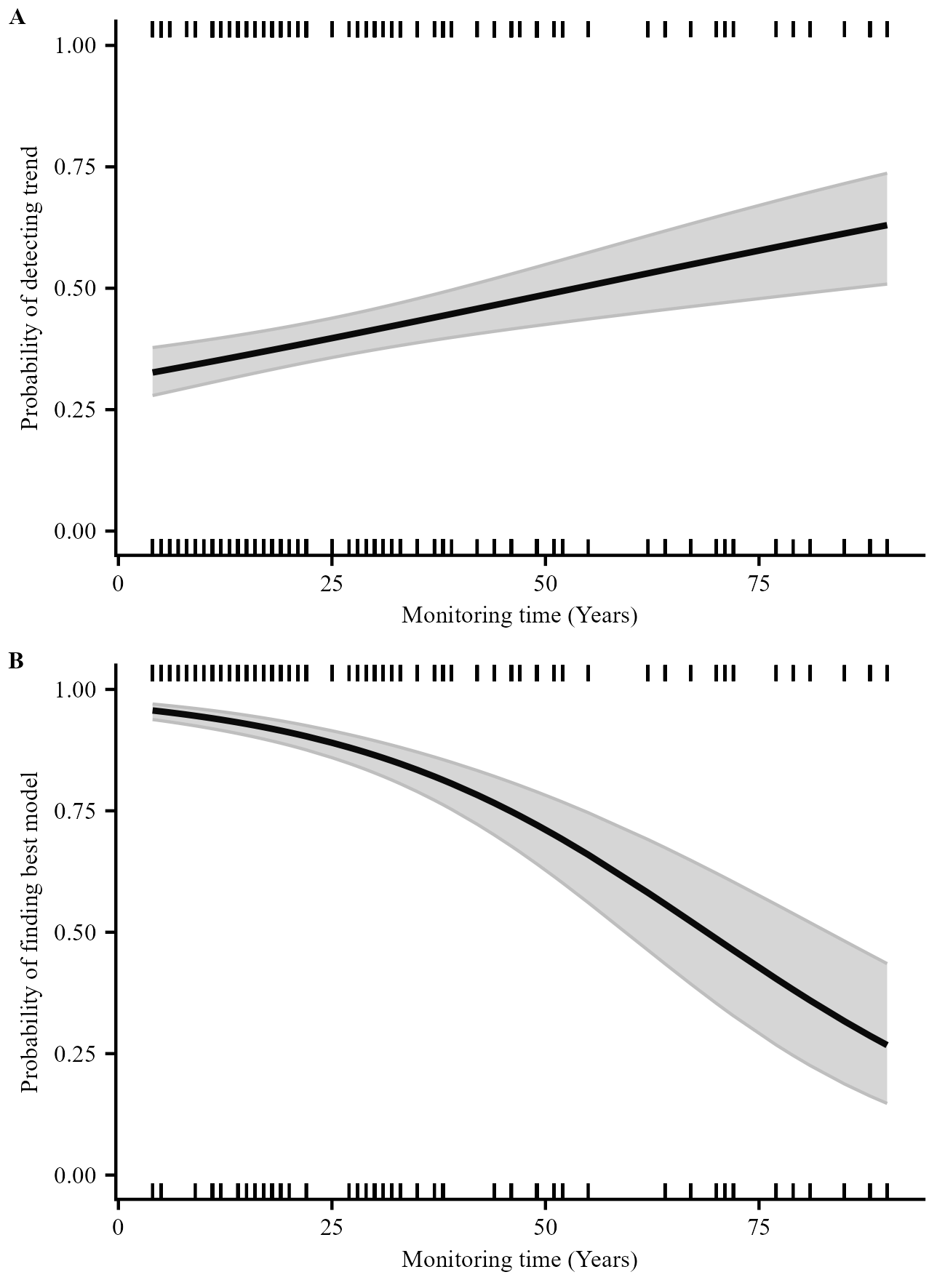
Fig. S3.** A) Probability of detecting a trend with increasing monitoring time; B) Probability of a model meeting assumptions of a Gaussian, Poisson or negative binomial error distribution using linear or polynomial regression with increasing monitoring time.


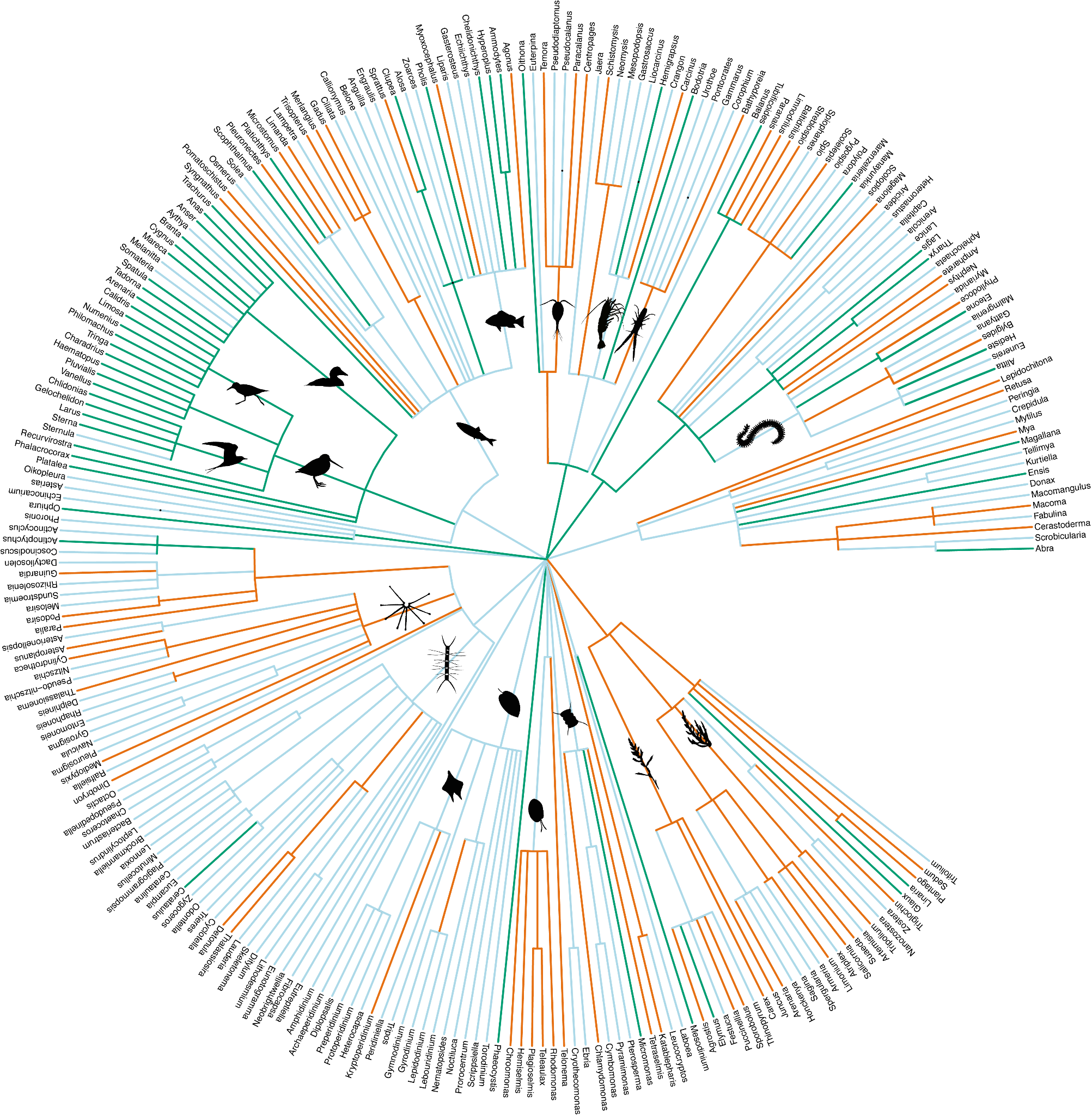
**Fig. S4.** Dendrogram of the meta-analysis results (coloured branches). The colour indicates an overall significantly positive trend (green), negative trend (orange) or a non-significant overall trend (blue). The labels refer to the genus of the branch. The estimates and 95% confidence intervals for each taxonomic level are presented in Table S4-8.


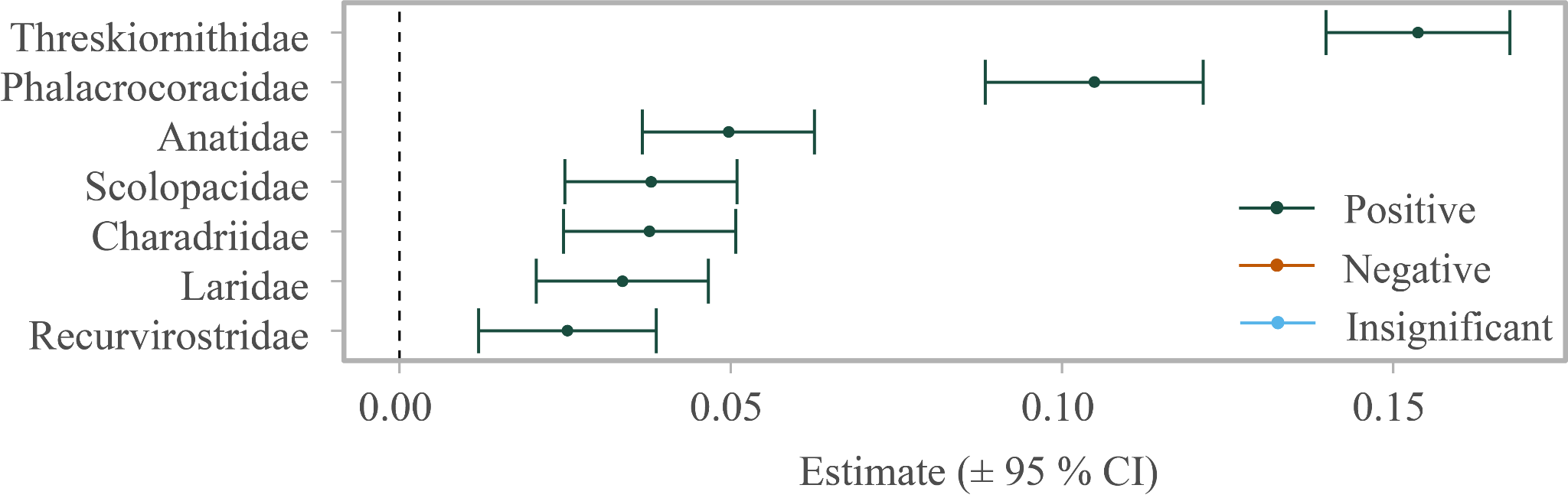
**Fig. S5.** Winners and losers in the class of Aves (birds). Estimates and 95 % confidence intervals (CI) were derived from the meta-analysis to identify winners (green) and losers (orange) on the phylogenetic level of family. Significance (p < 0.05) is indicated by CI not crossing the dashed line at 0.

#### **
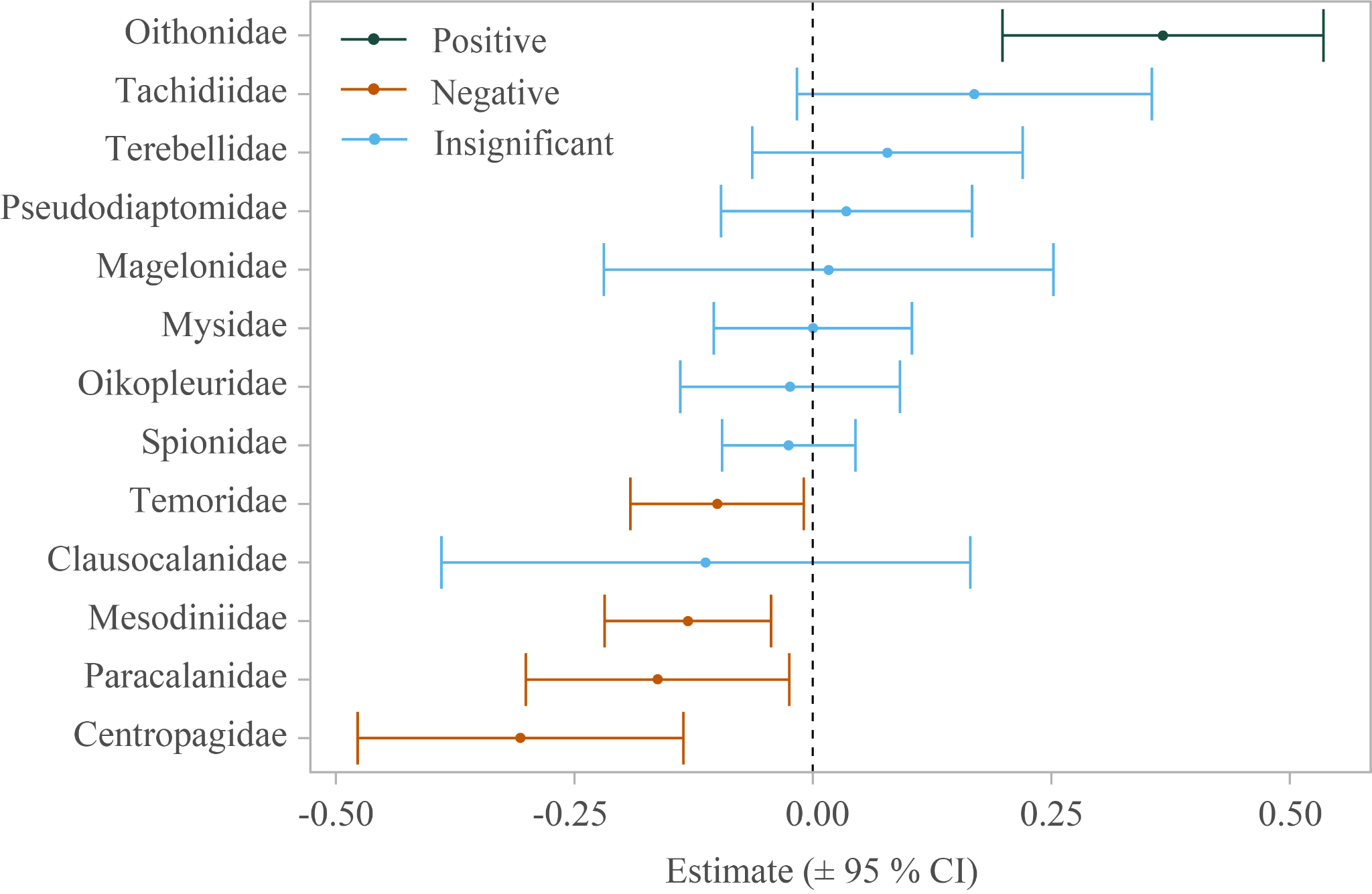
**

**Fig. S6.** Winners and losers in the ecosystem component of zooplankton. Estimates and 95 % confidence intervals (CI) were derived from the meta-analysis to identify winners (green) and losers (orange) on the phylogenetic level of family. Significance (p < 0.05) is indicated by CI not crossing the dashed line at 0.


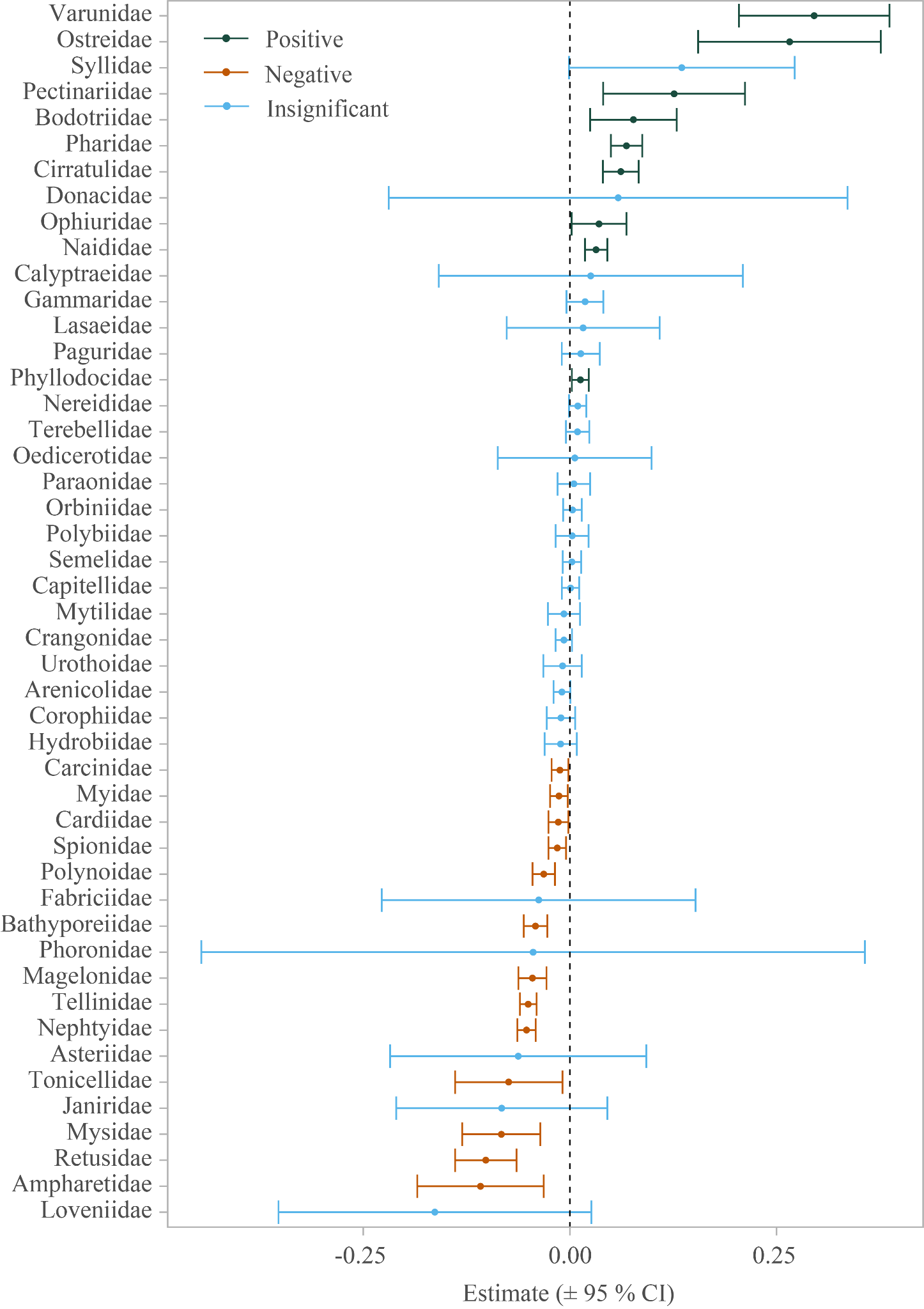


#### **Fig. S7.** Winners and losers in the ecosystem component of macrozoobenthos. Estimates and 95 % confidence intervals (CI) were derived from the meta-analysis to identify winners (green) and losers (orange) on the phylogenetic level of family. Significance (p < 0.05) is indicated by CI not crossing the dashed line at 0.


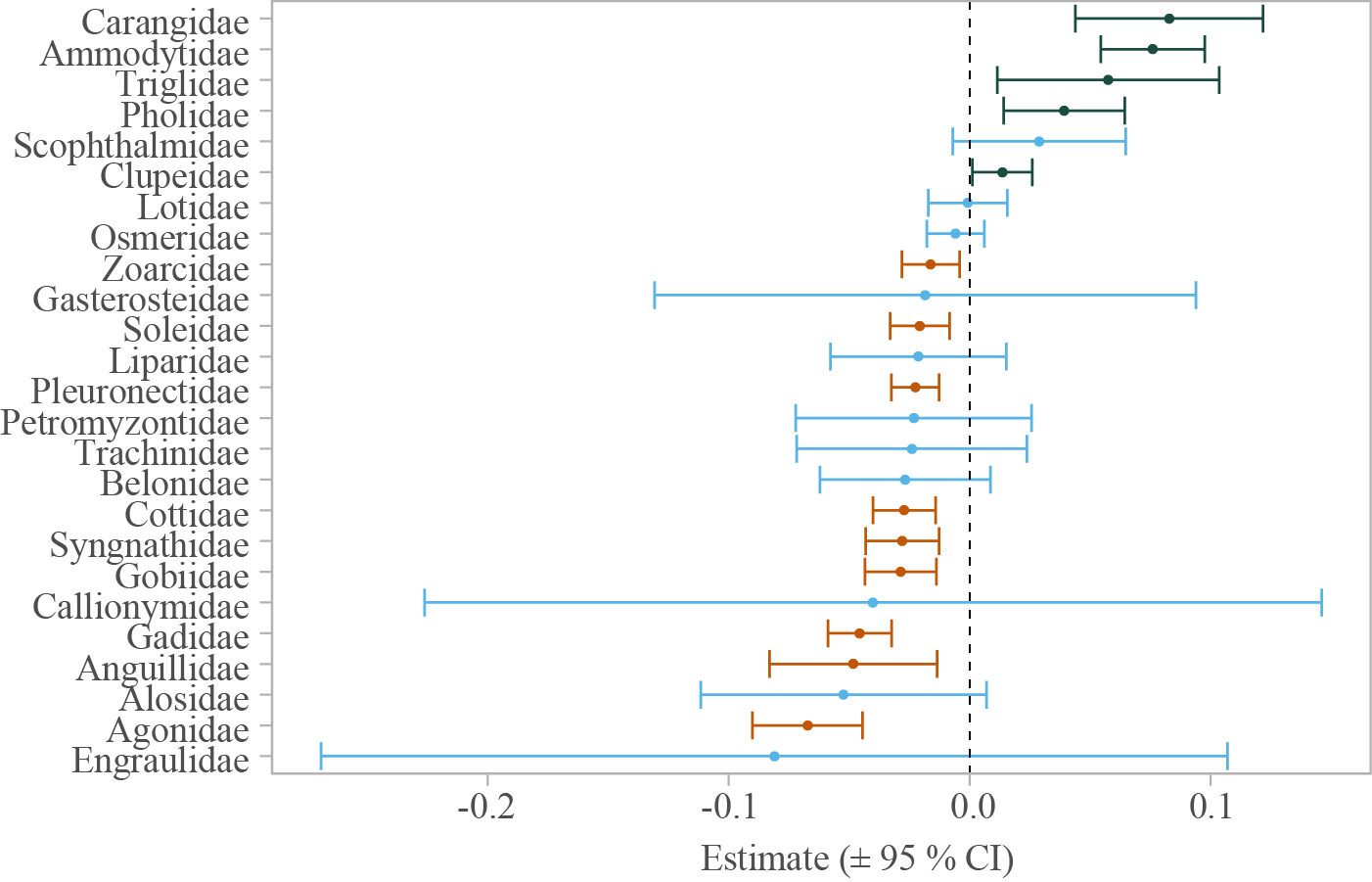


#### **Fig. S8.** Winners and losers in the ecosystem component of fish. Estimates and 95 % confidence intervals (CI) were derived from the meta-analysis to identify winners (green) and losers (orange) on the phylogenetic level of family. Significance (p < 0.05) is indicated by CI not crossing the dashed line at 0.

#### **Table S1.** Taxonomic information on all taxa included in the datasets and the number of sampling stations for which each taxa was recorded.

| **Aphia ID** | **Species valid name** | **Group** | **Authority** | **Kingdom** | **Phylum** | **Class** | **Order** | **Family** | **Genus** | **Nr. stations** |
| --- | --- | --- | --- | --- | --- | --- | --- | --- | --- | --- |
| 2036 | Oligochaeta sp. | Macrozoobenthos | Grube, 1850 | Animalia | Annelida | Clitellata | not assigned | not assigned | not assigned | 7 |
| 743898 | *Baltidrilus costatus* | Macrozoobenthos | (Claparède, 1863) | Animalia | Annelida | Clitellata | Tubificida | Naididae | Baltidrilus | 13 |
| 137556 | *Limnodrilus hoffmeisteri* | Macrozoobenthos | Claparède, 1862 | Animalia | Annelida | Clitellata | Tubificida | Naididae | Limnodrilus | 3 |
| 137485 | *Paranais litoralis* | Macrozoobenthos | (Müller, 1784) | Animalia | Annelida | Clitellata | Tubificida | Naididae | Paranais | 3 |
| 137571 | *Tubificoides benedii* | Macrozoobenthos | (d'Udekem, 1855) | Animalia | Annelida | Clitellata | Tubificida | Naididae | Tubificoides | 21 |
| 137577 | *Tubificoides heterochaetus* | Macrozoobenthos | (Michaelsen, 1926) | Animalia | Annelida | Clitellata | Tubificida | Naididae | Tubificoides | 3 |
| 137582 | *Tubificoides pseudogaster* | Macrozoobenthos | (Dahl, 1960) | Animalia | Annelida | Clitellata | Tubificida | Naididae | Tubificoides | 7 |
| 730747 | *Aricidea minuta* | Macrozoobenthos | Southward, 1956 | Animalia | Annelida | Polychaeta | Cirratulida | Paraonidae | Aricidea | 1 |
| 129868 | *Arenicola marina* | Macrozoobenthos | (Linnaeus, 1758) | Animalia | Annelida | Polychaeta | Capitellida | Arenicolidae | Arenicola | 29 |
| 129876 | *Capitella capitata* | Macrozoobenthos | (Fabricius, 1780) | Animalia | Annelida | Polychaeta | Capitellida | Capitellidae | Capitella | 17 |
| 129879 | *Capitella minima* | Macrozoobenthos | Langerhans, 1880 | Animalia | Annelida | Polychaeta | Capitellida | Capitellidae | Capitella | 1 |
| 129211 | *Capitella sp.* | Macrozoobenthos | Blainville, 1828 | Animalia | Annelida | Polychaeta | Capitellida | Capitellidae | Capitella | 3 |
| 129884 | *Heteromastus filiformis* | Macrozoobenthos | (Claparède, 1864) | Animalia | Annelida | Polychaeta | Capitellida | Capitellidae | Heteromastus | 49 |
| 130269 | *Magelona johnstoni* | Macrozoobenthos | Fiege, Licher & Mackie, 2000 | Animalia | Annelida | Polychaeta | not assigned | Magelonidae | Magelona | 8 |
| 130271 | *Magelona mirabilis* | Macrozoobenthos | (Johnston, 1865) | Animalia | Annelida | Polychaeta | not assigned | Magelonidae | Magelona | 7 |
| 130272 | *Magelona papillicornis* | Macrozoobenthos; Zooplankton | Müller, 1858 | Animalia | Annelida | Polychaeta | not assigned | Magelonidae | Magelona | 2 |
| 130537 | *Scoloplos armiger* | Macrozoobenthos | (Müller, 1776) | Animalia | Annelida | Polychaeta | not assigned | Orbiniidae | Scoloplos | 39 |
| 130355 | *Nephtys caeca* | Macrozoobenthos | (Fabricius, 1780) | Animalia | Annelida | Polychaeta | Phyllodocida | Nephtyidae | Nephtys | 2 |
| 130357 | *Nephtys cirrosa* | Macrozoobenthos | Ehlers, 1868 | Animalia | Annelida | Polychaeta | Phyllodocida | Nephtyidae | Nephtys | 8 |
| 130359 | *Nephtys hombergii* | Macrozoobenthos | Savigny in Lamarck, 1818 | Animalia | Annelida | Polychaeta | Phyllodocida | Nephtyidae | Nephtys | 40 |
| 130364 | *Nephtys longosetosa* | Macrozoobenthos | Örsted, 1842 | Animalia | Annelida | Polychaeta | Phyllodocida | Nephtyidae | Nephtys | 2 |
| 129370 | *Nephtys sp.* | Macrozoobenthos | Cuvier, 1817 | Animalia | Annelida | Polychaeta | Phyllodocida | Nephtyidae | Nephtys | 1 |
| 234850 | *Alitta succinea* | Macrozoobenthos | (Leuckart, 1847) | Animalia | Annelida | Polychaeta | Phyllodocida | Nereididae | Alitta | 8 |
| 234851 | *Alitta virens* | Macrozoobenthos | (Sars, 1835) | Animalia | Annelida | Polychaeta | Phyllodocida | Nereididae | Alitta | 1 |
| 130375 | *Eunereis longissima* | Macrozoobenthos | (Johnston, 1840) | Animalia | Annelida | Polychaeta | Phyllodocida | Nereididae | Eunereis | 1 |
| 152302 | *Hediste diversicolor* | Macrozoobenthos | (O.F. Müller, 1776) | Animalia | Annelida | Polychaeta | Phyllodocida | Nereididae | Hediste | 36 |
| 22496 | Nereididae sp. | Macrozoobenthos | Blainville, 1818 | Animalia | Annelida | Polychaeta | Phyllodocida | Nereididae | not assigned | 3 |
| 130616 | *Eteone longa* | Macrozoobenthos | (Fabricius, 1780) | Animalia | Annelida | Polychaeta | Phyllodocida | Phyllodocidae | Eteone | 39 |
| 334512 | *Phyllodoce mucosa* | Macrozoobenthos | Örsted, 1843 | Animalia | Annelida | Polychaeta | Phyllodocida | Phyllodocidae | Phyllodoce | 22 |
| 130735 | *Bylgides sarsi* | Macrozoobenthos | (Kinberg in Malmgren, 1866) | Animalia | Annelida | Polychaeta | Phyllodocida | Polynoidae | Bylgides | 6 |
| 130749 | *Gattyana cirrhosa* | Macrozoobenthos | (Pallas, 1766) | Animalia | Annelida | Polychaeta | Phyllodocida | Polynoidae | Gattyana | 1 |
| 863197 | *Malmgrenia darbouxi* | Macrozoobenthos | (Pettibone, 1993) | Animalia | Annelida | Polychaeta | Phyllodocida | Polynoidae | Malmgrenia | 1 |
| 238200 | *Myrianida prolifera* | Macrozoobenthos | (Müller, 1788) | Animalia | Annelida | Polychaeta | Phyllodocida | Syllidae | Myrianida | 1 |
| 129659 | *Myrianida sp.* | Macrozoobenthos | H. Milne Edwards, 1845 | Animalia | Annelida | Polychaeta | Phyllodocida | Syllidae | Myrianida | 1 |
| 130926 | *Manayunkia aestuarina* | Macrozoobenthos | (Bourne, 1883) | Animalia | Annelida | Polychaeta | Sabellida | Fabriciidae | Manayunkia | 2 |
| 131135 | *Marenzelleria viridis* | Macrozoobenthos | (Verrill, 1873) | Animalia | Annelida | Polychaeta | Spionida | Spionidae | Marenzelleria | 9 |
| 131143 | *Polydora cornuta* | Macrozoobenthos; Zooplankton | Bosc, 1802 | Animalia | Annelida | Polychaeta | Spionida | Spionidae | Polydora | 16 |
| 131170 | *Pygospio elegans* | Macrozoobenthos | Claparède, 1863 | Animalia | Annelida | Polychaeta | Spionida | Spionidae | Pygospio | 36 |
| 131171 | *Scolelepis bonnieri* | Macrozoobenthos | (Mesnil, 1896) | Animalia | Annelida | Polychaeta | Spionida | Spionidae | Scolelepis | 1 |
| 131173 | *Scolelepis foliosa* | Macrozoobenthos | (Audouin & Milne Edwards, 1833) | Animalia | Annelida | Polychaeta | Spionida | Spionidae | Scolelepis | 4 |
| 131185 | *Spio martinensis* | Macrozoobenthos | Mesnil, 1896 | Animalia | Annelida | Polychaeta | Spionida | Spionidae | Spio | 10 |
| 131187 | *Spiophanes bombyx* | Macrozoobenthos | (Claparède, 1870) | Animalia | Annelida | Polychaeta | Spionida | Spionidae | Spiophanes | 3 |
| 131191 | *Streblospio benedicti* | Macrozoobenthos | Webster, 1879 | Animalia | Annelida | Polychaeta | Spionida | Spionidae | Streblospio | 3 |
| 131193 | *Streblospio shrubsolii* | Macrozoobenthos | (Buchanan, 1890) | Animalia | Annelida | Polychaeta | Spionida | Spionidae | Streblospio | 2 |
| 129775 | *Ampharete acutifrons* | Macrozoobenthos | (Grube, 1860) | Animalia | Annelida | Polychaeta | Terebellida | Ampharetidae | Ampharete | 3 |
| 129938 | *Aphelochaeta marioni* | Macrozoobenthos | (Saint-Joseph, 1894) | Animalia | Annelida | Polychaeta | Terebellida | Cirratulidae | Aphelochaeta | 4 |
| 152269 | *Tharyx killariensis* | Macrozoobenthos | (Southern, 1914) | Animalia | Annelida | Polychaeta | Terebellida | Cirratulidae | Tharyx | 13 |
| 152367 | *Lagis koreni* | Macrozoobenthos | Malmgren, 1866 | Animalia | Annelida | Polychaeta | Terebellida | Pectinariidae | Lagis | 2 |
| 131495 | *Lanice conchilega* | Macrozoobenthos; Zooplankton | (Pallas, 1766) | Animalia | Annelida | Polychaeta | Terebellida | Terebellidae | Lanice | 24 |
| 104496 | *Centropages hamatus* | Zooplankton | (Lilljeborg, 1853) | Animalia | Arthropoda | Copepoda | Calanoida | Centropagidae | Centropages | 5 |
| 104515 | *Pseudocalanus elongatus* | Zooplankton | (Brady, 1865) | Animalia | Arthropoda | Copepoda | Calanoida | Clausocalanidae | Pseudocalanus | 2 |
| 104685 | *Paracalanus parvus* | Zooplankton | (Claus, 1863) | Animalia | Arthropoda | Copepoda | Calanoida | Paracalanidae | Paracalanus | 4 |
| 360352 | *Pseudodiaptomus marinus* | Zooplankton | Sato, 1913 | Animalia | Arthropoda | Copepoda | Calanoida | Pseudodiaptomidae | Pseudodiaptomus | 1 |
| 104878 | *Temora longicornis* | Zooplankton | (Müller O.F., 1785) | Animalia | Arthropoda | Copepoda | Calanoida | Temoridae | Temora | 5 |
| 128805 | *Ditrichocorycaeus anglicus* | Zooplankton | (Lubbock, 1857) | Animalia | Arthropoda | Copepoda | Cyclopoida | Corycaeidae | Ditrichocorycaeus | 1 |
| 106651 | *Oithona nana* | Zooplankton | Giesbrecht, 1893 | Animalia | Arthropoda | Copepoda | Cyclopoida | Oithonidae | Oithona | 2 |
| 116162 | *Euterpina acutifrons* | Zooplankton | (Dana, 1847) | Animalia | Arthropoda | Copepoda | Harpacticoida | Tachidiidae | Euterpina | 5 |
| 103058 | *Bathyporeia elegans* | Macrozoobenthos | Watkin, 1938 | Animalia | Arthropoda | Malacostraca | Amphipoda | Bathyporeiidae | Bathyporeia | 1 |
| 103066 | *Bathyporeia pelagica* | Macrozoobenthos | (Spence Bate, 1857) | Animalia | Arthropoda | Malacostraca | Amphipoda | Bathyporeiidae | Bathyporeia | 2 |
| 103068 | *Bathyporeia pilosa* | Macrozoobenthos | Lindström, 1855 | Animalia | Arthropoda | Malacostraca | Amphipoda | Bathyporeiidae | Bathyporeia | 1 |
| 103073 | *Bathyporeia sarsi* | Macrozoobenthos | Watkin, 1938 | Animalia | Arthropoda | Malacostraca | Amphipoda | Bathyporeiidae | Bathyporeia | 4 |
| 102087 | *Corophium arenarium* | Macrozoobenthos | Crawford, 1937 | Animalia | Arthropoda | Malacostraca | Amphipoda | Corophiidae | Corophium | 9 |
| 102101 | *Corophium volutator* | Macrozoobenthos | (Pallas, 1766) | Animalia | Arthropoda | Malacostraca | Amphipoda | Corophiidae | Corophium | 22 |
| 102281 | *Gammarus locusta* | Macrozoobenthos | (Linnaeus, 1758) | Animalia | Arthropoda | Malacostraca | Amphipoda | Gammaridae | Gammarus | 3 |
| 102843 | *Melita palmata* | Macrozoobenthos | (Montagu, 1804) | Animalia | Arthropoda | Malacostraca | Amphipoda | Melitidae | Melita | 1 |
| 102916 | *Pontocrates altamarinus* | Macrozoobenthos | (Spence Bate & Westwood, 1862) | Animalia | Arthropoda | Malacostraca | Amphipoda | Oedicerotidae | Pontocrates | 2 |
| 103235 | *Urothoe poseidonis* | Macrozoobenthos | Reibish, 1905 | Animalia | Arthropoda | Malacostraca | Amphipoda | Urothoidae | Urothoe | 12 |
| 110445 | *Bodotria scorpioides* | Macrozoobenthos | (Montagu, 1804) | Animalia | Arthropoda | Malacostraca | Cumacea | Bodotriidae | Bodotria | 2 |
| 107381 | *Carcinus maenas* | Macrozoobenthos | (Linnaeus, 1758) | Animalia | Arthropoda | Malacostraca | Decapoda | Carcinidae | Carcinus | 41 |
| 107552 | *Crangon crangon* | Macrozoobenthos | (Linnaeus, 1758) | Animalia | Arthropoda | Malacostraca | Decapoda | Crangonidae | Crangon | 52 |
| 107007 | *Crangon sp.* | Macrozoobenthos | Fabricius, 1798 | Animalia | Arthropoda | Malacostraca | Decapoda | Crangonidae | Crangon | 13 |
| 106738 | Paguridae sp. | Macrozoobenthos | Latreille, 1802 | Animalia | Arthropoda | Malacostraca | Decapoda | Paguridae | not assigned | 8 |
| 107388 | *Liocarcinus holsatus* | Macrozoobenthos | (Fabricius, 1798) | Animalia | Arthropoda | Malacostraca | Decapoda | Polybiidae | Liocarcinus | 13 |
| 389288 | *Hemigrapsus takanoi* | Macrozoobenthos | Asakura & Watanabe, 2005 | Animalia | Arthropoda | Malacostraca | Decapoda | Varunidae | Hemigrapsus | 1 |
| 264171 | *Jaera albifrons* | Macrozoobenthos | Leach, 1814 | Animalia | Arthropoda | Malacostraca | Isopoda | Janiridae | Jaera | 1 |
| 120020 | *Gastrosaccus spinifer* | Macrozoobenthos | (Goës, 1864) | Animalia | Arthropoda | Malacostraca | Mysida | Mysidae | Gastrosaccus | 2 |
| 120072 | *Mesopodopsis slabberi* | Zooplankton | (Van Beneden, 1861) | Animalia | Arthropoda | Malacostraca | Mysida | Mysidae | Mesopodopsis | 6 |
| 120136 | *Neomysis integer* | Macrozoobenthos | (Leach, 1814) | Animalia | Arthropoda | Malacostraca | Mysida | Mysidae | Neomysis | 2 |
| 120203 | *Schistomysis kervillei* | Macrozoobenthos | (G.O. Sars, 1885) | Animalia | Arthropoda | Malacostraca | Mysida | Mysidae | Schistomysis | 4 |
| 106215 | *Balanus crenatus* | Macrozoobenthos | Bruguière, 1789 | Animalia | Arthropoda | Thecostraca | Balanomorpha | Balanidae | Balanus | 6 |
| 712167 | *Austrominius modestus* | Macrozoobenthos | (Darwin, 1854) | Animalia | Arthropoda | Thecostraca | Balanomorpha | Elminiidae | Austrominius | 2 |
| 103407 | *Oikopleura dioica* | Zooplankton | Fol, 1872 | Animalia | Chordata | Appendicularia | Copelata | Oikopleuridae | Oikopleura | 5 |
| 158939 | *Anas acuta* | Birds | Linnaeus, 1758 | Animalia | Chordata | Aves | Anseriformes | Anatidae | Anas | 3 |
| 158943 | *Anas crecca* | Birds | Linnaeus, 1758 | Animalia | Chordata | Aves | Anseriformes | Anatidae | Anas | 3 |
| 148791 | *Anas platyrhynchos* | Birds | Linnaeus, 1758 | Animalia | Chordata | Aves | Anseriformes | Anatidae | Anas | 3 |
| 159159 | *Anser albifrons* | Birds | (Scopoli, 1769) | Animalia | Chordata | Aves | Anseriformes | Anatidae | Anser | 1 |
| 416682 | *Anser anser* | Birds | (Linnaeus, 1758) | Animalia | Chordata | Aves | Anseriformes | Anatidae | Anser | 1 |
| 416683 | *Anser brachyrhynchus* | Birds | Baillon, 1834 | Animalia | Chordata | Aves | Anseriformes | Anatidae | Anser | 1 |
| 1488266 | *Anser serrirostris* | Birds | Gould, 1852 | Animalia | Chordata | Aves | Anseriformes | Anatidae | Anser | 1 |
| 159172 | *Aythya marila* | Birds | (Linnaeus, 1761) | Animalia | Chordata | Aves | Anseriformes | Anatidae | Aythya | 3 |
| 159175 | *Branta bernicla* | Birds | (Linnaeus, 1758) | Animalia | Chordata | Aves | Anseriformes | Anatidae | Branta | 3 |
| 159177 | *Branta leucopsis* | Birds | (Bechstein, 1803) | Animalia | Chordata | Aves | Anseriformes | Anatidae | Branta | 3 |
| 159197 | *Bucephala clangula* | Birds | (Linnaeus, 1758) | Animalia | Chordata | Aves | Anseriformes | Anatidae | Bucephala | 1 |
| 159088 | *Cygnus columbianus* | Birds | (Ord, 1815) | Animalia | Chordata | Aves | Anseriformes | Anatidae | Cygnus | 1 |
| 1464521 | *Mareca penelope* | Birds | (Linnaeus, 1758) | Animalia | Chordata | Aves | Anseriformes | Anatidae | Mareca | 3 |
| 159171 | *Mareca strepera* | Birds | (Linnaeus, 1758) | Animalia | Chordata | Aves | Anseriformes | Anatidae | Mareca | 1 |
| 137073 | *Melanitta nigra* | Birds | (Linnaeus, 1758) | Animalia | Chordata | Aves | Anseriformes | Anatidae | Melanitta | 2 |
| 232039 | *Mergellus albellus* | Birds | (Linnaeus, 1758) | Animalia | Chordata | Aves | Anseriformes | Anatidae | Mergellus | 1 |
| 159097 | *Mergus merganser* | Birds | Linnaeus, 1758 | Animalia | Chordata | Aves | Anseriformes | Anatidae | Mergus | 1 |
| 159098 | *Mergus serrator* | Birds | Linnaeus, 1758 | Animalia | Chordata | Aves | Anseriformes | Anatidae | Mergus | 1 |
| 137074 | *Somateria mollissima* | Birds | (Linnaeus, 1758) | Animalia | Chordata | Aves | Anseriformes | Anatidae | Somateria | 13 |
| 1461476 | *Spatula clypeata* | Birds | (Linnaeus, 1758) | Animalia | Chordata | Aves | Anseriformes | Anatidae | Spatula | 3 |
| 232042 | *Tadorna tadorna* | Birds | (Linnaeus, 1758) | Animalia | Chordata | Aves | Anseriformes | Anatidae | Tadorna | 32 |
| 212611 | *Charadrius alexandrinus* | Birds | Linnaeus, 1758 | Animalia | Chordata | Aves | Charadriiformes | Charadriidae | Charadrius | 17 |
| 212619 | *Charadrius hiaticula* | Birds | Linnaeus, 1758 | Animalia | Chordata | Aves | Charadriiformes | Charadriidae | Charadrius | 18 |
| 147436 | *Haematopus ostralegus* | Birds | Linnaeus, 1758 | Animalia | Chordata | Aves | Charadriiformes | Charadriidae | Haematopus | 39 |
| 159135 | *Pluvialis apricaria* | Birds | (Linnaeus, 1758) | Animalia | Chordata | Aves | Charadriiformes | Charadriidae | Pluvialis | 3 |
| 159137 | *Pluvialis squatarola* | Birds | (Linnaeus, 1758) | Animalia | Chordata | Aves | Charadriiformes | Charadriidae | Pluvialis | 3 |
| 159142 | *Vanellus vanellus* | Birds | (Linnaeus, 1758) | Animalia | Chordata | Aves | Charadriiformes | Charadriidae | Vanellus | 3 |
| 137137 | *Chlidonias niger* | Birds | (Linnaeus, 1758) | Animalia | Chordata | Aves | Charadriiformes | Laridae | Chlidonias | 1 |
| 148798 | *Gelochelidon nilotica* | Birds | (Gmelin, 1789) | Animalia | Chordata | Aves | Charadriiformes | Laridae | Gelochelidon | 1 |
| 137138 | *Larus argentatus* | Birds | Pontoppidan, 1763 | Animalia | Chordata | Aves | Charadriiformes | Laridae | Larus | 20 |
| 137141 | *Larus canus* | Birds | Linnaeus, 1758 | Animalia | Chordata | Aves | Charadriiformes | Laridae | Larus | 15 |
| 137142 | *Larus fuscus* | Birds | Linnaeus, 1758 | Animalia | Chordata | Aves | Charadriiformes | Laridae | Larus | 2 |
| 137146 | *Larus marinus* | Birds | Linnaeus, 1758 | Animalia | Chordata | Aves | Charadriiformes | Laridae | Larus | 3 |
| 137149 | *Larus ridibundus* | Birds | Linnaeus, 1766 | Animalia | Chordata | Aves | Charadriiformes | Laridae | Larus | 3 |
| 137162 | *Sterna hirundo* | Birds | Linnaeus, 1758 | Animalia | Chordata | Aves | Charadriiformes | Laridae | Sterna | 21 |
| 137165 | *Sterna paradisaea* | Birds | Pontoppidan, 1763 | Animalia | Chordata | Aves | Charadriiformes | Laridae | Sterna | 12 |
| 137166 | *Sterna sandvicensis* | Birds | Latham, 1787 | Animalia | Chordata | Aves | Charadriiformes | Laridae | Sterna | 2 |
| 567480 | *Sternula albifrons* | Birds | (Pallas, 1764) | Animalia | Chordata | Aves | Charadriiformes | Laridae | Sternula | 14 |
| 212721 | *Recurvirostra avosetta* | Birds | Linnaeus, 1758 | Animalia | Chordata | Aves | Charadriiformes | Recurvirostridae | Recurvirostra | 17 |
| 147431 | *Arenaria interpres* | Birds | (Linnaeus, 1758) | Animalia | Chordata | Aves | Charadriiformes | Scolopacidae | Arenaria | 3 |
| 159084 | *Calidris alba* | Birds | (Pallas, 1764) | Animalia | Chordata | Aves | Charadriiformes | Scolopacidae | Calidris | 3 |
| 159047 | *Calidris alpina* | Birds | (Linnaeus, 1758) | Animalia | Chordata | Aves | Charadriiformes | Scolopacidae | Calidris | 3 |
| 147433 | *Calidris canutus* | Birds | (Linnaeus, 1758) | Animalia | Chordata | Aves | Charadriiformes | Scolopacidae | Calidris | 3 |
| 159049 | *Calidris ferruginea* | Birds | (Pontoppidan, 1763) | Animalia | Chordata | Aves | Charadriiformes | Scolopacidae | Calidris | 3 |
| 159034 | *Limosa lapponica* | Birds | (Linnaeus, 1758) | Animalia | Chordata | Aves | Charadriiformes | Scolopacidae | Limosa | 3 |
| 159035 | *Limosa limosa* | Birds | (Linnaeus, 1758) | Animalia | Chordata | Aves | Charadriiformes | Scolopacidae | Limosa | 1 |
| 159037 | *Numenius arquata* | Birds | (Linnaeus, 1758) | Animalia | Chordata | Aves | Charadriiformes | Scolopacidae | Numenius | 3 |
| 159040 | *Numenius phaeopus* | Birds | (Linnaeus, 1758) | Animalia | Chordata | Aves | Charadriiformes | Scolopacidae | Numenius | 2 |
| 159046 | *Philomachus pugnax* | Birds | (Linnaeus, 1758) | Animalia | Chordata | Aves | Charadriiformes | Scolopacidae | Philomachus | 2 |
| 159029 | *Tringa erythropus* | Birds | (Pallas, 1764) | Animalia | Chordata | Aves | Charadriiformes | Scolopacidae | Tringa | 3 |
| 158968 | *Tringa nebularia* | Birds | (Gunnerus, 1767) | Animalia | Chordata | Aves | Charadriiformes | Scolopacidae | Tringa | 3 |
| 158970 | *Tringa totanus* | Birds | (Linnaeus, 1758) | Animalia | Chordata | Aves | Charadriiformes | Scolopacidae | Tringa | 34 |
| 212668 | *Ardea cinerea* | Birds | Linnaeus, 1758 | Animalia | Chordata | Aves | Ciconiiformes | Ardeidae | Ardea | 1 |
| 416678 | *Platalea leucorodia* | Birds | Linnaeus, 1758 | Animalia | Chordata | Aves | Ciconiiformes | Threskiornithidae | Platalea | 3 |
| 232054 | *Fulica atra* | Birds | Linnaeus, 1758 | Animalia | Chordata | Aves | Gruiformes | Rallidae | Fulica | 1 |
| 137179 | *Phalacrocorax carbo* | Birds | (Linnaeus, 1758) | Animalia | Chordata | Aves | Pelecaniformes | Phalacrocoracidae | Phalacrocorax | 3 |
| 137182 | *Podiceps cristatus* | Birds | (Linnaeus, 1758) | Animalia | Chordata | Aves | Podicipediformes | Podicipedidae | Podiceps | 1 |
| 126281 | *Anguilla anguilla* | Fish | (Linnaeus, 1758) | Animalia | Chordata | Teleostei | Anguilliformes | Anguillidae | Anguilla | 4 |
| 126375 | *Belone belone* | Fish | (Linnaeus, 1760) | Animalia | Chordata | Teleostei | Beloniformes | Belonidae | Belone | 2 |
| 126792 | *Callionymus lyra* | Fish | Linnaeus, 1758 | Animalia | Chordata | Teleostei | Callionymiformes | Callionymidae | Callionymus | 1 |
| 126822 | *Trachurus trachurus* | Fish | (Linnaeus, 1758) | Animalia | Chordata | Teleostei | Carangiformes | Carangidae | Trachurus | 3 |
| 126415 | *Alosa fallax* | Fish | (Lacepède, 1803) | Animalia | Chordata | Teleostei | Clupeiformes | Alosidae | Alosa | 3 |
| 126417 | *Clupea harengus* | Fish | Linnaeus, 1758 | Animalia | Chordata | Teleostei | Clupeiformes | Clupeidae | Clupea | 27 |
| 126425 | *Sprattus sprattus* | Fish | (Linnaeus, 1758) | Animalia | Chordata | Teleostei | Clupeiformes | Clupeidae | Sprattus | 18 |
| 126426 | *Engraulis encrasicolus* | Fish | (Linnaeus, 1758) | Animalia | Chordata | Teleostei | Clupeiformes | Engraulidae | Engraulis | 1 |
| 126436 | *Gadus morhua* | Fish | Linnaeus, 1758 | Animalia | Chordata | Teleostei | Gadiformes | Gadidae | Gadus | 10 |
| 126438 | *Merlangius merlangus* | Fish | (Linnaeus, 1758) | Animalia | Chordata | Teleostei | Gadiformes | Gadidae | Merlangius | 24 |
| 126445 | *Trisopterus luscus* | Fish | (Linnaeus, 1758) | Animalia | Chordata | Teleostei | Gadiformes | Gadidae | Trisopterus | 8 |
| 126448 | *Ciliata mustela* | Fish | (Linnaeus, 1758) | Animalia | Chordata | Teleostei | Gadiformes | Lotidae | Ciliata | 18 |
| 126868 | *Aphia minuta* | Fish | (Risso, 1810) | Animalia | Chordata | Teleostei | Gobiiformes | Gobiidae | Aphia | 1 |
| 126927 | *Pomatoschistus microps* | Fish | (Krøyer, 1838) | Animalia | Chordata | Teleostei | Gobiiformes | Gobiidae | Pomatoschistus | 1 |
| 126928 | *Pomatoschistus minutus* | Fish | (Pallas, 1770) | Animalia | Chordata | Teleostei | Gobiiformes | Gobiidae | Pomatoschistus | 3 |
| 125999 | *Pomatoschistus sp.* | Fish | Gill, 1863 | Animalia | Chordata | Teleostei | Gobiiformes | Gobiidae | Pomatoschistus | 22 |
| 126736 | *Osmerus eperlanus* | Fish | (Linnaeus, 1758) | Animalia | Chordata | Teleostei | Osmeriformes | Osmeridae | Osmerus | 26 |
| 127190 | *Agonus cataphractus* | Fish | (Linnaeus, 1758) | Animalia | Chordata | Teleostei | Perciformes | Agonidae | Agonus | 19 |
| 125909 | *Ammodytes sp.* | Fish | Linnaeus, 1758 | Animalia | Chordata | Teleostei | Perciformes | Ammodytidae | Ammodytes | 8 |
| 126752 | *Ammodytes tobianus* | Fish | Linnaeus, 1758 | Animalia | Chordata | Teleostei | Perciformes | Ammodytidae | Ammodytes | 2 |
| 126756 | *Hyperoplus lanceolatus* | Fish | (Le Sauvage, 1824) | Animalia | Chordata | Teleostei | Perciformes | Ammodytidae | Hyperoplus | 2 |
| 127203 | *Myoxocephalus scorpius* | Fish | (Linnaeus, 1758) | Animalia | Chordata | Teleostei | Perciformes | Cottidae | Myoxocephalus | 17 |
| 126505 | *Gasterosteus aculeatus* | Fish | Linnaeus, 1758 | Animalia | Chordata | Teleostei | Perciformes | Gasterosteidae | Gasterosteus | 2 |
| 127219 | *Liparis liparis* | Fish | (Linnaeus, 1766) | Animalia | Chordata | Teleostei | Perciformes | Liparidae | Liparis | 2 |
| 126160 | *Liparis sp.* | Fish | Scopoli, 1777 | Animalia | Chordata | Teleostei | Perciformes | Liparidae | Liparis | 5 |
| 126996 | *Pholis gunnellus* | Fish | (Linnaeus, 1758) | Animalia | Chordata | Teleostei | Perciformes | Pholidae | Pholis | 8 |
| 150630 | *Echiichthys vipera* | Fish | (Cuvier, 1829) | Animalia | Chordata | Teleostei | Perciformes | Trachinidae | Echiichthys | 1 |
| 127262 | *Chelidonichthys lucerna* | Fish | (Linnaeus, 1758) | Animalia | Chordata | Teleostei | Perciformes | Triglidae | Chelidonichthys | 4 |
| 127123 | *Zoarces viviparus* | Fish | (Linnaeus, 1758) | Animalia | Chordata | Teleostei | Perciformes | Zoarcidae | Zoarces | 17 |
| 101172 | *Lampetra fluviatilis* | Fish | (Linnaeus, 1758) | Animalia | Chordata | Teleostei | Petromyzontiformes | Petromyzontidae | Lampetra | 1 |
| 127139 | *Limanda limanda* | Fish | (Linnaeus, 1758) | Animalia | Chordata | Teleostei | Pleuronectiformes | Pleuronectidae | Limanda | 19 |
| 127140 | *Microstomus kitt* | Fish | (Walbaum, 1792) | Animalia | Chordata | Teleostei | Pleuronectiformes | Pleuronectidae | Microstomus | 1 |
| 127141 | *Platichthys flesus* | Fish | (Linnaeus, 1758) | Animalia | Chordata | Teleostei | Pleuronectiformes | Pleuronectidae | Platichthys | 22 |
| 127143 | *Pleuronectes platessa* | Fish | Linnaeus, 1758 | Animalia | Chordata | Teleostei | Pleuronectiformes | Pleuronectidae | Pleuronectes | 30 |
| 127149 | *Scophthalmus maximus* | Fish | (Linnaeus, 1758) | Animalia | Chordata | Teleostei | Pleuronectiformes | Scophthalmidae | Scophthalmus | 2 |
| 127150 | *Scophthalmus rhombus* | Fish | (Linnaeus, 1758) | Animalia | Chordata | Teleostei | Pleuronectiformes | Scophthalmidae | Scophthalmus | 3 |
| 127160 | *Solea solea* | Fish | (Linnaeus, 1758) | Animalia | Chordata | Teleostei | Pleuronectiformes | Soleidae | Solea | 19 |
| 127387 | *Syngnathus acus* | Fish | Linnaeus, 1758 | Animalia | Chordata | Teleostei | Syngnathiformes | Syngnathidae | Syngnathus | 1 |
| 127389 | *Syngnathus rostellatus* | Fish | Nilsson, 1855 | Animalia | Chordata | Teleostei | Syngnathiformes | Syngnathidae | Syngnathus | 19 |
| 126227 | *Syngnathus sp.* | Fish | Linnaeus, 1758 | Animalia | Chordata | Teleostei | Syngnathiformes | Syngnathidae | Syngnathus | 6 |
| 1360 | Actiniaria sp. | Macrozoobenthos | Hertwig, 1882 | Animalia | Cnidaria | Hexacorallia | Actiniaria | not assigned | not assigned | 2 |
| 123776 | *Asterias rubens* | Macrozoobenthos | Linnaeus, 1758 | Animalia | Echinodermata | Asteroidea | Forcipulatida | Asteriidae | Asterias | 7 |
| 124392 | *Echinocardium cordatum* | Macrozoobenthos | (Pennant, 1777) | Animalia | Echinodermata | Echinoidea | Spatangoida | Loveniidae | Echinocarium | 1 |
| 123574 | *Ophiura sp.* | Macrozoobenthos | Lamarck, 1801 | Animalia | Echinodermata | Ophiuroidae | Ophiurida | Ophiuridae | Ophiura | 3 |
| 876640 | *Ensis leei* | Macrozoobenthos | Huber, 2015 | Animalia | Mollusca | Bivalvia | Adapedonta | Pharidae | Ensis | 12 |
| 138998 | *Cerastoderma edule* | Macrozoobenthos | (Linnaeus, 1758) | Animalia | Mollusca | Bivalvia | Cardiida | Cardiidae | Cerastoderma | 30 |
| 141433 | *Abra alba* | Macrozoobenthos | (W. Wood, 1802) | Animalia | Mollusca | Bivalvia | Cardiida | Semelidae | Abra | 1 |
| 141439 | *Abra tenuis* | Macrozoobenthos | (Montagu, 1803) | Animalia | Mollusca | Bivalvia | Cardiida | Semelidae | Abra | 2 |
| 141424 | *Scrobicularia plana* | Macrozoobenthos | (da Costa, 1778) | Animalia | Mollusca | Bivalvia | Cardiida | Semelidae | Scrobicularia | 13 |
| 146907 | *Fabulina fabula* | Macrozoobenthos | (Gmelin, 1791) | Animalia | Mollusca | Bivalvia | Cardiida | Tellinidae | Fabulina | 2 |
| 141579 | *Macoma balthica* | Macrozoobenthos | (Linnaeus, 1758) | Animalia | Mollusca | Bivalvia | Cardiida | Tellinidae | Macoma | 58 |
| 878470 | *Macomangulus tenuis* | Macrozoobenthos | (da Costa, 1778) | Animalia | Mollusca | Bivalvia | Cardiida | Tellinidae | Macomangulus | 3 |
| 345281 | *Kurtiella bidentata* | Macrozoobenthos | (Montagu, 1803) | Animalia | Mollusca | Bivalvia | Galeommatida | Lasaeidae | Kurtiella | 3 |
| 146952 | *Tellimya ferruginosa* | Macrozoobenthos | (Montagu, 1808) | Animalia | Mollusca | Bivalvia | Galeommatida | Lasaeidae | Tellimya | 1 |
| 140430 | *Mya arenaria* | Macrozoobenthos | Linnaeus, 1758 | Animalia | Mollusca | Bivalvia | Myida | Myidae | Mya | 31 |
| 140480 | *Mytilus edulis* | Macrozoobenthos | Linneaus, 1758 | Animalia | Mollusca | Bivalvia | Mytilida | Mytilidae | Mytilus | 12 |
| 836033 | *Magallana gigas* | Macrozoobenthos | (Thunberg, 1793) | Animalia | Mollusca | Bivalvia | Ostreida | Ostreidae | Magallana | 1 |
| 139604 | *Donax vittatus* | Macrozoobenthos | (da Costa, 1778) | Animalia | Mollusca | Bivalvia | Veneroida | Donacidae | Donax | 1 |
| 141134 | *Retusa obtusa* | Macrozoobenthos | (Montagu, 1803 | Animalia | Mollusca | Gastropoda | Cephalaspidea | Retusidae | Retusa | 4 |
| 138963 | *Crepidula fornicata* | Macrozoobenthos | (Linnaeus, 1758) | Animalia | Mollusca | Gastropoda | Littorinimorpha | Calyptraeidae | Crepidula | 1 |
| 151628 | *Peringia ulvae* | Macrozoobenthos | (Pennant, 1777) | Animalia | Mollusca | Gastropoda | Littorinimorpha | Hydrobiidae | Peringia | 27 |
| 140262 | *Littorina littorea* | Macrozoobenthos | (Linnaeus, 1758) | Animalia | Mollusca | Gastropoda | Littorinimorpha | Littorinidae | Littorina | 4 |
| 152774 | *Lepidochitona cinerea* | Macrozoobenthos | (Linnaeus, 1767) | Animalia | Mollusca | Polyplacophora | Chitonida | Tonicellidae | Lepidochitona | 1 |
| 152391 | Nemertea sp. | Macrozoobenthos | Schultze, 1851 | Animalia | Nemertea | not assigned | not assigned | not assigned | not assigned | 7 |
| 128545 | *Phoronis sp.* | Macrozoobenthos | Wright, 1856 | Animalia | Phoronida | not assigned | not assigned | Phoronidae | Phoronis | 1 |
| 146543 | Chroococcales sp. | Phytoplankton | von Wettstein von Westerheim, 2002 | Bacteria | Cyanobacteria | Cyanophyceae | Chroococcales | not assigned | not assigned | 2 |
| 146552 | *Planktothrix agardhii* | Phytoplankton | (Gomont) Anagnostidis & Komárek, 1988 | Bacteria | Cyanobacteria | Cyanophyceae | Oscillatoriales | Microcoleaceae | Planktothrix | 1 |
| 118047 | *Cryothecomonas scybalophora* | Phytoplankton | Thomsen, Buck, Bolt & Garriso, 1991 | Chromista | Cercozoa | Thecofilosea | Cryomonadida | Protaspidae | Cryothecomonas | 2 |
| 118051 | *Ebria tripartita* | Phytoplankton | (Schumann) Lemmermann, 1899 | Chromista | Cercozoa | Thecofilosea | Ebriales | Ebriaceae | Ebria | 8 |
| 232069 | *Mesodinium rubrum* | Zooplankton | (Lohmann, 1908) | Chromista | Ciliophora | Litostomatea | Cyclotrichiida | Mesodiniidae | Mesodinium | 8 |
| 101264 | *Laboea strobila* | Phytoplankton | Lohmann, 1908 | Chromista | Ciliophora | Oligotrichea | Oligotrichida | Tontoniidae | Laboea | 8 |
| 627117 | *Katablepharis remigera* | Phytoplankton | (N.Vørs) Clay & Kugrens, 2000 | Chromista | Cryptista | Katablepharidophyceae | Katablepharidales | Katablepharidaceae | Katablepharis | 8 |
| 119077 | *Leucocryptos marina* | Phytoplankton | (Braarud) Butcher, 1967 | Chromista | Cryptista | Katablepharidophyceae | Katablepharidales | Katablepharidaceae | Leucocryptos | 8 |
| 17640 | Cryptomonadales sp. | Phytoplankton | Pringsheim, 1944 | Chromista | Cryptophyta | Cryptophyceae | Cryptomonadales | not assigned | not assigned | 8 |
| 17639 | Cryptophyceae sp. | Phytoplankton | Fritsch, 1927 | Chromista | Cryptophyta | Cryptophyceae | not assigned | not assigned | not assigned | 4 |
| 106310 | *Hemiselmis virescens* | Phytoplankton | Droop, 1955 | Chromista | Cryptophyta | Cryptophyceae | Pyrenomonadales | Chroomonadacea | Hemiselmis | 8 |
| 106303 | *Plagioselmis prolonga* | Phytoplankton | Butcher ex Novarino, Lucas & Morrall, 1994 | Chromista | Cryptophyta | Cryptophyceae | Pyrenomonadales | Geminigeraceae | Plagioselmis | 8 |
| 106305 | *Teleaulax acuta* | Phytoplankton | (Butcher) Hill, 1991 | Chromista | Cryptophyta | Cryptophyceae | Pyrenomonadales | Geminigeraceae | Teleaulax | 8 |
| 106306 | *Teleaulax amphioxeia* | Phytoplankton | (W.Conrad) Hill, 1992 | Chromista | Cryptophyta | Cryptophyceae | Pyrenomonadales | Geminigeraceae | Teleaulax | 8 |
| 106281 | *Chroomonas sp.* | Phytoplankton | Hansgirg, 1885 | Chromista | Cryptophyta | Cryptophyceae | Pyrenomonadales | Hemiselmidaceae | Chroomonas | 7 |
| 106295 | *Chroomonas vectensis* | Phytoplankton | Carter, 1937 | Chromista | Cryptophyta | Cryptophyceae | Pyrenomonadales | Hemiselmidaceae | Chroomonas | 2 |
| 106314 | *Rhodomonas marina* | Phytoplankton | (P.A.Dangeard) Lemmermann, 1899 | Chromista | Cryptophyta | Cryptophyceae | Pyrenomonadales | Pyrenomonadaceae | Rhodomonas | 8 |
| 118028 | *Telonema sp.* | Phytoplankton | Griessmann, 1913 | Chromista | Cryptophyta | Telonemea | Telonemida | Telonemidae | Telonema | 8 |
| 109726 | *Amphidinium crassum* | Phytoplankton | Lohmann, 1908 | Chromista | Dinoflagellata | Dinophyceae | Amphidiniales | Amphidiniaceae | Amphidinium | 1 |
| 156505 | *Phalacroma rotundatum* | Phytoplankton | (Claparéde & Lachmann) Kofoid & Michener, 1911 | Chromista | Dinoflagellata | Dinophyceae | Dinophysales | Oxyphysaceae | Phalacroma | 1 |
| 840626 | *Tripos fusus* | Phytoplankton | (Ehrenberg) Gómez, 2013 | Chromista | Dinoflagellata | Dinophyceae | Gonyaulacales | Ceratiaceae | Tripos | 4 |
| 109519 | *Gonyaulax sp.* | Phytoplankton | Diesing, 1866 | Chromista | Dinoflagellata | Dinophyceae | Gonyaulacales | Gonyaulacaceae | Gonyaulax | 1 |
| 109826 | *Gymnodinium rhomboides* | Phytoplankton | Schütt, 1895 | Chromista | Dinoflagellata | Dinophyceae | Gymnodiniales | Gymnodiniaceae | Gymnodinium | 3 |
| 109475 | *Gymnodinium sp.* | Phytoplankton | Stein, 1878 | Chromista | Dinoflagellata | Dinophyceae | Gymnodiniales | Gymnodiniaceae | Gymnodinium | 8 |
| 109854 | *Gyrodinium flagellare* | Phytoplankton | Schiller, 1928 | Chromista | Dinoflagellata | Dinophyceae | Gymnodiniales | Gymnodiniaceae | Gyrodinium | 6 |
| 109476 | *Gyrodinium sp.* | Phytoplankton | Kofoid & Swezy, 1921 | Chromista | Dinoflagellata | Dinophyceae | Gymnodiniales | Gymnodiniaceae | Gyrodinium | 12 |
| 109876 | *Gyrodinium spirale* | Phytoplankton | (Bergh) Kofoid & Swezy, 1921 | Chromista | Dinoflagellata | Dinophyceae | Gymnodiniales | Gymnodiniaceae | Gyrodinium | 6 |
| 345481 | *Lepidodinium chlorophorum* | Phytoplankton | (Elbrächter & Schnepf) Hansen, Botes & Salas, 2007 | Chromista | Dinoflagellata | Dinophyceae | Gymnodiniales | Gymnodiniaceae | Lepidodinium | 4 |
| 1382182 | *Lebouridinium glaucum* | Phytoplankton | (Lebour) Gómez, Takayam, Moreira & López-García, 2016 | Chromista | Dinoflagellata | Dinophyceae | Gymnodiniales | not assigned | Lebouridinium | 10 |
| 109901 | *Polykrikos schwartzii* | Phytoplankton | Bütschli, 1873 | Chromista | Dinoflagellata | Dinophyceae | Gymnodiniales | Polykrikaceae | Polykrikos | 2 |
| 547527 | *Nematopsides vigilans* | Phytoplankton | (Marshall) Greuet, 1973 | Chromista | Dinoflagellata | Dinophyceae | Gymnodiniales | Warnowiaceae | Nematopsides | 8 |
| 109921 | *Noctiluca scintillans* | Phytoplankton | (Macartney) Kofoid & Swezy, 1921 | Chromista | Dinoflagellata | Dinophyceae | Noctilucales | Noctilucaceae | Noctiluca | 8 |
| 110152 | *Heterocapsa rotundata* | Phytoplankton | (Lohmann) Hansen, 1995 | Chromista | Dinoflagellata | Dinophyceae | Peridiniales | Heterocapsaceae | Heterocapsa | 7 |
| 109540 | *Heterocapsa sp.* | Phytoplankton | Stein, 1883 | Chromista | Dinoflagellata | Dinophyceae | Peridiniales | Heterocapsaceae | Heterocapsa | 8 |
| 1346683 | *Kryptoperidinium triquetrum* | Phytoplankton | (Ehrenberg) Tillmann, Gottschling, Elbrächter, Kusber & Hoppenrath, 2019 | Chromista | Dinoflagellata | Dinophyceae | Peridiniales | Kryptoperidiniaceae | Kryptoperidinium | 5 |
| 109394 | Peridiniales sp. | Phytoplankton | Haeckel, 1894 | Chromista | Dinoflagellata | Dinophyceae | Peridiniales | not assigned | not assigned | 8 |
| 233369 | *Peridiniella danica* | Phytoplankton | (Paulsen) Okolodkov & Dodge, 1995 | Chromista | Dinoflagellata | Dinophyceae | Peridiniales | not assigned | Peridiniella | 7 |
| 624607 | *Archaeperidinium minutum* | Phytoplankton | (Kofoid) Jørgensen, 1912 | Chromista | Dinoflagellata | Dinophyceae | Peridiniales | Protoperidiniaceae | Archaeperidinium | 2 |
| 604918 | *Archaeperidinium sp.* | Phytoplankton | Jörgensen, 1912 | Chromista | Dinoflagellata | Dinophyceae | Peridiniales | Protoperidiniaceae | Archaeperidinium | 4 |
| 109515 | *Diplopsalis sp.* | Phytoplankton | Bergh, 1881 | Chromista | Dinoflagellata | Dinophyceae | Peridiniales | Protoperidiniaceae | Diplopsalis | 8 |
| 614618 | *Preperidinium meunieri* | Phytoplankton | (Pavillard) Elbrächter, 1993 | Chromista | Dinoflagellata | Dinophyceae | Peridiniales | Protoperidiniaceae | Preperidinium | 8 |
| 110205 | *Protoperidinium achromaticum* | Phytoplankton | (Levander) Balech, 1974 | Chromista | Dinoflagellata | Dinophyceae | Peridiniales | Protoperidiniaceae | Protoperidinium | 5 |
| 110208 | *Protoperidinium bipes* | Phytoplankton | (Paulsen, 1904) Balech, 1974 | Chromista | Dinoflagellata | Dinophyceae | Peridiniales | Protoperidiniaceae | Protoperidinium | 13 |
| 110210 | *Protoperidinium brevipes* | Phytoplankton | (Paulsen, 1908) Balech, 1974 | Chromista | Dinoflagellata | Dinophyceae | Peridiniales | Protoperidiniaceae | Protoperidinium | 5 |
| 110213 | *Protoperidinium conicum* | Phytoplankton | (Gran) Balech, 1974 | Chromista | Dinoflagellata | Dinophyceae | Peridiniales | Protoperidiniaceae | Protoperidinium | 6 |
| 109553 | *Protoperidinium sp.* | Phytoplankton | Bergh, 1881 | Chromista | Dinoflagellata | Dinophyceae | Peridiniales | Protoperidiniaceae | Protoperidinium | 6 |
| 110257 | *Protoperidinium steinii* | Phytoplankton | (Jørgensen, 1899) Balech, 1974 | Chromista | Dinoflagellata | Dinophyceae | Peridiniales | Protoperidiniaceae | Protoperidinium | 2 |
| 232376 | *Prorocentrum cordatum* | Phytoplankton | (Ostenfeld) Dodge, 1976 | Chromista | Dinoflagellata | Dinophyceae | Prorocentrales | Prorocentraceae | Prorocentrum | 4 |
| 110303 | *Prorocentrum micans* | Phytoplankton | Ehrenberg, 1834 | Chromista | Dinoflagellata | Dinophyceae | Prorocentrales | Prorocentraceae | Prorocentrum | 8 |
| 110316 | *Prorocentrum triestinum* | Phytoplankton | Schiller, 1918 | Chromista | Dinoflagellata | Dinophyceae | Prorocentrales | Prorocentraceae | Prorocentrum | 13 |
| 109545 | *Scrippsiella sp.* | Phytoplankton | Balech ex Loeblich III, 1965 | Chromista | Dinoflagellata | Dinophyceae | Thoracosphaerales | Thoracosphaeraceae | Scrippsiella | 8 |
| 109889 | *Torodinium robustum* | Phytoplankton | Kofoid & Swezy, 1921 | Chromista | Dinoflagellata | Dinophyceae | Torodiniales | Torodiniaceae | Torodinium | 4 |
| 160538 | *Phaeocystis globosa* | Phytoplankton | Scherffel, 1899 | Chromista | Haptophyta | Coccolithophyceae | Phaeocystales | Phaeocystaceae | Phaeocystis | 8 |
| 115088 | *Phaeocystis sp.* | Phytoplankton | Lagerheim, 1893 | Chromista | Haptophyta | Coccolithophyceae | Phaeocystales | Phaeocystaceae | Phaeocystis | 8 |
| 149004 | *Cylindrotheca closterium* | Phytoplankton | (Ehrenberg) Reimann & Lewin, 1964 | Chromista | Heterokontophyta | Bacillariophyceae | Bacillariales | Bacillariaceae | Cylindrotheca | 10 |
| 149213 | *Nitzschia sigma* | Phytoplankton | (Kützing) Smith, 1853 | Chromista | Heterokontophyta | Bacillariophyceae | Bacillariales | Bacillariaceae | Nitzschia | 1 |
| 149045 | *Nitzschia sp.* | Phytoplankton | Hassall, 1845 | Chromista | Heterokontophyta | Bacillariophyceae | Bacillariales | Bacillariaceae | Nitzschia | 1 |
| 149153 | *Pseudo-nitzschia delicatissima* | Phytoplankton | (Cleve) Heiden, 1928 | Chromista | Heterokontophyta | Bacillariophyceae | Bacillariales | Bacillariaceae | Pseudo-nitzschia | 2 |
| 246606 | *Pseudo-nitzschia fraudulenta* | Phytoplankton | (Cleve) Hasle, 1993 | Chromista | Heterokontophyta | Bacillariophyceae | Bacillariales | Bacillariaceae | Pseudo-nitzschia | 6 |
| 160528 | *Pseudo-nitzschia pungens* | Phytoplankton | (Grunow ex Cleve) Hasle, 1993 | Chromista | Heterokontophyta | Bacillariophyceae | Bacillariales | Bacillariaceae | Pseudo-nitzschia | 2 |
| 149151 | *Pseudo-nitzschia sp.* | Phytoplankton | Peragallo in Peragallo & Peragallo, 1900 | Chromista | Heterokontophyta | Bacillariophyceae | Bacillariales | Bacillariaceae | Pseudo-nitzschia | 16 |
| 149001 | Bacillariales sp. | Phytoplankton | Hendey, 1937 sensu emend. | Chromista | Heterokontophyta | Bacillariophyceae | Bacillariales | not assigned | not assigned | 4 |
| 149018 | *Diploneis sp.* | Phytoplankton | (Ehrenberg) Cleve, 1894 | Chromista | Heterokontophyta | Bacillariophyceae | Naviculales | Diploneidaceae | Diploneis | 1 |
| 149494 | *Gyrosigma fasciola* | Phytoplankton | (Ehrenberg) Griffith & Henfrey, 1856 | Chromista | Heterokontophyta | Bacillariophyceae | Naviculales | Naviculaceae | Gyrosigma | 2 |
| 149033 | *Gyrosigma sp.* | Phytoplankton | Hassall, 1845 | Chromista | Heterokontophyta | Bacillariophyceae | Naviculales | Naviculaceae | Gyrosigma | 8 |
| 149320 | *Navicula transitans* | Phytoplankton | Cleve, 1883 | Chromista | Heterokontophyta | Bacillariophyceae | Naviculales | Naviculaceae | Navicula | 6 |
| 149181 | *Pleurosigma sp.* | Phytoplankton | Smith, 1852 | Chromista | Heterokontophyta | Bacillariophyceae | Naviculales | Pleurosigmataceae | Pleurosigma | 2 |
| 149139 | *Asterionellopsis glacialis* | Phytoplankton | (Castracane) Round, 1990 | Chromista | Heterokontophyta | Bacillariophyceae | Rhaphoneidales | Asterionellopsidaceae | Asterionellopsis | 18 |
| 251745 | *Asteroplanus karianus* | Phytoplankton | (Grunow) Gardner & Crawford, 1997 | Chromista | Heterokontophyta | Bacillariophyceae | Rhaphoneidales | Asterionellopsidaceae | Asteroplanus | 14 |
| 149179 | *Delphineis sp.* | Phytoplankton | Andrews, 1977 | Chromista | Heterokontophyta | Bacillariophyceae | Rhaponeidales | Rhaphoneidaceae | Delphineis | 8 |
| 149066 | *Rhaphoneis amphiceros* | Phytoplankton | (Ehrenberg) Ehrenberg, 1844 | Chromista | Heterokontophyta | Bacillariophyceae | Rhaponeidales | Rhaphoneidaceae | Rhaphoneis | 3 |
| 156598 | *Entomoneis sp.* | Phytoplankton | Ehrenberg, 1845 | Chromista | Heterokontophyta | Bacillariophyceae | Surirellales | Entomoneidaceae | Entomoneis | 1 |
| 149084 | *Surirella sp.* | Phytoplankton | Turpin, 1828 | Chromista | Heterokontophyta | Bacillariophyceae | Surirellales | Surirellaceae | Surirella | 2 |
| 149093 | *Thalassionema nitzschioides* | Phytoplankton | (Grunow) Mereschkowsky, 1902 | Chromista | Heterokontophyta | Bacillariophyceae | Thalassionematales | Thalassionemataceae | Thalassionema | 19 |
| 345485 | *Mediopyxis helysia* | Phytoplankton | Kühn, Hargreaves & Halliger, 2006 | Chromista | Heterokontophyta | Bacillariophyceae | not assigned | not assigned | Mediopyxis | 11 |
| 1322474 | *Ralfsiella minima* | Phytoplankton | (Grunow in Van Heurck) Sims & Williams in Sims et al., 2018 | Chromista | Heterokontophyta | Bacillariophyceae | Triceratiales | Triceratiaceae | Ralfsiella | 8 |
| 160552 | *Dinobryon balticum* | Phytoplankton | (Schütt) Lemmermann, 1901 | Chromista | Heterokontophyta | Chrysophyceae | Chromulinales | Dinobryaceae | Dinobryon | 3 |
| 160553 | *Dinobryon faculiferum* | Phytoplankton | (Willén) Willén, 1992 | Chromista | Heterokontophyta | Chrysophyceae | Chromulinales | Dinobryaceae | Dinobryon | 2 |
| 148992 | *Coscinodiscus concinnus* | Phytoplankton | Smith, 1856 | Chromista | Heterokontophyta | Coscinodiscophyceae | Coscinodiscales | Coscinodiscaceae | Coscinodiscus | 3 |
| 149158 | *Coscinodiscus radiatus* | Phytoplankton | Ehrenberg, 1840 | Chromista | Heterokontophyta | Coscinodiscophyceae | Coscinodiscales | Coscinodiscaceae | Coscinodiscus | 2 |
| 148948 | *Actinoptychus senarius* | Phytoplankton | (Ehrenberg) Ehrenberg, 1843 | Chromista | Heterokontophyta | Coscinodiscophyceae | Coscinodiscales | Heliopeltaceae | Actinoptychus | 19 |
| 148949 | *Actinoptychus splendens* | Phytoplankton | (Shadbolt) Ralfs, 1861 | Chromista | Heterokontophyta | Coscinodiscophyceae | Coscinodiscales | Heliopeltaceae | Actinoptychus | 3 |
| 149163 | *Actinocyclus octonarius* | Phytoplankton | Ehrenberg, 1837 | Chromista | Heterokontophyta | Coscinodiscophyceae | Coscinodiscales | Hemidiscaceae | Actinocyclus | 7 |
| 149060 | *Podosira stelligera* | Phytoplankton | (Bailey) Mann, 1907 | Chromista | Heterokontophyta | Coscinodiscophyceae | Melosirales | Hyalodiscaceae | Podosira | 2 |
| 149044 | *Melosira nummuloides* | Phytoplankton | Agardh, 1824 | Chromista | Heterokontophyta | Coscinodiscophyceae | Melosirales | Melosiraceae | Melosira | 2 |
| 149055 | *Paralia sulcata* | Phytoplankton | (Ehrenberg) Cleve, 1873 | Chromista | Heterokontophyta | Coscinodiscophyceae | Paraliales | Paraliaceae | Paralia | 12 |
| 149310 | *Dactyliosolen fragilissimus* | Phytoplankton | (Bergon) Hasle, 1996 | Chromista | Heterokontophyta | Coscinodiscophyceae | Rhizosoleniales | Rhizosoleniaceae | Dactyliosolen | 6 |
| 149112 | *Guinardia delicatula* | Phytoplankton | (Cleve) Hasle, 1997 | Chromista | Heterokontophyta | Coscinodiscophyceae | Rhizosoleniales | Rhizosoleniaceae | Guinardia | 19 |
| 149132 | *Guinardia flaccida* | Phytoplankton | (Castracane) Peragallo, 1892 | Chromista | Heterokontophyta | Coscinodiscophyceae | Rhizosoleniales | Rhizosoleniaceae | Guinardia | 13 |
| 149113 | *Guinardia striata* | Phytoplankton | (Stolterfoth) Hasle, 1996 | Chromista | Heterokontophyta | Coscinodiscophyceae | Rhizosoleniales | Rhizosoleniaceae | Guinardia | 2 |
| 149116 | *Rhizosolenia imbricata* | Phytoplankton | Brightwell, 1858 | Chromista | Heterokontophyta | Coscinodiscophyceae | Rhizosoleniales | Rhizosoleniaceae | Rhizosolenia | 19 |
| 1590706 | *Sundstroemia setigera* | Phytoplankton | (Brightwell) Medlin in Medlin et al., 2021 | Chromista | Heterokontophyta | Coscinodiscophyceae | Rhizosoleniales | Rhizosoleniaceae | Sundstroemia | 20 |
| 1590709 | *Sundstroemia similoides* | Phytoplankton | (Cl.-Euler) Medlin, Lundholm, Boonprakob & Moestrup in Medlin et al., 2021 | Chromista | Heterokontophyta | Coscinodiscophyceae | Rhizosoleniales | Rhizosoleniaceae | Sundstroemia | 8 |
| 1310442 | *Octactis speculum* | Phytoplankton | (Ehrenberg) Chang, Grieve & Sutherland, 2017 | Chromista | Heterokontophyta | Dictyochophyceae | Dictyochales | Dictyochaceae | Octactis | 2 |
| 160600 | *Pseudopedinella pyriformis* | Phytoplankton | Carter, 1937 | Chromista | Heterokontophyta | Dictyochophyceae | Pedinellales | Actinomonadaceae | Pseudopedinella | 8 |
| 160599 | *Pseudopedinella sp.* | Phytoplankton | Carter, 1937 | Chromista | Heterokontophyta | Dictyochophyceae | Pedinellales | Actinomonadaceae | Pseudopedinella | 8 |
| 345464 | *Eunotogramma dubium* | Phytoplankton | Hustedt, 1939 | Chromista | Heterokontophyta | Mediophyceae | Anaulales | Anaulaceae | Eunotogramma | 18 |
| 149655 | *Neobrightwellia alternans* | Phytoplankton | (Bailey) Ashworth & Sims, 2023 | Chromista | Heterokontophyta | Mediophyceae | Biddulphiales | Biddulphiaceae | Neobrightwellia | 13 |
| 149119 | *Bacteriastrum hyalinum* | Phytoplankton | Lauder, 1864 | Chromista | Heterokontophyta | Mediophyceae | Chaetocerotales | Chaetocerotaceae | Bacteriastrum | 6 |
| 149623 | *Chaetoceros contortus* | Phytoplankton | Schütt, 1895 | Chromista | Heterokontophyta | Mediophyceae | Chaetocerotales | Chaetocerotaceae | Chaetoceros | 3 |
| 149120 | *Chaetoceros danicus* | Phytoplankton | Cleve, 1889 | Chromista | Heterokontophyta | Mediophyceae | Chaetocerotales | Chaetocerotaceae | Chaetoceros | 5 |
| 149219 | *Chaetoceros debilis* | Phytoplankton | Cleve, 1894 emend Xu, Li & Lundholm in Xu et al., 2020 | Chromista | Heterokontophyta | Mediophyceae | Chaetocerotales | Chaetocerotaceae | Chaetoceros | 11 |
| 149121 | *Chaetoceros densus* | Phytoplankton | (Cleve) Cleve, 1899 | Chromista | Heterokontophyta | Mediophyceae | Chaetocerotales | Chaetocerotaceae | Chaetoceros | 10 |
| 149128 | *Chaetoceros diadema* | Phytoplankton | (Ehrenberg) Gran, 1897 | Chromista | Heterokontophyta | Mediophyceae | Chaetocerotales | Chaetocerotaceae | Chaetoceros | 2 |
| 149122 | *Chaetoceros didymus* | Phytoplankton | Ehrenberg, 1845 | Chromista | Heterokontophyta | Mediophyceae | Chaetocerotales | Chaetocerotaceae | Chaetoceros | 1 |
| 160521 | *Chaetoceros eibenii* | Phytoplankton | Grunow, 1882 | Chromista | Heterokontophyta | Mediophyceae | Chaetocerotales | Chaetocerotaceae | Chaetoceros | 1 |
| 160523 | *Chaetoceros lauderi* | Phytoplankton | Ralfs ex Lauder, 1864 | Chromista | Heterokontophyta | Mediophyceae | Chaetocerotales | Chaetocerotaceae | Chaetoceros | 5 |
| 178207 | *Chaetoceros neogracilis* | Phytoplankton | VanLandingham, 1968 | Chromista | Heterokontophyta | Mediophyceae | Chaetocerotales | Chaetocerotaceae | Chaetoceros | 1 |
| 149123 | *Chaetoceros socialis* | Phytoplankton | Lauder, 1864 | Chromista | Heterokontophyta | Mediophyceae | Chaetocerotales | Chaetocerotaceae | Chaetoceros | 12 |
| 148985 | *Chaetoceros sp.* | Phytoplankton | Ehrenberg, 1844 | Chromista | Heterokontophyta | Mediophyceae | Chaetocerotales | Chaetocerotaceae | Chaetoceros | 12 |
| 156621 | *Chaetoceros subtilis* | Phytoplankton | Cleve, 1896 | Chromista | Heterokontophyta | Mediophyceae | Chaetocerotales | Chaetocerotaceae | Chaetoceros | 2 |
| 148984 | Chaetocerotaceae | Phytoplankton | Ralfs, 1861 | Chromista | Heterokontophyta | Mediophyceae | Chaetocerotales | Chaetocerotaceae | not assigned | 1 |
| 149106 | *Leptocylindrus danicus* | Phytoplankton | Cleve, 1889 | Chromista | Heterokontophyta | Mediophyceae | Chaetocerotales | Leptocylindraceae | Leptocylindrus | 12 |
| 149039 | *Leptocylindrus minimus* | Phytoplankton | Gran, 1915 | Chromista | Heterokontophyta | Mediophyceae | Chaetocerotales | Leptocylindraceae | Leptocylindrus | 12 |
| 149038 | *Leptocylindrus sp.* | Phytoplankton | Cleve in Petersen, 1889 | Chromista | Heterokontophyta | Mediophyceae | Chaetocerotales | Leptocylindraceae | Leptocylindrus | 6 |
| 149037 | Leptocylindraceae sp. | Phytoplankton | Lebour, 1930 | Chromista | Heterokontophyta | Mediophyceae | Chaetocerotales | Leptocylindraceae | not assigned | 8 |
| 149137 | *Brockmanniella brockmannii* | Phytoplankton | (Hustedt) Hasle, Stosch & Syvertsen, 1983 | Chromista | Heterokontophyta | Mediophyceae | Cymatosirales | Cymatosiraceae | Brockmanniella | 12 |
| 549205 | *Lennoxia faveolata* | Phytoplankton | Thomsen & Buck, 1993 | Chromista | Heterokontophyta | Mediophyceae | Cymatosirales | Cymatosiraceae | Lennoxia | 1 |
| 573486 | *Minutocellus scriptus* | Phytoplankton | Hasle, von Stosch & Syvertsen, 1983 | Chromista | Heterokontophyta | Mediophyceae | Cymatosirales | Cymatosiraceae | Minutocellus | 5 |
| 149056 | *Plagiogrammopsis sp.* | Phytoplankton | Hasle, von Stosch & Syvertsem, 1983 | Chromista | Heterokontophyta | Mediophyceae | Cymatosirales | Cymatosiraceae | Plagiogrammopsis | 8 |
| 149057 | *Plagiogrammopsis vanheurckii* | Phytoplankton | (Grunow) Hasle, von Stosch & Syvertsen, 1983 | Chromista | Heterokontophyta | Mediophyceae | Cymatosirales | Cymatosiraceae | Plagiogrammopsis | 12 |
| 1322477 | *Cerataulus granulatus* | Phytoplankton | (Roper) Sims & Williams, 2018 | Chromista | Heterokontophyta | Mediophyceae | Eupodiscales | Eupodiscaceae | Cerataulus | 7 |
| 149157 | *Zygoceros ehrenbergii* | Phytoplankton | Sar, 2016 | Chromista | Heterokontophyta | Mediophyceae | Eupodiscales | Eupodiscaceae | Zygoceros | 4 |
| 149325 | *Zygoceros rhombus var. trigonus* | Phytoplankton | (Cleve ex Van Heurck) Kusber & Zimmermann, 2023 | Chromista | Heterokontophyta | Mediophyceae | Eupodiscales | Eupodiscaceae | Zygoceros | 8 |
| 149050 | *Odontella aurita* | Phytoplankton | (Lyngbye) Agardh, 1832 | Chromista | Heterokontophyta | Mediophyceae | Eupodiscales | Odontellaceae | Odontella | 20 |
| 148969 | *Trieres chinensis* | Phytoplankton | (Greville) Ashworth & Theriot 2013 | Chromista | Heterokontophyta | Mediophyceae | Eupodiscales | Parodontellaceae | Trieres | 13 |
| 839991 | *Trieres mobiliensis* | Phytoplankton | (Bailey) Ashworth & Theriot 2013 | Chromista | Heterokontophyta | Mediophyceae | Eupodiscales | Parodontellaceae | Trieres | 6 |
| 839996 | *Trieres regia* | Phytoplankton | (Schultze) Ashworth & Theriot, 2013 | Chromista | Heterokontophyta | Mediophyceae | Eupodiscales | Parodontellaceae | Trieres | 13 |
| 149619 | *Cerataulina pelagica* | Phytoplankton | (Cleve) Hendey, 1937 | Chromista | Heterokontophyta | Mediophyceae | Hemiaulales | Hemiaulaceae | Cerataulina | 8 |
| 149131 | *Eucampia zodiacus* | Phytoplankton | Ehrenberg, 1839 | Chromista | Heterokontophyta | Mediophyceae | Hemiaulales | Hemiaulaceae | Eucampia | 9 |
| 149023 | *Ditylum brightwellii* | Phytoplankton | (West) Grunow, 1885 | Chromista | Heterokontophyta | Mediophyceae | Lithodesmiales | Lithodesmiaceae | Ditylum | 14 |
| 157440 | *Helicotheca tamesis* | Phytoplankton | (Shrubsole) Ricard, 1987 | Chromista | Heterokontophyta | Mediophyceae | Lithodesmiales | Lithodesmiaceae | Helicotheca | 1 |
| 149322 | *Lithodesmium undulatum* | Phytoplankton | Ehrenberg, 1839 | Chromista | Heterokontophyta | Mediophyceae | Lithodesmiales | Lithodesmiaceae | Lithodesmium | 12 |
| 148905 | *Cyclotella sp.* | Phytoplankton | (Kützing) de Brébisson, 1838 | Chromista | Heterokontophyta | Mediophyceae | Stephanodiscales | Stephanodiscaceae | Cyclotella | 4 |
| 149135 | *Lauderia annulata* | Phytoplankton | Cleve, 1873 | Chromista | Heterokontophyta | Mediophyceae | Thalassiosirales | Lauderiaceae | Lauderia | 7 |
| 149074 | *Skeletonema costatum* | Phytoplankton | (Greville) Cleve, 1873 | Chromista | Heterokontophyta | Mediophyceae | Thalassiosirales | Skeletonemaceae | Skeletonema | 4 |
| 376667 | *Skeletonema marinoi* | Phytoplankton | Sarno & Zingone, 2005 | Chromista | Heterokontophyta | Mediophyceae | Thalassiosirales | Skeletonemaceae | Skeletonema | 8 |
| 149073 | *Skeletonema sp.* | Phytoplankton | Greville, 1865 | Chromista | Heterokontophyta | Mediophyceae | Thalassiosirales | Skeletonemaceae | Skeletonema | 8 |
| 149647 | *Detonula pumila* | Phytoplankton | (Castracane) Gran, 1900 | Chromista | Heterokontophyta | Mediophyceae | Thalassiosirales | Thalassiosiraceae | Detonula | 4 |
| 148919 | *Thalassiosira decipiens* | Phytoplankton | (Grunow) Jørgensen, 1905 | Chromista | Heterokontophyta | Mediophyceae | Thalassiosirales | Thalassiosiraceae | Thalassiosira | 7 |
| 148922 | *Thalassiosira eccentrica* | Phytoplankton | (Ehrenberg) Cleve, 1904 | Chromista | Heterokontophyta | Mediophyceae | Thalassiosirales | Thalassiosiraceae | Thalassiosira | 6 |
| 148925 | *Thalassiosira minima* | Phytoplankton | Gaarder, 1951 | Chromista | Heterokontophyta | Mediophyceae | Thalassiosirales | Thalassiosiraceae | Thalassiosira | 12 |
| 148931 | *Thalassiosira nordenskioeldii* | Phytoplankton | Cleve, 1873 | Chromista | Heterokontophyta | Mediophyceae | Thalassiosirales | Thalassiosiraceae | Thalassiosira | 1 |
| 148936 | *Thalassiosira punctigera* | Phytoplankton | (Castracane) Hasle, 1983 | Chromista | Heterokontophyta | Mediophyceae | Thalassiosirales | Thalassiosiraceae | Thalassiosira | 5 |
| 148942 | *Thalassiosira rotula* | Phytoplankton | Meunier, 1910 | Chromista | Heterokontophyta | Mediophyceae | Thalassiosirales | Thalassiosiraceae | Thalassiosira | 11 |
| 148912 | *Thalassiosira sp.* | Phytoplankton | Cleve, 1873 emend. Hasle, 1973 | Chromista | Heterokontophyta | Mediophyceae | Thalassiosirales | Thalassiosiraceae | Thalassiosira | 20 |
| 233761 | *Fibrocapsa japonica* | Phytoplankton | Toriumi & Takano, 1973 | Chromista | Heterokontophyta | Raphidophyceae | Chattonellales | Fibrocapsaceae | Fibrocapsa | 9 |
| 7 | Chromista sp. | Phytoplankton | Cavalier-Smith, 1981 | Chromista | not assigned | not assigned | not assigned | not assigned | not assigned | 8 |
| 134526 | *Tetraselmis sp.* | Phytoplankton | Stein, 1878 | Plantae | Chlorophyta | Chlorodendrophyceae | Chlorodendrales | Chlorodendraceae | Tetraselmis | 8 |
| 178583 | *Chlamydomonas sp.* | Phytoplankton | Ehrenberg, 1833 | Plantae | Chlorophyta | Chlorophyceae | Chlamydomonadales | Chlamydomonadaceae | Chlamydomonas | 7 |
| 160561 | *Pediastrum sp.* | Phytoplankton | Meyen, 1829 | Plantae | Chlorophyta | Chlorophyceae | Sphaeropleales | Hydrodictyaceae | Pediastrum | 2 |
| 160541 | Scenedesmaceae sp. | Phytoplankton | Oltmanns, 1904 | Plantae | Chlorophyta | Chlorophyceae | Sphaeropleales | Scenedesmaceae | not assigned | 7 |
| 160602 | *Scenedesmus sp.* | Phytoplankton | Meyen, 1829 | Plantae | Chlorophyta | Chlorophyceae | Sphaeropleales | Scenedesmaceae | Scenedesmus | 1 |
| 160590 | *Monoraphidium sp.* | Phytoplankton | Komárková-Legnerová, 1969 | Plantae | Chlorophyta | Chlorophyceae | Sphaeropleales | Selenastraceae | Monoraphidium | 1 |
| 134564 | *Micromonas pusilla* | Phytoplankton | (Butcher) Manton & Parke, 1960 | Plantae | Chlorophyta | Mamiellophyceae | Mamiellales | Mamiellaceae | Micromonas | 3 |
| 160595 | *Pterosperma sp.* | Phytoplankton | Pouchet, 1893 | Plantae | Chlorophyta | Pyramimonadophyceae | Pyramimonadales | Pterospermataceae | Pterosperma | 5 |
| 134545 | *Cymbomonas tetramitiformis* | Phytoplankton | Schiller, 1913 | Plantae | Chlorophyta | Pyramimonadophyceae | Pyramimonadales | Pyramimonadaceae | Cymbomonas | 1 |
| 160513 | *Pyramimonas longicauda* | Phytoplankton | van Meel, 1969 | Plantae | Chlorophyta | Pyramimonadophyceae | Pyramimonadales | Pyramimonadaceae | Pyramimonas | 4 |
| 134529 | *Pyramimonas sp.* | Phytoplankton | Schmarda, 1849 | Plantae | Chlorophyta | Pyramimonadophyceae | Pyramimonadales | Pyramimonadaceae | Pyramimonas | 8 |
| 425976 | *Glaux maritima* | Plants | Linnaeus, 1753 | Plantae | Tracheophyta | Equisetopsida | Ericales | Primulaceae | Glaux | 5 |
| not assigned | *Trifolium arvense* | Plants | Linnaeus, 1753 | Plantae | Tracheophyta | Equisetopsida | Fabales | Fabaceae | Trifolium | 1 |
| 993805 | *Trifolium repens* | Plants | Linnaeus, 1753 | Plantae | Tracheophyta | Equisetopsida | Fabales | Fabaceae | Trifolium | 1 |
| not assigned | *Linaria vulgaris* | Plants | Miller, 1768 | Plantae | Tracheophyta | Equisetopsida | Lamiales | Plantaginaceae | Linaria | 1 |
| 425922 | *Plantago coronopus* | Plants | Linnaeus, 1753 | Plantae | Tracheophyta | Equisetopsida | Lamiales | Plantaginaceae | Plantago | 1 |
| 426080 | *Plantago maritima* | Plants | Barton, 1815 | Plantae | Tracheophyta | Equisetopsida | Lamiales | Plantaginaceae | Plantago | 5 |
| not assigned | *Argentina anserina* | Plants | (L.) Rydberg, 1898 | Plantae | Tracheophyta | Equisetopsida | Rosales | Rosaceae | Argentina | 1 |
| not assigned | *Sedum acre* | Plants | Linnaeus, 1753 | Plantae | Tracheophyta | Equisetopsida | Saxifragales | Crassulaceae | Sedum | 5 |
| 425961 | *Carex extensa* | Plants | Goodenough, 1794 | Plantae | Tracheophyta | Liliopsida | Poales | Cyperaceae | Carex | 1 |
| 394967 | *Juncus gerardii* | Plants | Loisel, 1809 | Plantae | Tracheophyta | Liliopsida | Poales | Juncaceae | Juncus | 3 |
| 426076 | *Juncus maritimus* | Plants | Lamarck, 1789 | Plantae | Tracheophyta | Liliopsida | Poales | Juncaceae | Juncus | 1 |
| 993840 | *Agrostis stolonifera* | Plants | Linnaeus, 1753 | Plantae | Tracheophyta | Liliopsida | Poales | Poaceae | Agrostis | 2 |
| 431933 | *Elytrigia atherica/mar* | Plants | (Link) Carreras Mart., 1987 | Plantae | Tracheophyta | Liliopsida | Poales | Poaceae | Elymus | 8 |
| 431962 | *Festuca rubra* | Plants | Linnaeus, 1753 | Plantae | Tracheophyta | Liliopsida | Poales | Poaceae | Festuca | 21 |
| 403907 | *Puccinellia maritima* | Plants | (Huds.) Parlatore, 1850 | Plantae | Tracheophyta | Liliopsida | Poales | Poaceae | Puccinellia | 11 |
| 234043 | *Sporobolus anglicus* | Plants | (Hubb.) Peterson & Saarela, 2014 | Plantae | Tracheophyta | Liliopsida | Poales | Poaceae | Sporobolus | 8 |
| not assigned | *Thinopyrum acutum* | Plants | (DC.) Banfi, 2018 | Plantae | Tracheophyta | Liliopsida | Poales | Poaceae | Thinopyrum | 14 |
| not assigned | *Thinopyrum junceum* | Plants | (L.) Á.Löve, 1980 | Plantae | Tracheophyta | Liliopsida | Poales | Poaceae | Thinopyrum | 1 |
| 426006 | *Triglochin maritima* | Plants | Linnaeus, 1753 | Plantae | Tracheophyta | Magnoliopsida | Alismatales | Juncaginaceae | Triglochin | 4 |
| 669496 | *Nanozostera noltei* | Plants | (Hornemann) Tomlinson & Posluszny, 2001 | Plantae | Tracheophyta | Magnoliopsida | Alismatales | Zosteraceae | Nanozostera | 2 |
| 495077 | *Zostera marina* | Plants | Linnaeus, 1753 | Plantae | Tracheophyta | Magnoliopsida | Alismatales | Zosteraceae | Zostera | 2 |
| 426065 | *Artemisia maritima* | Plants | Linnaeus, 1753 | Plantae | Tracheophyta | Magnoliopsida | Asterales | Asteraceae | Artemisia | 17 |
| 403905 | *Tripolium pannonicum* | Plants | (Jacq.) Dobrocz., 1962 | Plantae | Tracheophyta | Magnoliopsida | Asterales | Compositae | Tripolium | 5 |
| 431936 | *Atriplex portulacoides* | Plants | Linnaeus, 1753 | Plantae | Tracheophyta | Magnoliopsida | Caryophyllales | Amaranthaceae | Atriplex | 18 |
| not assigned | *Atriplex prostrata* | Plants | Boucher ex DC, 1805 | Plantae | Tracheophyta | Magnoliopsida | Caryophyllales | Amaranthaceae | Atriplex | 8 |
| 425952 | *Salicornia europaea* | Plants | Linnaeus, 1753 | Plantae | Tracheophyta | Magnoliopsida | Caryophyllales | Amaranthaceae | Salicornia | 12 |
| 425938 | *Suaeda maritima* | Plants | (L.) Dumortier, 1827 | Plantae | Tracheophyta | Magnoliopsida | Caryophyllales | Amaranthaceae | Suaeda | 16 |
| 438825 | *Arenaria serpyllifolia* | Plants | Linnaeus, 1753 | Plantae | Tracheophyta | Magnoliopsida | Caryophyllales | Caryophyllaceae | Arenaria | 1 |
| 412894 | *Honckenya peploides* | Plants | (L.) Ehrhart, 1783 | Plantae | Tracheophyta | Magnoliopsida | Caryophyllales | Caryophyllaceae | Honckenya | 1 |
| 426008 | *Sagina nodosa* | Plants | (L.) Fenzl, 1833 | Plantae | Tracheophyta | Magnoliopsida | Caryophyllales | Caryophyllaceae | Sagina | 1 |
| 425804 | *Spergularia media* | Plants | (L.) Presl, 1826 | Plantae | Tracheophyta | Magnoliopsida | Caryophyllales | Caryophyllaceae | Spergularia | 4 |
| 182769 | *Armeria maritima* | Plants | (Miller) Willdenow, 1809 | Plantae | Tracheophyta | Magnoliopsida | Caryophyllales | Plumbaginaceae | Armeria | 2 |
| 232102 | *Limonium vulgare* | Plants | Miller, 1768 | Plantae | Tracheophyta | Magnoliopsida | Caryophyllales | Plumbaginaceae | Limonium | 21 |
| 562870 | Choanozoa sp. |  | Cavalier-Smith, 1981 | Protozoa | Choanozoa | not assigned | not assigned | not assigned | not assigned | 8 |
| 110652 | *Eutreptiella gymnastica* | Phytoplankton | Throndsen, 1969 | Protozoa | Euglenophyta | Euglenophyceae | Eutreptiales | Eutreptiaceae | Eutreptiella | 3 |
| 160556 | *Eutreptiella marina* | Phytoplankton | da Cunha, 1914 | Protozoa | Euglenophyta | Euglenophyceae | Eutreptiales | Eutreptiaceae | Eutreptiella | 3 |
| 17657 | *Eutreptiella sp.* | Phytoplankton | A.M.Cunha, 1913 | Protozoa | Euglenophyta | Euglenophyceae | Eutreptiales | Eutreptiaceae | Eutreptiella | 16 |
| 5 | Protozoa sp. |  | Owen, 1858 | Protozoa | not assigned | not assigned | not assigned | not assigned | not assigned | 8 |

#### **Table S2**: Number of entries for each ecosystem component and phylogenetic level. Entries identified only to genus level were included as “Genera sp.” at species level. The ecosystem component of “Plants” includes both salt marsh plants and seagrasses. Macroinvertebrates show a lower number of genera than families as three genera could not be assigned. Abbreviations are as follows: Phytoplankton (Phytopl.), macrozoobenthos (MZB), zooplankton (Zoopl.).

|  | **Phytopl.** | **MZB** | **Birds** | **Fish** | **Plants** | **Zoopl.** |
| --- | --- | --- | --- | --- | --- | --- |
| **Phyla** | 10 | 7 | 1 | 1 | 1 | 4 |
| **Classes** | 19 | 11 | 1 | 1 | 3 | 5 |
| **Orders** | 48 | 27 | 6 | 12 | 9 | 8 |
| **Families** | 69 | 51 | 10 | 25 | 15 | 14 |
| **Genera** | 97 | 72 | 34 | 33 | 28 | 14 |
| **Species** | 161 | 96 | 57 | 40 | 33 | 14 |

####

#### **Table S3.** Multinomial model output for the temporal trends. Taxa are ordered by taxonomic level. In case the polynomial model was a better fit, 2nd order positive and negative coefficients are also given. Significant p-values (p < 0.05) are indicated in bold.

| Phylum | Taxa | Coefficient | Estimate | Std. Error | z value | Pr(>\|z\|) |
| --- | --- | --- | --- | --- | --- | --- |
| Annelida | Annelida (Phylum) | Positive | -29.75 | 3.24 | -9.17 | **0** |
|  |  | Positive 2nd order | 5.68 | 3.09 | 1.84 | 0.07 |
|  |  | Negative | -30.81 | 2.76 | -11.17 | **0** |
|  |  | Negative 2nd order | -3.57 | 2.67 | -1.34 | 0.18 |
|  | Clitellata (Class) | Positive | -22.40 | 3.63 | -6.18 | **0** |
|  |  | Positive 2nd order | 12.34 | 3.16 | 3.91 | **0** |
|  |  | Negative | -6.23 | 3.26 | -1.91 | 0.06 |
|  |  | Negative 2nd order | -4.90 | 2.83 | -1.73 | 0.08 |
|  | Tubificida (Order) | Positive | -24.46 | 3.61 | -6.79 | **0** |
|  |  | Positive 2nd order | 10.26 | 3.02 | 3.40 | **0** |
|  |  | Negative | -6.26 | 3.28 | -1.91 | 0.06 |
|  |  | Negative 2nd order | -5.06 | 2.91 | -1.74 | 0.08 |
|  | Naididae (Family) | Positive | -24.46 | 3.61 | -6.79 | **0** |
|  |  | Positive 2nd order | 10.26 | 3.02 | 3.40 | **0** |
|  |  | Negative | -6.26 | 3.28 | -1.91 | 0.06 |
|  |  | Negative 2nd order | -5.06 | 2.91 | -1.74 | 0.08 |
|  | Baltidrilus (Genus) | Positive | -0.04 | 0.11 | -0.41 | 0.68 |
|  |  | Negative | -0.01 | 0.10 | -0.13 | 0.9 |
|  | Paranais (Genus) | Negative | 0.02 | 0.15 | 0.10 | 0.92 |
|  | Tubificoides (Genus) | Positive | -23.97 | 4.54 | -5.28 | **0** |
|  |  | Positive 2nd order | 16.43 | 4.02 | 4.09 | **0** |
|  |  | Negative | -9.06 | 5.48 | -1.66 | 0.1 |
|  |  | Negative 2nd order | -0.22 | 4.94 | -0.04 | 0.96 |
|  | Polychaeta (Class) | Positive | -25.64 | 3.24 | -7.91 | **0** |
|  |  | Positive 2nd order | 3.90 | 3.09 | 1.26 | 0.21 |
|  |  | Negative | -29.33 | 2.73 | -10.75 | **0** |
|  |  | Negative 2nd order | -2.97 | 2.65 | -1.12 | 0.26 |
|  | Capitellida (Order) | Positive | 2.62 | 2.92 | 0.90 | 0.37 |
|  |  | Positive 2nd order | 6.62 | 2.90 | 2.28 | **0.02** |
|  |  | Negative | -10.32 | 2.56 | -4.02 | **0** |
|  |  | Negative 2nd order | -1.66 | 2.50 | -0.67 | 0.51 |
|  | Arenicolidae (Family) | Positive | 0.00 | 0.05 | -0.07 | 0.95 |
|  |  | Negative | 0.00 | 0.05 | -0.04 | 0.97 |
|  | Arenicola (Genus) | Positive | 0.00 | 0.05 | -0.07 | 0.95 |
|  |  | Negative | 0.00 | 0.05 | -0.04 | 0.97 |
|  | Capitellidae (Family) | Positive | 6.26 | 3.11 | 2.01 | **0.04** |
|  |  | Positive 2nd order | 2.68 | 3.07 | 0.88 | 0.38 |
|  |  | Negative | -10.36 | 2.64 | -3.92 | **0** |
|  |  | Negative 2nd order | -5.48 | 2.55 | -2.15 | **0.03** |
|  | Capitella (Genus) | Positive | 56.82 | 280.50 | 0.20 | 0.84 |
|  |  | Positive 2nd order | -53.09 | 174.61 | -0.30 | 0.76 |
|  |  | Negative | -33.55 | 7.06 | -4.75 | **0** |
|  |  | Negative 2nd order | 15.89 | 6.66 | 2.39 | **0.02** |
|  | Heteromastus (Genus) | Positive | 0.03 | 0.04 | 0.68 | 0.49 |
|  |  | Negative | -0.01 | 0.04 | -0.21 | 0.83 |
|  | Phyllodocida (Order) | Positive | -0.03 | 0.00 | -7.32 | **0** |
|  |  | Negative | -0.02 | 0.00 | -7.41 | **0** |
|  | Nephtyidae (Family) | Positive | 4.96 | 20.55 | 0.24 | 0.81 |
|  |  | Positive 2nd order | -45.47 | 13.54 | -3.36 | **0** |
|  |  | Negative | -30.93 | 3.81 | -8.11 | **0** |
|  |  | Negative 2nd order | 5.63 | 3.29 | 1.71 | 0.09 |
|  | Nephtys (Genus) | Positive | 4.96 | 20.55 | 0.24 | 0.81 |
|  |  | Positive 2nd order | -45.47 | 13.54 | -3.36 | **0** |
|  |  | Negative | -30.93 | 3.81 | -8.11 | **0** |
|  |  | Negative 2nd order | 5.63 | 3.29 | 1.71 | 0.09 |
|  | Nereididae (Family) | Positive | 3.89 | 3.89 | 1.00 | 0.32 |
|  |  | Positive 2nd order | 2.19 | 3.65 | 0.60 | 0.55 |
|  |  | Negative | 0.63 | 2.89 | 0.22 | 0.83 |
|  |  | Negative 2nd order | 6.83 | 2.80 | 2.44 | **0.01** |
|  | Alitta (Genus) | Positive | -8.09 | 4.50 | -1.80 | 0.07 |
|  |  | Positive 2nd order | 14.17 | 4.82 | 2.94 | **0** |
|  |  | Negative | 18.86 | 10.46 | 1.80 | 0.07 |
|  |  | Negative 2nd order | -0.60 | 8.34 | -0.07 | 0.94 |
|  | Hediste (Genus) | Positive | 0.02 | 0.05 | 0.37 | 0.71 |
|  |  | Negative | 0.00 | 0.05 | -0.07 | 0.94 |
|  | Phyllodocidae (Family) | Positive | -24.93 | 3.95 | -6.31 | **0** |
|  |  | Positive 2nd order | 11.56 | 3.79 | 3.05 | **0** |
|  |  | Negative | -15.58 | 3.40 | -4.58 | **0** |
|  |  | Negative 2nd order | 4.85 | 3.23 | 1.50 | 0.13 |
|  | Eteone (Genus) | Positive | -0.06 | 0.05 | -1.06 | 0.29 |
|  |  | Negative | -0.04 | 0.05 | -0.74 | 0.46 |
|  | Phyllodoce (Genus) | Positive | 0.13 | 0.15 | 0.85 | 0.4 |
|  |  | Negative | -0.01 | 0.06 | -0.23 | 0.82 |
|  | Polynoidae (Family) | Positive | -1.12 | 2.38 | -0.47 | 0.64 |
|  |  | Positive 2nd order | 2.32 | 2.43 | 0.95 | 0.34 |
|  |  | Negative | 29.16 | 9.49 | 3.07 | **0** |
|  |  | Negative 2nd order | -18.18 | 6.85 | -2.66 | **0.01** |
|  | Bylgides (Genus) | Positive | 0.00 | 0.10 | -0.04 | 0.96 |
|  |  | Negative | 0.05 | 0.12 | 0.40 | 0.69 |
|  | Syllidae (Family) | Positive | -0.49 | 0.82 | -0.60 | 0.55 |
|  | Myrianida (Genus) | Positive | -0.49 | 0.82 | -0.60 | 0.55 |
|  | Spionida (Order) | Positive | 0.00 | 0.01 | -0.04 | 0.97 |
|  |  | Negative | -0.01 | 0.01 | -2.24 | **0.02** |
|  | Spionidae (Family) | Positive | 0.00 | 0.01 | -0.04 | 0.97 |
|  |  | Negative | -0.01 | 0.01 | -2.24 | **0.02** |
|  | Marenzelleria (Genus) | Positive | -0.04 | 0.08 | -0.52 | 0.6 |
|  |  | Negative | -0.04 | 0.09 | -0.50 | 0.61 |
|  | Polydora (Genus) | Positive | 0.03 | 0.10 | 0.30 | 0.77 |
|  | Pygospio (Genus) | Positive | 0.00 | 0.06 | -0.01 | 0.99 |
|  |  | Negative | -0.02 | 0.05 | -0.31 | 0.75 |
|  | Scolelepis (Genus) | Negative | 0.00 | 0.01 | -0.08 | 0.94 |
|  | Spio (Genus) | Positive | -0.04 | 0.11 | -0.36 | 0.72 |
|  |  | Negative | 0.01 | 0.16 | 0.05 | 0.96 |
|  | Spiophanes (Genus) | Positive | 0.03 | 0.22 | 0.12 | 0.91 |
|  | Streblospio (Genus) | Positive | 0.06 | 0.07 | 0.90 | 0.37 |
|  |  | Negative | 0.03 | 0.02 | 1.97 | 0.05 |
|  | Terebellida (Order) | Positive | -0.04 | 0.01 | -5.71 | **0** |
|  |  | Negative | -0.01 | 0.01 | -1.09 | 0.28 |
|  | Ampharetidae (Family) | Positive | 0.06 | 0.31 | 0.21 | 0.83 |
|  | Ampharete (Genus) | Positive | 0.06 | 0.31 | 0.21 | 0.83 |
|  | Cirratulidae (Family) | Positive | -46.21 | 8.25 | -5.60 | **0** |
|  |  | Positive 2nd order | 19.99 | 5.85 | 3.42 | **0** |
|  |  | Negative | -28.45 | 9.87 | -2.88 | **0** |
|  |  | Negative 2nd order | 19.71 | 7.60 | 2.59 | **0.01** |
|  | Aphelochaeta (Genus) | Positive | 0.00 | 0.33 | 0.00 | 1 |
|  |  | Negative | -0.10 | 0.33 | -0.30 | 0.77 |
|  | Tharyx (Genus) | Positive | -0.09 | 0.10 | -0.90 | 0.37 |
|  |  | Negative | 0.02 | 0.16 | 0.15 | 0.88 |
|  | Pectinariidae (Family) | Positive | 0.00 | 0.43 | 0.00 | 1 |
|  | Lagis (Genus) | Positive | 0.00 | 0.43 | 0.00 | 1 |
|  | Terebellidae (Family) | Positive | -0.01 | 0.06 | -0.16 | 0.88 |
|  |  | Negative | -0.01 | 0.06 | -0.17 | 0.87 |
|  | Lanice (Genus) | Positive | -0.01 | 0.06 | -0.16 | 0.88 |
|  |  | Negative | -0.01 | 0.06 | -0.17 | 0.87 |
|  | Magelonidae (Family) | Positive | -17.70 | 100.98 | -0.18 | 0.86 |
|  |  | Positive 2nd order | 4.41 | 46.48 | 0.09 | 0.92 |
|  |  | Negative | -104.04 | 39.42 | -2.64 | **0.01** |
|  |  | Negative 2nd order | 38.51 | 18.78 | 2.05 | **0.04** |
|  | Magelona (Genus) | Positive | -17.70 | 100.98 | -0.18 | 0.86 |
|  |  | Positive 2nd order | 4.41 | 46.48 | 0.09 | 0.92 |
|  |  | Negative | -104.04 | 39.42 | -2.64 | **0.01** |
|  |  | Negative 2nd order | 38.51 | 18.78 | 2.05 | **0.04** |
|  | Orbiniidae (Family) | Positive | -0.05 | 0.05 | -1.03 | 0.3 |
|  |  | Negative | -0.04 | 0.05 | -0.70 | 0.48 |
|  | Scoloplos (Genus) | Positive | -0.05 | 0.05 | -1.03 | 0.3 |
|  |  | Negative | -0.04 | 0.05 | -0.70 | 0.48 |
| Arthropoda | Arthropoda (Phylum) | Positive | -4.15 | 3.10 | -1.34 | 0.18 |
|  |  | Positive 2nd order | -10.11 | 3.01 | -3.36 | **0** |
|  |  | Negative | -9.09 | 2.54 | -3.58 | **0** |
|  |  | Negative 2nd order | -3.81 | 2.45 | -1.55 | 0.12 |
|  | Copepoda (Class) | Positive | 0.00 | 0.29 | 0.00 | 1 |
|  |  | Negative | 0.00 | 0.18 | 0.00 | 1 |
|  | Calanoida (Order) | Positive | 0.00 | 0.31 | 0.00 | 1 |
|  |  | Negative | 0.00 | 0.18 | 0.00 | 1 |
|  | Centropagidae (Family) | Negative | 0.00 | 0.65 | 0.00 | 1 |
|  | Centropages (Genus) | Negative | 0.00 | 0.65 | 0.00 | 1 |
|  | Paracalanidae (Family) | Positive | 0.00 | 0.77 | 0.00 | 1 |
|  |  | Negative | 0.00 | 0.82 | 0.00 | 1 |
|  | Paracalanus (Genus) | Positive | 0.00 | 0.77 | 0.00 | 1 |
|  |  | Negative | 0.00 | 0.82 | 0.00 | 1 |
|  | Temoridae (Family) | Negative | 0.00 | 0.65 | 0.00 | 1 |
|  | Temora (Genus) | Negative | 0.00 | 0.65 | 0.00 | 1 |
|  | Malacostraca (Class) | Positive | -4.35 | 3.04 | -1.43 | 0.15 |
|  |  | Positive 2nd order | -10.31 | 2.95 | -3.49 | **0** |
|  |  | Negative | -8.77 | 2.49 | -3.52 | **0** |
|  |  | Negative 2nd order | -4.19 | 2.44 | -1.72 | 0.09 |
|  | Amphipoda (Order) | Positive | -1.91 | 3.26 | -0.59 | 0.56 |
|  |  | Positive 2nd order | -4.85 | 3.24 | -1.50 | 0.13 |
|  |  | Negative | 0.46 | 2.47 | 0.19 | 0.85 |
|  |  | Negative 2nd order | -4.91 | 2.44 | -2.02 | **0.04** |
|  | Bathyporeiidae (Family) | Negative | -0.02 | 0.01 | -1.54 | 0.12 |
|  | Bathyporeia (Genus) | Negative | -0.02 | 0.01 | -1.54 | 0.12 |
|  | Corophiidae (Family) | Positive | -5.52 | 2.65 | -2.08 | **0.04** |
|  |  | Positive 2nd order | -5.56 | 2.61 | -2.13 | **0.03** |
|  |  | Negative | 6.74 | 2.76 | 2.44 | **0.01** |
|  |  | Negative 2nd order | -10.64 | 2.75 | -3.87 | **0** |
|  | Corophium (Genus) | Positive | -5.52 | 2.65 | -2.08 | **0.04** |
|  |  | Positive 2nd order | -5.56 | 2.61 | -2.13 | **0.03** |
|  |  | Negative | 6.74 | 2.76 | 2.44 | **0.01** |
|  |  | Negative 2nd order | -10.64 | 2.75 | -3.87 | **0** |
|  | Gammaridae (Family) | Negative | -0.03 | 0.17 | -0.15 | 0.88 |
|  | Gammarus (Genus) | Negative | -0.03 | 0.17 | -0.15 | 0.88 |
|  | Oedicerotidae (Family) | Positive | -0.02 | 0.45 | -0.04 | 0.97 |
|  | Pontocrates (Genus) | Positive | -0.02 | 0.45 | -0.04 | 0.97 |
|  | Urothoidae (Family) | Positive | 0.05 | 0.11 | 0.44 | 0.66 |
|  |  | Negative | 0.02 | 0.10 | 0.18 | 0.86 |
|  | Urothoe (Genus) | Positive | 0.05 | 0.11 | 0.44 | 0.66 |
|  |  | Negative | 0.02 | 0.10 | 0.18 | 0.86 |
|  | Decapoda (Order) | Positive | -3.48 | 2.92 | -1.19 | 0.23 |
|  |  | Positive 2nd order | -9.10 | 2.88 | -3.16 | **0** |
|  |  | Negative | -9.77 | 2.41 | -4.06 | **0** |
|  |  | Negative 2nd order | -1.58 | 2.39 | -0.66 | 0.51 |
|  | Carcinidae (Family) | Positive | 0.01 | 0.05 | 0.16 | 0.87 |
|  |  | Negative | -0.01 | 0.04 | -0.35 | 0.72 |
|  | Carcinus (Genus) | Positive | 0.01 | 0.05 | 0.16 | 0.87 |
|  |  | Negative | -0.01 | 0.04 | -0.35 | 0.72 |
|  | Crangonidae (Family) | Positive | -0.02 | 0.01 | -3.47 | **0** |
|  |  | Negative | -0.01 | 0.00 | -2.73 | **0.01** |
|  | Crangon (Genus) | Positive | -0.02 | 0.01 | -3.47 | **0** |
|  |  | Negative | -0.01 | 0.00 | -2.73 | **0.01** |
|  | Paguridae (Family) | Positive | 0.01 | 0.12 | 0.07 | 0.94 |
|  | Polybiidae (Family) | Positive | -0.04 | 0.10 | -0.36 | 0.72 |
|  |  | Negative | -0.03 | 0.11 | -0.24 | 0.81 |
|  | Liocarcinus (Genus) | Positive | -0.04 | 0.10 | -0.36 | 0.72 |
|  |  | Negative | -0.03 | 0.11 | -0.24 | 0.81 |
|  | Mysida (Order) | Positive | 0.00 | 0.10 | -0.01 | 0.99 |
|  |  | Negative | -0.01 | 0.09 | -0.14 | 0.89 |
|  | Mysidae (Family) | Positive | 0.00 | 0.10 | -0.01 | 0.99 |
|  |  | Negative | -0.01 | 0.09 | -0.14 | 0.89 |
|  | Neomysis (Genus) | Positive | 0.00 | 0.43 | 0.00 | 1 |
|  | Schistomysis (Genus) | Negative | -0.01 | 0.33 | -0.02 | 0.99 |
|  | Thecostraca (Class) | Positive | 7.68 | 4.73 | 1.62 | 0.1 |
|  |  | Positive 2nd order | -7.33 | 4.67 | -1.57 | 0.12 |
|  |  | Negative | -0.41 | 2.34 | -0.17 | 0.86 |
|  |  | Negative 2nd order | 3.24 | 2.36 | 1.37 | 0.17 |
|  | Balanomorpha (Order) | Positive | 7.68 | 4.73 | 1.62 | 0.1 |
|  |  | Positive 2nd order | -7.33 | 4.67 | -1.57 | 0.12 |
|  |  | Negative | -0.41 | 2.34 | -0.17 | 0.86 |
|  |  | Negative 2nd order | 3.24 | 2.36 | 1.37 | 0.17 |
|  | Balanidae (Family) | Positive | 0.02 | 0.15 | 0.16 | 0.87 |
|  |  | Negative | 0.00 | 0.13 | -0.03 | 0.97 |
|  | Balanus (Genus) | Positive | 0.02 | 0.15 | 0.16 | 0.87 |
|  |  | Negative | 0.00 | 0.13 | -0.03 | 0.97 |
| Cercozoa | Cercozoa (Phylum) | Negative | 0.00 | 0.32 | 0.00 | 1 |
|  | Thecofilosea (Class) | Negative | 0.00 | 0.32 | 0.00 | 1 |
|  | Cryomonadida (Order) | Negative | 0.00 | 0.87 | 0.00 | 1 |
|  | Protaspidae (Family) | Negative | 0.00 | 0.87 | 0.00 | 1 |
|  | Cryothecomonas (Genus) | Negative | 0.00 | 0.87 | 0.00 | 1 |
| Chlorophyta | Chlorophyta (Phylum) | Positive | 0.00 | 0.04 | 0.01 | 0.99 |
|  |  | Negative | 0.01 | 0.09 | 0.14 | 0.89 |
|  | Chlorodendrophyceae (Class) | Negative | 0.00 | 0.43 | 0.00 | 1 |
|  | Clorodendrales (Order) | Negative | 0.00 | 0.43 | 0.00 | 1 |
|  | Chlorodendraceae (Family) | Negative | 0.00 | 0.43 | 0.00 | 1 |
|  | Tetraselmis (Genus) | Negative | 0.00 | 0.43 | 0.00 | 1 |
|  | Chlorophyceae (Class) | Negative | 33.57 | 28.52 | 1.18 | 0.24 |
|  |  | Negative 2nd order | -32.71 | 25.72 | -1.27 | 0.2 |
|  | Chlamydomonadales (Order) | Negative | 0.00 | 0.56 | 0.00 | 1 |
|  | Chlamydomonadaceae (Family) | Negative | 0.00 | 0.56 | 0.00 | 1 |
|  | Chlamydomonas (Genus) | Negative | 0.00 | 0.56 | 0.00 | 1 |
|  | Pyramimonadophyceae (Class) | Positive | 0.00 | 0.04 | -0.06 | 0.96 |
|  |  | Negative | 0.01 | 0.12 | 0.04 | 0.96 |
|  | Pyramimonadales (Order) | Positive | 0.00 | 0.04 | -0.06 | 0.96 |
|  |  | Negative | 0.01 | 0.12 | 0.04 | 0.96 |
|  | Pterospermataceae (Family) | Negative | 0.00 | 0.61 | 0.00 | 1 |
|  | Pterosperma (Genus) | Negative | 0.00 | 0.61 | 0.00 | 1 |
|  | Pyramimonadaceae (Family) | Positive | 0.00 | 0.04 | -0.04 | 0.97 |
|  |  | Negative | 0.01 | 0.14 | 0.07 | 0.94 |
|  | Pyramimonas (Genus) | Positive | -0.02 | 2.31 | -0.01 | 0.99 |
|  |  | Positive 2nd order | 0.00 | 2.37 | 0.00 | 1 |
|  |  | Negative | 46.21 | 35.47 | 1.30 | 0.19 |
|  |  | Negative 2nd order | -44.92 | 31.80 | -1.41 | 0.16 |
| Choanozoa | Choanozoa (Phylum) | Negative | 0.00 | 0.43 | 0.00 | 1 |
| Chordata | Chordata (Phylum) | Positive | -45.79 | 4.97 | -9.22 | **0** |
|  |  | Positive 2nd order | -1.58 | 3.28 | -0.48 | 0.63 |
|  |  | Negative | 24.27 | 5.06 | 4.79 | **0** |
|  |  | Negative 2nd order | 15.51 | 3.34 | 4.64 | **0** |
|  | Aves (Class) | Positive | -39.81 | 4.62 | -8.62 | **0** |
|  |  | Positive 2nd order | -8.11 | 3.89 | -2.09 | **0.04** |
|  |  | Negative | 24.60 | 4.57 | 5.39 | **0** |
|  |  | Negative 2nd order | 23.36 | 3.77 | 6.19 | **0** |
|  | Anseriformes (Order) | Positive | 14.22 | 5.61 | 2.53 | **0.01** |
|  |  | Positive 2nd order | 1.52 | 3.70 | 0.41 | 0.68 |
|  |  | Negative | 23.96 | 5.98 | 4.01 | **0** |
|  |  | Negative 2nd order | -7.79 | 4.06 | -1.92 | 0.05 |
|  | Anatidae (Family) | Positive | 14.22 | 5.61 | 2.53 | **0.01** |
|  |  | Positive 2nd order | 1.52 | 3.70 | 0.41 | 0.68 |
|  |  | Negative | 23.96 | 5.98 | 4.01 | **0** |
|  |  | Negative 2nd order | -7.79 | 4.06 | -1.92 | 0.05 |
|  | Anas (Genus) | Positive | 4.22 | 4.00 | 1.05 | 0.29 |
|  |  | Positive 2nd order | -7.72 | 4.17 | -1.85 | 0.06 |
|  | Anser (Genus) | Positive | 0.00 | 0.10 | 0.00 | 1 |
|  |  | Negative | 0.00 | 0.11 | 0.00 | 1 |
|  | Branta (Genus) | Positive | 5.71 | 2.51 | 2.27 | **0.02** |
|  |  | Positive 2nd order | -9.68 | 2.69 | -3.60 | **0** |
|  | Mareca (Genus) | Negative | 6.76 | 3.06 | 2.21 | **0.03** |
|  |  | Negative 2nd order | -10.36 | 3.29 | -3.15 | **0** |
|  | Somateria (Genus) | Positive | 0.01 | 0.11 | 0.12 | 0.91 |
|  |  | Negative | 0.00 | 0.10 | -0.01 | 0.99 |
|  | Spatula (Genus) | Positive | -0.01 | 0.14 | -0.07 | 0.95 |
|  | Tadorna (Genus) | Positive | 0.00 | 0.04 | 0.03 | 0.98 |
|  |  | Negative | 0.02 | 0.04 | 0.47 | 0.64 |
|  | Charadriiformes (Order) | Positive | -42.61 | 4.21 | -10.11 | **0** |
|  |  | Positive 2nd order | -13.67 | 3.84 | -3.56 | **0** |
|  |  | Negative | 17.90 | 4.10 | 4.36 | **0** |
|  |  | Negative 2nd order | 24.89 | 3.68 | 6.77 | **0** |
|  | Charadriidae (Family) | Positive | -13.90 | 3.38 | -4.11 | **0** |
|  |  | Positive 2nd order | -6.16 | 3.25 | -1.89 | 0.06 |
|  |  | Negative | -2.88 | 3.26 | -0.88 | 0.38 |
|  |  | Negative 2nd order | 4.66 | 3.07 | 1.52 | 0.13 |
|  | Charadrius (Genus) | Positive | -2.83 | 3.09 | -0.92 | 0.36 |
|  |  | Positive 2nd order | -1.97 | 3.11 | -0.63 | 0.53 |
|  |  | Negative | 3.56 | 2.67 | 1.33 | 0.18 |
|  |  | Negative 2nd order | 8.68 | 2.67 | 3.25 | **0** |
|  | Haematopus (Genus) | Positive | -0.03 | 0.04 | -0.77 | 0.44 |
|  |  | Negative | -0.03 | 0.04 | -0.61 | 0.54 |
|  | Pluvialis (Genus) | Positive | 0.00 | 0.01 | 0.00 | 1 |
|  | Vanellus (Genus) | Positive | 0.01 | 0.14 | 0.07 | 0.95 |
|  | Laridae (Family) | Positive | -8.76 | 4.69 | -1.87 | 0.06 |
|  |  | Positive 2nd order | -3.13 | 4.99 | -0.63 | 0.53 |
|  |  | Negative | 23.99 | 4.61 | 5.21 | **0** |
|  |  | Negative 2nd order | 35.00 | 4.84 | 7.22 | **0** |
|  | Larus (Genus) | Positive | 12.44 | 9.96 | 1.25 | 0.21 |
|  |  | Positive 2nd order | 18.06 | 9.95 | 1.81 | 0.07 |
|  |  | Negative | 66.31 | 10.17 | 6.52 | **0** |
|  |  | Negative 2nd order | 38.48 | 10.28 | 3.74 | **0** |
|  | Sterna (Genus) | Positive | 10.51 | 3.09 | 3.40 | **0** |
|  |  | Positive 2nd order | -7.16 | 3.09 | -2.32 | **0.02** |
|  |  | Negative | -3.35 | 2.93 | -1.14 | 0.25 |
|  |  | Negative 2nd order | 4.31 | 2.88 | 1.50 | 0.13 |
|  | Sternula (Genus) | Positive | -0.01 | 0.04 | -0.21 | 0.84 |
|  |  | Negative | -0.01 | 0.04 | -0.22 | 0.83 |
|  | Recurvirostridae (Family) | Positive | 0.00 | 0.07 | -0.04 | 0.97 |
|  |  | Negative | 0.06 | 0.08 | 0.84 | 0.4 |
|  | Recurvirostra (Genus) | Positive | 0.00 | 0.07 | -0.04 | 0.97 |
|  |  | Negative | 0.06 | 0.08 | 0.84 | 0.4 |
|  | Scolopacidae (Family) | Positive | -123.89 | 16.12 | -7.69 | **0** |
|  |  | Positive 2nd order | 40.27 | 11.42 | 3.53 | **0** |
|  |  | Negative | 105.65 | 29.21 | 3.62 | **0** |
|  |  | Negative 2nd order | -56.22 | 17.55 | -3.20 | **0** |
|  | Calidris (Genus) | Positive | -7.92 | 4.18 | -1.90 | 0.06 |
|  |  | Positive 2nd order | 14.39 | 4.56 | 3.15 | **0** |
|  |  | Negative | -0.91 | 5.23 | -0.17 | 0.86 |
|  |  | Negative 2nd order | 2.04 | 5.68 | 0.36 | 0.72 |
|  | Limosa (Genus) | Positive | -0.01 | 0.14 | -0.07 | 0.95 |
|  | Numenius (Genus) | Positive | -2.43 | 2.35 | -1.04 | 0.3 |
|  |  | Positive 2nd order | 4.61 | 2.52 | 1.83 | 0.07 |
|  | Philomachus (Genus) | Positive | 0.00 | 0.19 | 0.00 | 1 |
|  | Tringa (Genus) | Positive | -0.10 | 0.01 | -10.78 | **0** |
|  |  | Negative | 0.03 | 0.01 | 3.13 | **0** |
|  | Ciconiiformes (Order) | Positive | 0.00 | 0.15 | 0.00 | 1 |
|  |  | Negative | 0.02 | 0.15 | 0.13 | 0.9 |
|  | Threskiornithidae (Family) | Positive | -0.01 | 0.14 | -0.07 | 0.95 |
|  | Platalea (Genus) | Positive | -0.01 | 0.14 | -0.07 | 0.95 |
|  | Pelecaniformes (Order) | Negative | 0.01 | 0.14 | 0.07 | 0.95 |
|  | Phalacrocoracidae (Family) | Negative | 0.01 | 0.14 | 0.07 | 0.95 |
|  | Phalacrocorax (Genus) | Negative | 0.01 | 0.14 | 0.07 | 0.95 |
|  | Teleostei (Class) | Positive | -4.17 | 2.73 | -1.53 | 0.13 |
|  |  | Positive 2nd order | 14.95 | 2.75 | 5.43 | **0** |
|  |  | Negative | -2.85 | 2.94 | -0.97 | 0.33 |
|  |  | Negative 2nd order | 9.11 | 2.96 | 3.07 | **0** |
|  | Anguilliformes (Order) | Negative | 0.03 | 0.17 | 0.16 | 0.87 |
|  | Anguillidae (Family) | Negative | 0.03 | 0.17 | 0.16 | 0.87 |
|  | Anguilla (Genus) | Negative | 0.03 | 0.17 | 0.16 | 0.87 |
|  | Beloniformes (Order) | Negative | 0.02 | 0.36 | 0.06 | 0.95 |
|  | Belonidae (Family) | Negative | 0.02 | 0.36 | 0.06 | 0.95 |
|  | Belone (Genus) | Negative | 0.02 | 0.36 | 0.06 | 0.95 |
|  | Carangiformes (Order) | Positive | -0.01 | 0.15 | -0.10 | 0.92 |
|  | Carangidae (Family) | Positive | -0.01 | 0.15 | -0.10 | 0.92 |
|  | Trachurus (Genus) | Positive | -0.01 | 0.15 | -0.10 | 0.92 |
|  | Clupeiformes (Order) | Positive | 1.37 | 2.15 | 0.64 | 0.52 |
|  |  | Positive 2nd order | 1.86 | 2.15 | 0.86 | 0.39 |
|  |  | Negative | 16.30 | 5.93 | 2.75 | **0.01** |
|  |  | Negative 2nd order | -20.00 | 6.24 | -3.20 | **0** |
|  | Alosidae (Family) | Negative | -0.08 | 0.24 | -0.32 | 0.75 |
|  | Alosa (Genus) | Negative | -0.08 | 0.24 | -0.32 | 0.75 |
|  | Clupeidae (Family) | Positive | 2.29 | 2.09 | 1.09 | 0.27 |
|  |  | Positive 2nd order | 1.40 | 2.10 | 0.67 | 0.51 |
|  |  | Negative | 63.58 | 16.61 | 3.83 | **0** |
|  |  | Negative 2nd order | -54.97 | 13.61 | -4.04 | **0** |
|  | Clupea (Genus) | Positive | 0.01 | 0.07 | 0.12 | 0.9 |
|  |  | Negative | 0.04 | 0.11 | 0.38 | 0.7 |
|  | Sprattus (Genus) | Positive | 0.00 | 0.08 | 0.04 | 0.97 |
|  |  | Negative | 0.05 | 0.10 | 0.44 | 0.66 |
|  | Gadiformes (Order) | Positive | 1.62 | 2.94 | 0.55 | 0.58 |
|  |  | Positive 2nd order | 9.61 | 2.96 | 3.24 | **0** |
|  |  | Negative | -0.50 | 2.67 | -0.19 | 0.85 |
|  |  | Negative 2nd order | 12.98 | 2.73 | 4.76 | **0** |
|  | Gadidae (Family) | Positive | 2.15 | 3.01 | 0.71 | 0.48 |
|  |  | Positive 2nd order | 8.95 | 3.03 | 2.96 | **0** |
|  |  | Negative | -3.82 | 2.85 | -1.34 | 0.18 |
|  |  | Negative 2nd order | 14.21 | 2.91 | 4.88 | **0** |
|  | Gadus (Genus) | Negative | 0.03 | 0.13 | 0.24 | 0.81 |
|  | Merlangius (Genus) | Positive | 0.01 | 0.09 | 0.16 | 0.88 |
|  |  | Negative | -0.02 | 0.08 | -0.24 | 0.81 |
|  | Trisopterus (Genus) | Positive | 0.00 | 0.09 | 0.04 | 0.97 |
|  |  | Negative | 0.00 | 0.10 | 0.04 | 0.97 |
|  | Lotidae (Family) | Positive | 0.00 | 0.09 | -0.02 | 0.99 |
|  |  | Negative | 0.03 | 0.09 | 0.34 | 0.74 |
|  | Ciliata (Genus) | Positive | 0.00 | 0.09 | -0.02 | 0.99 |
|  |  | Negative | 0.03 | 0.09 | 0.34 | 0.74 |
|  | Gobiiformes (Order) | Positive | 0.01 | 0.02 | 0.49 | 0.62 |
|  |  | Negative | 0.03 | 0.01 | 2.36 | **0.02** |
|  | Gobiidae (Family) | Positive | 0.01 | 0.02 | 0.49 | 0.62 |
|  |  | Negative | 0.03 | 0.01 | 2.36 | **0.02** |
|  | Pomatoschistus (Genus) | Positive | 0.01 | 0.02 | 0.47 | 0.64 |
|  |  | Negative | 0.03 | 0.01 | 2.35 | **0.02** |
|  | Osmeriformes (Order) | Positive | -0.01 | 0.06 | -0.20 | 0.84 |
|  |  | Negative | 0.01 | 0.07 | 0.11 | 0.91 |
|  | Osmeridae (Family) | Positive | -0.01 | 0.06 | -0.20 | 0.84 |
|  |  | Negative | 0.01 | 0.07 | 0.11 | 0.91 |
|  | Osmerus (Genus) | Positive | -0.01 | 0.06 | -0.20 | 0.84 |
|  |  | Negative | 0.01 | 0.07 | 0.11 | 0.91 |
|  | Perciformes (Order) | Positive | -5.27 | 2.94 | -1.79 | 0.07 |
|  |  | Positive 2nd order | 8.49 | 2.95 | 2.88 | **0** |
|  |  | Negative | -7.18 | 3.45 | -2.08 | **0.04** |
|  |  | Negative 2nd order | 9.58 | 3.46 | 2.77 | **0.01** |
|  | Agonidae (Family) | Negative | -0.04 | 0.13 | -0.31 | 0.76 |
|  | Agonus (Genus) | Negative | -0.04 | 0.13 | -0.31 | 0.76 |
|  | Ammodytidae (Family) | Positive | 0.00 | 0.02 | 0.00 | 1 |
|  |  | Negative | 0.00 | 0.02 | 0.00 | 1 |
|  | Ammodytes (Genus) | Positive | 0.00 | 0.09 | 0.00 | 1 |
|  |  | Negative | 0.00 | 0.11 | 0.00 | 1 |
|  | Cottidae (Family) | Positive | -0.01 | 0.10 | -0.10 | 0.92 |
|  |  | Negative | -0.01 | 0.09 | -0.07 | 0.95 |
|  | Myoxocephalus (Genus) | Positive | -0.01 | 0.10 | -0.10 | 0.92 |
|  |  | Negative | -0.01 | 0.09 | -0.07 | 0.95 |
|  | Liparidae (Family) | Negative | 0.13 | 0.33 | 0.40 | 0.69 |
|  | Liparis (Genus) | Negative | 0.13 | 0.33 | 0.40 | 0.69 |
|  | Pholidae (Family) | Positive | 0.00 | 0.09 | 0.04 | 0.97 |
|  | Pholis (Genus) | Positive | 0.00 | 0.09 | 0.04 | 0.97 |
|  | Triglidae (Family) | Positive | -0.01 | 0.15 | -0.10 | 0.92 |
|  | Chelidonichthys (Genus) | Positive | -0.01 | 0.15 | -0.10 | 0.92 |
|  | Zoarcidae (Family) | Positive | -0.05 | 0.12 | -0.39 | 0.7 |
|  |  | Negative | -0.04 | 0.12 | -0.37 | 0.71 |
|  | Zoarces (Genus) | Positive | -0.05 | 0.12 | -0.39 | 0.7 |
|  |  | Negative | -0.04 | 0.12 | -0.37 | 0.71 |
|  | Pleuronectiformes (Order) | Positive | -4.12 | 2.58 | -1.60 | 0.11 |
|  |  | Positive 2nd order | 9.12 | 2.60 | 3.51 | **0** |
|  |  | Negative | -6.85 | 2.76 | -2.48 | **0.01** |
|  |  | Negative 2nd order | 2.61 | 2.77 | 0.94 | 0.35 |
|  | Pleuronectidae (Family) | Positive | -7.36 | 2.55 | -2.88 | **0** |
|  |  | Positive 2nd order | 10.71 | 2.56 | 4.18 | **0** |
|  |  | Negative | -9.67 | 2.83 | -3.42 | **0** |
|  |  | Negative 2nd order | 5.60 | 2.82 | 1.98 | 0.05 |
|  | Limanda (Genus) | Positive | -0.01 | 0.08 | -0.11 | 0.91 |
|  |  | Negative | -0.02 | 0.08 | -0.21 | 0.83 |
|  | Platichthys (Genus) | Positive | -0.01 | 0.07 | -0.08 | 0.94 |
|  |  | Negative | 0.00 | 0.08 | -0.02 | 0.99 |
|  | Pleuronectes (Genus) | Positive | -0.02 | 0.06 | -0.41 | 0.68 |
|  |  | Negative | -0.03 | 0.06 | -0.53 | 0.6 |
|  | Scophthalmidae (Family) | Positive | -0.03 | 0.23 | -0.12 | 0.91 |
|  | Scophthalmus (Genus) | Positive | -0.03 | 0.23 | -0.12 | 0.91 |
|  | Soleidae (Family) | Positive | 0.03 | 0.08 | 0.33 | 0.74 |
|  |  | Negative | 0.01 | 0.08 | 0.11 | 0.91 |
|  | Solea (Genus) | Positive | 0.03 | 0.08 | 0.33 | 0.74 |
|  |  | Negative | 0.01 | 0.08 | 0.11 | 0.91 |
|  | Syngnathiformes (Order) | Positive | -0.02 | 0.02 | -1.02 | 0.31 |
|  |  | Negative | -0.03 | 0.02 | -1.91 | 0.06 |
|  | Syngnathidae (Family) | Positive | -0.02 | 0.02 | -1.02 | 0.31 |
|  |  | Negative | -0.03 | 0.02 | -1.91 | 0.06 |
|  | Syngnathus (Genus) | Positive | -0.02 | 0.02 | -1.02 | 0.31 |
|  |  | Negative | -0.03 | 0.02 | -1.91 | 0.06 |
| Ciliophora | Ciliophora (Phylum) | Negative | 0.00 | 0.15 | 0.00 | 1 |
|  | Litostomatea (Class) | Negative | 0.00 | 0.46 | 0.00 | 1 |
|  | Cyclotrichiida (Order) | Negative | 0.00 | 0.46 | 0.00 | 1 |
|  | Mesodiniidae (Family) | Negative | 0.00 | 0.46 | 0.00 | 1 |
|  | Mesodinium (Genus) | Negative | 0.00 | 0.46 | 0.00 | 1 |
|  | Oligotrichea (Class) | Negative | 0.00 | 0.56 | 0.00 | 1 |
|  | Oligotrichida (Order) | Negative | 0.00 | 0.56 | 0.00 | 1 |
|  | Tontoniidae (Family) | Negative | 0.00 | 0.56 | 0.00 | 1 |
|  | Laboea (Genus) | Negative | 0.00 | 0.56 | 0.00 | 1 |
| Cryptista | Cryptista (Phylum) | Negative | 0.00 | 0.12 | 0.00 | 1 |
|  | Katablepharidophyceae (Class) | Negative | 0.00 | 0.12 | 0.00 | 1 |
|  | Katablepharidales (Order) | Negative | 0.00 | 0.12 | 0.00 | 1 |
|  | Katablepharidaceae (Family) | Negative | 0.00 | 0.12 | 0.00 | 1 |
|  | Katablepharis (Genus) | Negative | 0.00 | 0.44 | 0.00 | 1 |
|  | Leucocryptos (Genus) | Negative | 0.00 | 0.43 | 0.00 | 1 |
| Cryptophyta | Cryptophyta (Phylum) | Positive | 0.04 | 0.05 | 0.87 | 0.39 |
|  |  | Negative | 0.00 | 0.07 | 0.04 | 0.97 |
|  | Cryptophyceae (Class) | Positive | 0.04 | 0.05 | 0.89 | 0.38 |
|  |  | Negative | 0.00 | 0.08 | 0.03 | 0.97 |
|  | Cryptomonadales (Order) | Positive | 0.03 | 0.22 | 0.14 | 0.89 |
|  | Pyrenomonadales (Order) | Negative | 0.00 | 0.08 | 0.00 | 1 |
|  | Chroomonadacea (Family) | Negative | 0.00 | 0.44 | 0.00 | 1 |
|  | Hemiselmis (Genus) | Negative | 0.00 | 0.44 | 0.00 | 1 |
|  | Geminigeraceae (Family) | Negative | 0.00 | 0.13 | 0.00 | 1 |
|  | Plagioselmis (Genus) | Negative | 0.00 | 0.46 | 0.00 | 1 |
|  | Teleaulax (Genus) | Negative | 0.00 | 0.17 | 0.00 | 1 |
|  | Hemiselmidaceae (Family) | Negative | 0.00 | 0.20 | 0.00 | 1 |
|  | Chroomonas (Genus) | Negative | 0.00 | 0.20 | 0.00 | 1 |
|  | Pyrenomonadaceae (Family) | Negative | 0.00 | 0.46 | 0.00 | 1 |
|  | Rhodomonas (Genus) | Negative | 0.00 | 0.46 | 0.00 | 1 |
|  | Telonemea (Class) | Negative | 0.00 | 0.44 | 0.00 | 1 |
|  | Telonemida (Order) | Negative | 0.00 | 0.44 | 0.00 | 1 |
|  | Telonemidae (Family) | Negative | 0.00 | 0.44 | 0.00 | 1 |
|  | Telonema (Genus) | Negative | 0.00 | 0.44 | 0.00 | 1 |
| Dinoflagellata | Dinoflagellata (Phylum) | Positive | -1.85 | 4.43 | -0.42 | 0.68 |
|  |  | Positive 2nd order | 4.26 | 4.60 | 0.93 | 0.35 |
|  |  | Negative | 2.62 | 4.05 | 0.65 | 0.52 |
|  |  | Negative 2nd order | -5.07 | 4.00 | -1.27 | 0.2 |
|  | Dinophyceae (Class) | Positive | -1.85 | 4.43 | -0.42 | 0.68 |
|  |  | Positive 2nd order | 4.26 | 4.60 | 0.93 | 0.35 |
|  |  | Negative | 2.62 | 4.05 | 0.65 | 0.52 |
|  |  | Negative 2nd order | -5.07 | 4.00 | -1.27 | 0.2 |
|  | Gymnodiniales (Order) | Positive | -8.91 | 4.38 | -2.03 | **0.04** |
|  |  | Positive 2nd order | 7.22 | 4.57 | 1.58 | 0.11 |
|  |  | Negative | 2.76 | 3.35 | 0.82 | 0.41 |
|  |  | Negative 2nd order | -4.12 | 3.30 | -1.25 | 0.21 |
|  | Gymnodiniaceae (Family) | Positive | -7.33 | 3.71 | -1.97 | 0.05 |
|  |  | Positive 2nd order | 6.28 | 3.90 | 1.61 | 0.11 |
|  |  | Negative | 54.52 | 23.33 | 2.34 | **0.02** |
|  |  | Negative 2nd order | -45.07 | 18.37 | -2.45 | **0.01** |
|  | Gymnodinium (Genus) | Negative | 0.00 | 0.25 | 0.00 | 1 |
|  | Gyrodinium (Genus) | Positive | -6.94 | 3.52 | -1.97 | 0.05 |
|  |  | Positive 2nd order | 5.65 | 3.65 | 1.55 | 0.12 |
|  |  | Negative | 58.06 | 25.28 | 2.30 | **0.02** |
|  |  | Negative 2nd order | -46.16 | 19.15 | -2.41 | **0.02** |
|  | Warnowiaceae (Family) | Negative | 0.00 | 0.87 | 0.00 | 1 |
|  | Nematopsides (Genus) | Negative | 0.00 | 0.87 | 0.00 | 1 |
|  | Lebouridinium (Genus) | Negative | 0.00 | 0.22 | -0.01 | 1 |
|  | Noctilucales (Order) | Negative | 0.00 | 0.73 | 0.00 | 1 |
|  | Noctilucaceae (Family) | Negative | 0.00 | 0.73 | 0.00 | 1 |
|  | Noctiluca (Genus) | Negative | 0.00 | 0.73 | 0.00 | 1 |
|  | Peridiniales (Order) | Positive | 4.82 | 3.79 | 1.27 | 0.2 |
|  |  | Positive 2nd order | -2.62 | 3.95 | -0.66 | 0.51 |
|  |  | Negative | 55.51 | 27.26 | 2.04 | **0.04** |
|  |  | Negative 2nd order | -49.69 | 23.15 | -2.15 | **0.03** |
|  | Heterocapsaceae (Family) | Positive | 0.05 | 0.04 | 1.32 | 0.19 |
|  |  | Negative | 0.04 | 0.21 | 0.17 | 0.86 |
|  | Heterocapsa (Genus) | Positive | 0.05 | 0.04 | 1.32 | 0.19 |
|  |  | Negative | 0.04 | 0.21 | 0.17 | 0.86 |
|  | Kryptoperidiniaceae (Family) | Negative | 0.00 | 0.73 | 0.00 | 1 |
|  | Kryptoperidinium (Genus) | Negative | 0.00 | 0.73 | 0.00 | 1 |
|  | Protoperidiniaceae (Family) | Negative | 53.17 | 26.82 | 1.98 | 0.05 |
|  |  | Negative 2nd order | -48.26 | 22.85 | -2.11 | **0.03** |
|  | Diplopsalis (Genus) | Negative | 0.00 | 0.61 | 0.00 | 1 |
|  | Preperidinium (Genus) | Negative | 0.00 | 0.87 | 0.00 | 1 |
|  | Protoperidinium (Genus) | Negative | 67.32 | 34.29 | 1.96 | 0.05 |
|  |  | Negative 2nd order | -58.59 | 27.86 | -2.10 | **0.04** |
|  | Peridiniella (Genus) | Negative | 0.00 | 0.56 | 0.00 | 1 |
|  | Prorocentrales (Order) | Negative | -0.09 | 0.05 | -1.73 | 0.08 |
|  | Prorocentraceae (Family) | Negative | -0.09 | 0.05 | -1.73 | 0.08 |
|  | Prorocentrum (Genus) | Negative | -0.09 | 0.05 | -1.73 | 0.08 |
|  | Thoracosphaerales (Order) | Negative | 0.00 | 0.46 | 0.00 | 1 |
|  | Thoracosphaeraceae (Family) | Negative | 0.00 | 0.46 | 0.00 | 1 |
|  | Scrippsiella (Genus) | Negative | 0.00 | 0.46 | 0.00 | 1 |
| Echinodermata | Echinodermata (Phylum) | Positive | -0.04 | 0.23 | -0.19 | 0.85 |
| Euglenophyta | Euglenophyta (Phylum) | Positive | -4.01 | 2.90 | -1.38 | 0.17 |
|  |  | Positive 2nd order | 2.47 | 2.97 | 0.83 | 0.41 |
|  |  | Negative | 54.58 | 19.54 | 2.79 | **0.01** |
|  |  | Negative 2nd order | -48.25 | 16.19 | -2.98 | **0** |
|  | Euglenophyceae (Class) | Positive | -4.01 | 2.90 | -1.38 | 0.17 |
|  |  | Positive 2nd order | 2.47 | 2.97 | 0.83 | 0.41 |
|  |  | Negative | 54.58 | 19.54 | 2.79 | **0.01** |
|  |  | Negative 2nd order | -48.25 | 16.19 | -2.98 | **0** |
|  | Eutreptiales (Order) | Positive | -4.01 | 2.90 | -1.38 | 0.17 |
|  |  | Positive 2nd order | 2.47 | 2.97 | 0.83 | 0.41 |
|  |  | Negative | 54.58 | 19.54 | 2.79 | **0.01** |
|  |  | Negative 2nd order | -48.25 | 16.19 | -2.98 | **0** |
|  | Eutreptiaceae (Family) | Positive | -4.01 | 2.90 | -1.38 | 0.17 |
|  |  | Positive 2nd order | 2.47 | 2.97 | 0.83 | 0.41 |
|  |  | Negative | 54.58 | 19.54 | 2.79 | **0.01** |
|  |  | Negative 2nd order | -48.25 | 16.19 | -2.98 | **0** |
|  | Eutreptiella (Genus) | Positive | -4.01 | 2.90 | -1.38 | 0.17 |
|  |  | Positive 2nd order | 2.47 | 2.97 | 0.83 | 0.41 |
|  |  | Negative | 54.58 | 19.54 | 2.79 | **0.01** |
|  |  | Negative 2nd order | -48.25 | 16.19 | -2.98 | **0** |
| Haptophyta | Haptophyta (Phylum) | Positive | -0.07 | 0.05 | -1.31 | 0.19 |
|  |  | Negative | 0.01 | 0.16 | 0.07 | 0.94 |
|  | Coccolithophyceae (Class) | Positive | -0.07 | 0.05 | -1.31 | 0.19 |
|  |  | Negative | 0.01 | 0.16 | 0.07 | 0.94 |
|  | Phaeocystales (Order) | Positive | -0.07 | 0.05 | -1.31 | 0.19 |
|  |  | Negative | 0.01 | 0.16 | 0.07 | 0.94 |
|  | Phaeocystaceae (Family) | Positive | -0.07 | 0.05 | -1.31 | 0.19 |
|  |  | Negative | 0.01 | 0.16 | 0.07 | 0.94 |
|  | Phaeocystis (Genus) | Positive | -0.07 | 0.05 | -1.31 | 0.19 |
|  |  | Negative | 0.01 | 0.16 | 0.07 | 0.94 |
| Heterokontophyta | Heterokontophyta (Phylum) | Positive | -12.92 | 3.44 | -3.76 | **0** |
|  |  | Positive 2nd order | 11.01 | 3.52 | 3.12 | **0** |
|  |  | Negative | -5.27 | 2.91 | -1.81 | 0.07 |
|  |  | Negative 2nd order | 2.09 | 2.89 | 0.72 | 0.47 |
|  | Bacillariophyceae (Class) | Positive | -0.02 | 0.03 | -0.83 | 0.41 |
|  |  | Negative | -0.03 | 0.02 | -1.44 | 0.15 |
|  | Bacillariales (Order) | Positive | -0.05 | 0.06 | -0.88 | 0.38 |
|  |  | Negative | 0.01 | 0.04 | 0.14 | 0.89 |
|  | Bacillariaceae (Family) | Negative | 20.43 | 8.56 | 2.39 | **0.02** |
|  |  | Negative 2nd order | -22.71 | 8.35 | -2.72 | **0.01** |
|  | Cylindrotheca (Genus) | Negative | 0.00 | 0.30 | 0.00 | 1 |
|  | Pseudo-nitzschia (Genus) | Negative | 52.55 | 21.13 | 2.49 | **0.01** |
|  |  | Negative 2nd order | -54.31 | 20.07 | -2.71 | **0.01** |
|  | Naviculales (Order) | Positive | -0.01 | 0.10 | -0.05 | 0.96 |
|  |  | Negative | 0.02 | 0.14 | 0.18 | 0.86 |
|  | Naviculaceae (Family) | Negative | 0.04 | 0.13 | 0.28 | 0.78 |
|  | Gyrosigma (Genus) | Negative | 0.03 | 0.17 | 0.17 | 0.86 |
|  | Navicula (Genus) | Negative | 0.00 | 0.61 | 0.00 | 1 |
|  | Pleurosigmataceae (Family) | Positive | 0.00 | 0.47 | 0.00 | 1 |
|  | Pleurosigma (Genus) | Positive | 0.00 | 0.47 | 0.00 | 1 |
|  | Rhaphoneidales (Order) | Positive | 0.09 | 0.08 | 1.19 | 0.23 |
|  |  | Negative | -0.08 | 0.04 | -1.94 | 0.05 |
|  | Asterionellopsidaceae (Family) | Positive | 0.09 | 0.08 | 1.19 | 0.23 |
|  |  | Negative | -0.08 | 0.04 | -1.94 | 0.05 |
|  | Asterionellopsis (Genus) | Positive | 0.05 | 0.24 | 0.21 | 0.84 |
|  | Asteroplanus (Genus) | Negative | -0.05 | 0.18 | -0.27 | 0.78 |
|  | Rhaponeidales (Order) | Positive | 10.71 | 7.16 | 1.49 | 0.13 |
|  |  | Positive 2nd order | -7.36 | 5.96 | -1.23 | 0.22 |
|  | Rhaphoneidaceae (Family) | Positive | 10.71 | 7.16 | 1.49 | 0.13 |
|  |  | Positive 2nd order | -7.36 | 5.96 | -1.23 | 0.22 |
|  | Delphineis (Genus) | Positive | 0.07 | 0.24 | 0.28 | 0.78 |
|  | Thalassionematales (Order) | Positive | -0.08 | 0.19 | -0.45 | 0.65 |
|  |  | Negative | -0.04 | 0.17 | -0.26 | 0.8 |
|  | Thalassionemataceae (Family) | Positive | -0.08 | 0.19 | -0.45 | 0.65 |
|  |  | Negative | -0.04 | 0.17 | -0.26 | 0.8 |
|  | Thalassionema (Genus) | Positive | -0.08 | 0.19 | -0.45 | 0.65 |
|  |  | Negative | -0.04 | 0.17 | -0.26 | 0.8 |
|  | Triceratiales (Order) | Negative | 0.00 | 0.46 | 0.00 | 1 |
|  | Triceratiaceae (Family) | Negative | 0.00 | 0.46 | 0.00 | 1 |
|  | Ralfsiella (Genus) | Negative | 0.00 | 0.46 | 0.00 | 1 |
|  | Mediopyxis (Genus) | Negative | 0.00 | 0.29 | 0.00 | 1 |
|  | Chrysophyceae (Class) | Negative | 0.00 | 0.28 | 0.00 | 1 |
|  | Chromulinales (Order) | Negative | 0.00 | 0.28 | 0.00 | 1 |
|  | Dinobryaceae (Family) | Negative | 0.00 | 0.28 | 0.00 | 1 |
|  | Dinobryon (Genus) | Negative | 0.00 | 0.28 | 0.00 | 1 |
|  | Coscinodiscophyceae (Class) | Positive | -0.02 | 0.03 | -0.77 | 0.44 |
|  |  | Negative | -0.01 | 0.02 | -0.39 | 0.7 |
|  | Coscinodiscales (Order) | Positive | 51.44 | 38.21 | 1.35 | 0.18 |
|  |  | Positive 2nd order | -49.44 | 34.31 | -1.44 | 0.15 |
|  |  | Negative | 24.11 | 8.43 | 2.86 | **0** |
|  |  | Negative 2nd order | -22.86 | 7.36 | -3.10 | **0** |
|  | Heliopeltaceae (Family) | Positive | 52.84 | 38.72 | 1.36 | 0.17 |
|  |  | Positive 2nd order | -47.83 | 32.95 | -1.45 | 0.15 |
|  |  | Negative | 25.04 | 8.53 | 2.94 | **0** |
|  |  | Negative 2nd order | -22.39 | 7.06 | -3.17 | **0** |
|  | Actinoptychus (Genus) | Positive | 52.84 | 38.72 | 1.36 | 0.17 |
|  |  | Positive 2nd order | -47.83 | 32.95 | -1.45 | 0.15 |
|  |  | Negative | 25.04 | 8.53 | 2.94 | **0** |
|  |  | Negative 2nd order | -22.39 | 7.06 | -3.17 | **0** |
|  | Melosirales (Order) | Negative | -0.09 | 0.38 | -0.23 | 0.82 |
|  | Paraliales (Order) | Negative | -0.04 | 0.15 | -0.28 | 0.78 |
|  | Paraliaceae (Family) | Negative | -0.04 | 0.15 | -0.28 | 0.78 |
|  | Paralia (Genus) | Negative | -0.04 | 0.15 | -0.28 | 0.78 |
|  | Rhizosoleniales (Order) | Positive | -0.03 | 0.03 | -1.01 | 0.31 |
|  |  | Negative | 0.00 | 0.02 | -0.22 | 0.83 |
|  | Rhizosoleniaceae (Family) | Positive | -0.03 | 0.03 | -1.01 | 0.31 |
|  |  | Negative | 0.00 | 0.02 | -0.22 | 0.83 |
|  | Dactyliosolen (Genus) | Negative | 0.08 | 0.40 | 0.19 | 0.85 |
|  | Guinardia (Genus) | Negative | 80.77 | 20.02 | 4.03 | **0** |
|  |  | Negative 2nd order | -64.22 | 14.98 | -4.29 | **0** |
|  | Rhizosolenia (Genus) | Positive | -0.08 | 0.19 | -0.41 | 0.68 |
|  |  | Negative | -0.03 | 0.18 | -0.16 | 0.87 |
|  | Sundstroemia (Genus) | Positive | -2.15 | 2.64 | -0.82 | 0.42 |
|  |  | Positive 2nd order | 4.23 | 2.64 | 1.60 | 0.11 |
|  |  | Negative | -6.46 | 2.58 | -2.50 | **0.01** |
|  |  | Negative 2nd order | 5.20 | 2.66 | 1.96 | 0.05 |
|  | Dictyochophyceae (Class) | Negative | 0.00 | 0.17 | 0.00 | 1 |
|  | Pedinellales (Order) | Negative | 0.00 | 0.17 | 0.00 | 1 |
|  | Actinomonadaceae (Family) | Negative | 0.00 | 0.17 | 0.00 | 1 |
|  | Pseudopedinella (Genus) | Negative | 0.00 | 0.17 | 0.00 | 1 |
|  | Mediophyceae (Class) | Positive | -11.86 | 3.12 | -3.80 | **0** |
|  |  | Positive 2nd order | 9.00 | 3.21 | 2.81 | **0.01** |
|  |  | Negative | -3.83 | 3.07 | -1.25 | 0.21 |
|  |  | Negative 2nd order | 2.29 | 3.04 | 0.75 | 0.45 |
|  | Anaulales (Order) | Positive | -0.06 | 0.16 | -0.41 | 0.68 |
|  |  | Negative | 0.04 | 0.19 | 0.21 | 0.84 |
|  | Anaulaceae (Family) | Positive | -0.06 | 0.16 | -0.41 | 0.68 |
|  |  | Negative | 0.04 | 0.19 | 0.21 | 0.84 |
|  | Eunotogramma (Genus) | Positive | -0.06 | 0.16 | -0.41 | 0.68 |
|  |  | Negative | 0.04 | 0.19 | 0.21 | 0.84 |
|  | Biddulphiales (Order) | Negative | 0.08 | 0.31 | 0.24 | 0.81 |
|  | Biddulphiaceae (Family) | Negative | 0.08 | 0.31 | 0.24 | 0.81 |
|  | Neobrightwellia (Genus) | Negative | 0.08 | 0.31 | 0.24 | 0.81 |
|  | Chaetocerotales (Order) | Positive | 0.00 | 0.03 | -0.03 | 0.98 |
|  |  | Negative | 0.03 | 0.03 | 1.03 | 0.3 |
|  | Chaetocerotaceae (Family) | Positive | 14.65 | 6.45 | 2.27 | **0.02** |
|  |  | Positive 2nd order | -9.43 | 5.82 | -1.62 | 0.11 |
|  |  | Negative | -0.65 | 3.47 | -0.19 | 0.85 |
|  |  | Negative 2nd order | -0.85 | 3.37 | -0.25 | 0.8 |
|  | Chaetoceros (Genus) | Positive | 0.10 | 0.05 | 2.14 | **0.03** |
|  |  | Negative | -0.01 | 0.04 | -0.19 | 0.85 |
|  | Leptocylindraceae (Family) | Positive | -7.93 | 3.71 | -2.14 | **0.03** |
|  |  | Positive 2nd order | 4.14 | 3.88 | 1.07 | 0.29 |
|  |  | Negative | 76.31 | 28.54 | 2.67 | **0.01** |
|  |  | Negative 2nd order | -64.01 | 22.67 | -2.82 | **0** |
|  | Leptocylindrus (Genus) | Negative | 64.60 | 23.95 | 2.70 | **0.01** |
|  |  | Negative 2nd order | -53.52 | 18.84 | -2.84 | **0** |
|  | Cymatosirales (Order) | Positive | 2.46 | 3.44 | 0.71 | 0.48 |
|  |  | Positive 2nd order | -0.97 | 3.63 | -0.27 | 0.79 |
|  |  | Negative | -9.68 | 3.29 | -2.94 | **0** |
|  |  | Negative 2nd order | 7.90 | 3.15 | 2.51 | **0.01** |
|  | Cymatosiraceae (Family) | Positive | 2.46 | 3.44 | 0.71 | 0.48 |
|  |  | Positive 2nd order | -0.97 | 3.63 | -0.27 | 0.79 |
|  |  | Negative | -9.68 | 3.29 | -2.94 | **0** |
|  |  | Negative 2nd order | 7.90 | 3.15 | 2.51 | **0.01** |
|  | Brockmanniella (Genus) | Negative | -0.09 | 0.20 | -0.45 | 0.65 |
|  | Plagiogrammopsis (Genus) | Positive | 0.03 | 0.04 | 0.70 | 0.48 |
|  |  | Negative | -0.06 | 0.05 | -1.34 | 0.18 |
|  | Eupodiscales (Order) | Positive | -0.05 | 0.02 | -2.22 | **0.03** |
|  |  | Negative | -0.02 | 0.03 | -0.79 | 0.43 |
|  | Eupodiscaceae (Family) | Positive | -8.67 | 4.01 | -2.16 | **0.03** |
|  |  | Positive 2nd order | 7.57 | 4.16 | 1.82 | 0.07 |
|  |  | Negative | 2.64 | 28.90 | 0.09 | 0.93 |
|  |  | Negative 2nd order | -2.89 | 29.73 | -0.10 | 0.92 |
|  | Zygoceros (Genus) | Positive | -8.31 | 3.89 | -2.14 | **0.03** |
|  |  | Positive 2nd order | 6.99 | 3.96 | 1.76 | 0.08 |
|  |  | Negative | 7.51 | 25.68 | 0.29 | 0.77 |
|  |  | Negative 2nd order | -8.04 | 25.47 | -0.32 | 0.75 |
|  | Odontellaceae (Family) | Positive | -0.01 | 0.14 | -0.04 | 0.97 |
|  |  | Negative | 0.08 | 0.20 | 0.39 | 0.7 |
|  | Odontella (Genus) | Positive | -0.01 | 0.14 | -0.04 | 0.97 |
|  |  | Negative | 0.08 | 0.20 | 0.39 | 0.7 |
|  | Parodontellaceae (Family) | Positive | -0.08 | 0.04 | -2.13 | **0.03** |
|  |  | Negative | -0.06 | 0.03 | -1.68 | 0.09 |
|  | Trieres (Genus) | Positive | -0.08 | 0.04 | -2.13 | **0.03** |
|  |  | Negative | -0.06 | 0.03 | -1.68 | 0.09 |
|  | Lithodesmiales (Order) | Positive | -0.04 | 0.03 | -1.28 | 0.2 |
|  |  | Negative | -0.01 | 0.03 | -0.20 | 0.84 |
|  | Lithodesmiaceae (Family) | Positive | -0.04 | 0.03 | -1.28 | 0.2 |
|  |  | Negative | -0.01 | 0.03 | -0.20 | 0.84 |
|  | Ditylum (Genus) | Positive | 0.06 | 0.23 | 0.25 | 0.81 |
|  |  | Negative | -0.04 | 0.17 | -0.22 | 0.83 |
|  | Lithodesmium (Genus) | Positive | -0.05 | 0.19 | -0.29 | 0.77 |
|  |  | Negative | 0.05 | 0.22 | 0.23 | 0.82 |
|  | Thalassiosirales (Order) | Positive | -12.48 | 3.74 | -3.34 | **0** |
|  |  | Positive 2nd order | 11.21 | 3.81 | 2.95 | **0** |
|  |  | Negative | -5.93 | 2.94 | -2.02 | **0.04** |
|  |  | Negative 2nd order | 6.52 | 2.89 | 2.26 | **0.02** |
|  | Skeletonemaceae (Family) | Negative | 0.03 | 0.16 | 0.20 | 0.84 |
|  | Skeletonema (Genus) | Negative | 0.03 | 0.16 | 0.20 | 0.84 |
|  | Thalassiosiraceae (Family) | Positive | -11.12 | 3.25 | -3.42 | **0** |
|  |  | Positive 2nd order | 9.91 | 3.31 | 3.00 | **0** |
|  |  | Negative | -5.57 | 2.59 | -2.15 | **0.03** |
|  |  | Negative 2nd order | 6.03 | 2.54 | 2.37 | **0.02** |
|  | Detonula (Genus) | Positive | -0.12 | 0.37 | -0.33 | 0.74 |
|  |  | Negative | 0.00 | 0.48 | 0.00 | 1 |
|  | Thalassiosira (Genus) | Positive | -8.67 | 3.23 | -2.68 | **0.01** |
|  |  | Positive 2nd order | 7.72 | 3.30 | 2.34 | **0.02** |
|  |  | Negative | -5.33 | 2.47 | -2.16 | **0.03** |
|  |  | Negative 2nd order | 5.98 | 2.45 | 2.44 | **0.01** |
| Mollusca | Mollusca (Phylum) | Positive | -4.32 | 3.19 | -1.36 | 0.18 |
|  |  | Positive 2nd order | 1.21 | 3.15 | 0.38 | 0.7 |
|  |  | Negative | -2.47 | 2.60 | -0.95 | 0.34 |
|  |  | Negative 2nd order | 7.48 | 2.58 | 2.90 | **0** |
|  | Bivalvia (Class) | Positive | -5.07 | 3.12 | -1.63 | 0.1 |
|  |  | Positive 2nd order | 3.93 | 3.08 | 1.28 | 0.2 |
|  |  | Negative | -4.92 | 2.69 | -1.83 | 0.07 |
|  |  | Negative 2nd order | 10.50 | 2.67 | 3.93 | **0** |
|  | Adapedonta (Order) | Positive | -0.09 | 0.12 | -0.81 | 0.42 |
|  |  | Negative | -0.07 | 0.13 | -0.52 | 0.6 |
|  | Pharidae (Family) | Positive | -0.09 | 0.12 | -0.81 | 0.42 |
|  |  | Negative | -0.07 | 0.13 | -0.52 | 0.6 |
|  | Ensis (Genus) | Positive | -0.09 | 0.12 | -0.81 | 0.42 |
|  |  | Negative | -0.07 | 0.13 | -0.52 | 0.6 |
|  | Cardiida (Order) | Positive | -5.78 | 3.05 | -1.89 | 0.06 |
|  |  | Positive 2nd order | 4.89 | 3.02 | 1.62 | 0.11 |
|  |  | Negative | -6.44 | 2.73 | -2.36 | **0.02** |
|  |  | Negative 2nd order | 13.47 | 2.71 | 4.97 | **0** |
|  | Cardiidae (Family) | Positive | -0.01 | 0.04 | -0.14 | 0.89 |
|  |  | Negative | 0.01 | 0.05 | 0.12 | 0.91 |
|  | Cerastoderma (Genus) | Positive | -0.01 | 0.04 | -0.14 | 0.89 |
|  |  | Negative | 0.01 | 0.05 | 0.12 | 0.91 |
|  | Semelidae (Family) | Positive | -0.01 | 0.01 | -0.75 | 0.45 |
|  |  | Negative | -0.01 | 0.01 | -1.17 | 0.24 |
|  | Scrobicularia (Genus) | Positive | -0.01 | 0.06 | -0.16 | 0.88 |
|  |  | Negative | -0.01 | 0.07 | -0.21 | 0.84 |
|  | Tellinidae (Family) | Positive | -9.21 | 5.12 | -1.80 | 0.07 |
|  |  | Positive 2nd order | 6.74 | 5.10 | 1.32 | 0.19 |
|  |  | Negative | -10.38 | 2.81 | -3.69 | **0** |
|  |  | Negative 2nd order | 16.20 | 2.77 | 5.85 | **0** |
|  | Fabulina (Genus) | Negative | -0.10 | 0.51 | -0.20 | 0.84 |
|  | Macoma (Genus) | Positive | 0.00 | 0.05 | 0.01 | 0.99 |
|  |  | Negative | 0.00 | 0.04 | -0.13 | 0.9 |
|  | Myida (Order) | Positive | 0.03 | 0.08 | 0.44 | 0.66 |
|  |  | Negative | 0.00 | 0.05 | 0.07 | 0.95 |
|  | Myidae (Family) | Positive | 0.03 | 0.08 | 0.44 | 0.66 |
|  |  | Negative | 0.00 | 0.05 | 0.07 | 0.95 |
|  | Mya (Genus) | Positive | 0.03 | 0.08 | 0.44 | 0.66 |
|  |  | Negative | 0.00 | 0.05 | 0.07 | 0.95 |
|  | Mytilida (Order) | Positive | 0.05 | 0.10 | 0.48 | 0.63 |
|  |  | Negative | -0.01 | 0.11 | -0.11 | 0.91 |
|  | Mytilidae (Family) | Positive | 0.05 | 0.10 | 0.48 | 0.63 |
|  |  | Negative | -0.01 | 0.11 | -0.11 | 0.91 |
|  | Mytilus (Genus) | Positive | 0.05 | 0.10 | 0.48 | 0.63 |
|  |  | Negative | -0.01 | 0.11 | -0.11 | 0.91 |
|  | Gastropoda (Class) | Positive | -0.65 | 4.05 | -0.16 | 0.87 |
|  |  | Positive 2nd order | -6.87 | 4.05 | -1.70 | 0.09 |
|  |  | Negative | 3.14 | 2.36 | 1.33 | 0.18 |
|  |  | Negative 2nd order | -2.75 | 2.36 | -1.17 | 0.24 |
|  | Cephalaspidea (Order) | Negative | 0.04 | 0.13 | 0.29 | 0.77 |
|  | Retusidae (Family) | Negative | 0.04 | 0.13 | 0.29 | 0.77 |
|  | Retusa (Genus) | Negative | 0.04 | 0.13 | 0.29 | 0.77 |
|  | Littorinimorpha (Order) | Positive | 0.00 | 0.01 | -0.37 | 0.71 |
|  |  | Negative | 0.00 | 0.01 | -0.22 | 0.82 |
|  | Hydrobiidae (Family) | Positive | 0.00 | 0.06 | -0.03 | 0.98 |
|  |  | Negative | 0.00 | 0.05 | -0.02 | 0.98 |
|  | Peringia (Genus) | Positive | 0.00 | 0.06 | -0.03 | 0.98 |
|  |  | Negative | 0.00 | 0.05 | -0.02 | 0.98 |
| Nemertea | Nemertea (Phylum) | Positive | 0.15 | 0.17 | 0.85 | 0.4 |
|  |  | Negative | 0.01 | 0.11 | 0.09 | 0.93 |
| Tracheophyta | Tracheophyta (Phylum) | Positive | 2.95 | 4.62 | 0.64 | 0.52 |
|  |  | Positive 2nd order | 16.14 | 3.93 | 4.10 | **0** |
|  |  | Negative | -0.61 | 4.22 | -0.15 | 0.88 |
|  |  | Negative 2nd order | 10.46 | 3.49 | 2.99 | **0** |
|  | Equisetopsida (Class) | Positive | 492.49 | 305.46 | 1.61 | 0.11 |
|  |  | Positive 2nd order | -232.34 | 143.72 | -1.62 | 0.11 |
|  |  | Negative | -18.03 | 9.89 | -1.82 | 0.07 |
|  |  | Negative 2nd order | 8.76 | 6.38 | 1.37 | 0.17 |
|  | Ericales (Order) | Positive | 0.00 | 0.61 | 0.00 | 1 |
|  | Primulaceae (Family) | Positive | 0.00 | 0.61 | 0.00 | 1 |
|  | Glaux (Genus) | Positive | 0.00 | 0.61 | 0.00 | 1 |
|  | Lamiales (Order) | Positive | 2.46 | 451.65 | 0.01 | 1 |
|  |  | Positive 2nd order | -1.18 | 216.08 | -0.01 | 1 |
|  |  | Negative | -550.28 | 407.16 | -1.35 | 0.18 |
|  |  | Negative 2nd order | 264.00 | 194.75 | 1.36 | 0.18 |
|  | Plantaginaceae (Family) | Positive | 2.46 | 451.65 | 0.01 | 1 |
|  |  | Positive 2nd order | -1.18 | 216.08 | -0.01 | 1 |
|  |  | Negative | -550.28 | 407.16 | -1.35 | 0.18 |
|  |  | Negative 2nd order | 264.00 | 194.75 | 1.36 | 0.18 |
|  | Plantago (Genus) | Positive | 3.61 | 450.98 | 0.01 | 0.99 |
|  |  | Positive 2nd order | -1.75 | 218.12 | -0.01 | 0.99 |
|  |  | Negative | -541.34 | 399.24 | -1.36 | 0.18 |
|  |  | Negative 2nd order | 262.57 | 193.05 | 1.36 | 0.17 |
|  | Saxifragales (Order) | Negative | 0.00 | 0.55 | 0.00 | 1 |
|  | Crassulaceae (Family) | Negative | 0.00 | 0.55 | 0.00 | 1 |
|  | Sedum (Genus) | Negative | 0.00 | 0.55 | 0.00 | 1 |
|  | Liliopsida (Class) | Positive | -4.99 | 4.63 | -1.08 | 0.28 |
|  |  | Positive 2nd order | 9.42 | 4.12 | 2.28 | **0.02** |
|  |  | Negative | 0.19 | 4.67 | 0.04 | 0.97 |
|  |  | Negative 2nd order | 11.38 | 3.99 | 2.85 | **0** |
|  | Poales (Order) | Positive | -4.99 | 4.63 | -1.08 | 0.28 |
|  |  | Positive 2nd order | 9.42 | 4.12 | 2.28 | **0.02** |
|  |  | Negative | 0.19 | 4.67 | 0.04 | 0.97 |
|  |  | Negative 2nd order | 11.38 | 3.99 | 2.85 | **0** |
|  | Juncaceae (Family) | Positive | 0.00 | 0.12 | 0.00 | 1 |
|  | Juncus (Genus) | Positive | 0.00 | 0.12 | 0.00 | 1 |
|  | Poaceae (Family) | Positive | -4.42 | 4.35 | -1.02 | 0.31 |
|  |  | Positive 2nd order | 8.83 | 3.88 | 2.28 | **0.02** |
|  |  | Negative | 0.53 | 4.39 | 0.12 | 0.9 |
|  |  | Negative 2nd order | 10.64 | 3.76 | 2.83 | **0** |
|  | Festuca (Genus) | Positive | -0.02 | 0.05 | -0.36 | 0.72 |
|  |  | Negative | -0.01 | 0.05 | -0.10 | 0.92 |
|  | Puccinellia (Genus) | Positive | 0.04 | 0.42 | 0.08 | 0.93 |
|  |  | Negative | 0.47 | 0.34 | 1.39 | 0.17 |
|  | Sporobolus (Genus) | Positive | 0.02 | 0.47 | 0.03 | 0.97 |
|  |  | Negative | 0.32 | 0.40 | 0.80 | 0.42 |
|  | Thinopyrum (Genus) | Positive | -0.15 | 0.18 | -0.84 | 0.4 |
|  |  | Negative | -0.15 | 0.12 | -1.18 | 0.24 |
|  | Magnoliopsida (Class) | Positive | 0.14 | 0.02 | 6.13 | **0** |
|  |  | Negative | 0.00 | 0.01 | 0.04 | 0.97 |
|  | Alismatales (Order) | Positive | 37.05 | 13.80 | 2.68 | **0.01** |
|  |  | Positive 2nd order | -36.09 | 13.00 | -2.78 | **0.01** |
|  | Juncaginaceae (Family) | Positive | 640.13 | 845.54 | 0.76 | 0.45 |
|  |  | Positive 2nd order | -375.77 | 474.11 | -0.79 | 0.43 |
|  | Triglochin (Genus) | Positive | 640.13 | 845.54 | 0.76 | 0.45 |
|  |  | Positive 2nd order | -375.77 | 474.11 | -0.79 | 0.43 |
|  | Zosteraceae (Family) | Positive | 0.00 | 0.08 | -0.04 | 0.96 |
|  | Zostera (Genus) | Positive | 0.00 | 0.38 | 0.00 | 1 |
|  | Asterales (Order) | Positive | 617.95 | 544.81 | 1.13 | 0.26 |
|  |  | Positive 2nd order | -355.30 | 310.29 | -1.15 | 0.25 |
|  |  | Negative | 86.91 | 77.37 | 1.12 | 0.26 |
|  |  | Negative 2nd order | -26.93 | 30.56 | -0.88 | 0.38 |
|  | Asteraceae (Family) | Positive | 0.05 | 0.14 | 0.33 | 0.74 |
|  |  | Negative | 0.05 | 0.14 | 0.32 | 0.75 |
|  | Artemisia (Genus) | Positive | 0.05 | 0.14 | 0.33 | 0.74 |
|  |  | Negative | 0.05 | 0.14 | 0.32 | 0.75 |
|  | Compositae (Family) | Negative | 0.31 | 0.35 | 0.89 | 0.37 |
|  | Tripolium (Genus) | Negative | 0.31 | 0.35 | 0.89 | 0.37 |
|  | Caryophyllales (Order) | Positive | 0.28 | 0.04 | 7.74 | **0** |
|  |  | Negative | -0.02 | 0.01 | -1.57 | 0.12 |
|  | Amaranthaceae (Family) | Positive | 0.31 | 0.05 | 6.65 | **0** |
|  |  | Negative | -0.01 | 0.01 | -1.11 | 0.27 |
|  | Atriplex (Genus) | Positive | 0.43 | 0.08 | 5.20 | **0** |
|  |  | Negative | -0.01 | 0.01 | -1.17 | 0.24 |
|  | Salicornia (Genus) | Negative | -0.27 | 0.28 | -0.96 | 0.34 |
|  | Suaeda (Genus) | Positive | 69.42 | 46.57 | 1.49 | 0.14 |
|  |  | Positive 2nd order | 32.29 | 25.24 | 1.28 | 0.2 |
|  |  | Negative | 67.34 | 46.89 | 1.44 | 0.15 |
|  |  | Negative 2nd order | 32.62 | 24.78 | 1.32 | 0.19 |
|  | Caryophyllaceae (Family) | Negative | -0.11 | 0.50 | -0.23 | 0.82 |
|  | Arenaria (Genus) | Positive | -0.01 | 0.14 | -0.07 | 0.95 |
|  | Plumbaginaceae (Family) | Positive | 9.03 | 3.19 | 2.83 | **0** |
|  |  | Positive 2nd order | -1.46 | 3.24 | -0.45 | 0.65 |
|  |  | Negative | -29.97 | 15.83 | -1.89 | 0.06 |
|  |  | Negative 2nd order | -16.62 | 9.27 | -1.79 | 0.07 |
|  | Limonium (Genus) | Positive | 0.21 | 0.22 | 0.95 | 0.34 |
|  |  | Negative | -0.16 | 0.24 | -0.67 | 0.51 |

####

#### **Table S4.** Meta-analysis results used to assign the winner and loser status on phylum level. The meta-analysis was run on the full dataset.

| **Phylum** | **Estimate** | **Std. Error** | **z-value** | **CI (lower)** | **CI (upper)** | **p-value** |
| --- | --- | --- | --- | --- | --- | --- |
| Annelida | 0.018 | 0.005 | 3.615 | 0.008 | 0.027 | <0.001 |
| Arthropoda | 0.013 | 0.005 | 2.597 | 0.003 | 0.022 | 0.009 |
| Cercozoa | 0.021 | 0.030 | 0.677 | -0.039 | 0.080 | 0.498 |
| Chlorophyta | -0.014 | 0.017 | -0.838 | -0.048 | 0.019 | 0.402 |
| Choanozoa | -0.528 | 0.095 | -5.585 | -0.713 | -0.343 | <0.001 |
| Chordata | -0.002 | 0.005 | -0.389 | -0.012 | 0.008 | 0.697 |
| Ciliophora | -0.030 | 0.023 | -1.325 | -0.075 | 0.015 | 0.185 |
| Cnidaria | 0.109 | 0.080 | 1.369 | -0.047 | 0.266 | 0.171 |
| Cryptista | -0.166 | 0.047 | -3.570 | -0.257 | -0.075 | <0.001 |
| Cryptophyta | 0.010 | 0.015 | 0.666 | -0.019 | 0.039 | 0.506 |
| Cyanobacteria | -0.774 | 0.170 | -4.539 | -1.108 | -0.440 | <0.001 |
| Dinoflagellata | -0.011 | 0.014 | -0.837 | -0.038 | 0.015 | 0.402 |
| Echinodermata | 0.058 | 0.014 | 4.214 | 0.031 | 0.085 | <0.001 |
| Euglenophyta | -0.002 | 0.018 | -0.113 | -0.036 | 0.032 | 0.910 |
| Haptophyta | 0.129 | 0.051 | 2.530 | 0.029 | 0.229 | 0.011 |
| Heterokontophyta | -0.016 | 0.013 | -1.258 | -0.042 | 0.009 | 0.208 |
| Mollusca | -0.005 | 0.005 | -1.085 | -0.015 | 0.004 | 0.278 |
| Nemertea | 0.001 | 0.010 | 0.054 | -0.019 | 0.020 | 0.957 |
| Phoronida | -0.043 | 0.205 | -0.211 | -0.445 | 0.358 | 0.833 |
| Tracheophyta | -0.042 | 0.006 | -7.514 | -0.052 | -0.031 | <0.001 |

#### **Table S5.** Meta-analysis results used to assign the winner and loser status on class level. The meta-analysis was run on the full dataset.

| **Class** | **Estimate** | **Std. Error** | **z-value** | **CI (lower)** | **CI (upper)** | **p-value** |
| --- | --- | --- | --- | --- | --- | --- |
| Appendicularia | -0.050 | 0.054 | -0.913 | -0.156 | 0.057 | 0.361 |
| Asteroidea | -0.074 | 0.078 | -0.953 | -0.226 | 0.078 | 0.341 |
| Aves | 0.027 | 0.006 | 4.322 | 0.015 | 0.039 | <0.001 |
| Bacillariophyceae | -0.049 | 0.014 | -3.599 | -0.076 | -0.022 | <0.001 |
| Bivalvia | -0.010 | 0.005 | -1.931 | -0.020 | <0.001 | 0.054 |
| Chlorodendrophyceae | -0.541 | 0.127 | -4.255 | -0.790 | -0.292 | <0.001 |
| Chlorophyceae | -0.032 | 0.024 | -1.337 | -0.080 | 0.015 | 0.181 |
| Chrysophyceae | -0.363 | 0.120 | -3.035 | -0.598 | -0.129 | 0.002 |
| Clitellata | 0.040 | 0.007 | 6.045 | 0.027 | 0.053 | <0.001 |
| Coccolithophyceae | 0.118 | 0.051 | 2.319 | 0.018 | 0.217 | 0.020 |
| Copepoda | -0.058 | 0.030 | -1.965 | -0.117 | <0.001 | 0.049 |
| Coscinodiscophyceae | -0.035 | 0.013 | -2.715 | -0.061 | -0.010 | 0.007 |
| Cryptophyceae | -0.002 | 0.014 | -0.144 | -0.030 | 0.026 | 0.886 |
| Cyanophyceae | -0.786 | 0.170 | -4.613 | -1.120 | -0.452 | <0.001 |
| Dictyochophyceae | -0.075 | 0.058 | -1.299 | -0.189 | 0.038 | 0.194 |
| Dinophyceae | -0.023 | 0.013 | -1.818 | -0.048 | 0.002 | 0.069 |
| Echinoidea | -0.152 | 0.097 | -1.570 | -0.341 | 0.038 | 0.117 |
| Equisetopsida | -0.159 | 0.011 | -14.803 | -0.180 | -0.138 | <0.001 |
| Euglenophyceae | -0.014 | 0.017 | -0.858 | -0.047 | 0.019 | 0.391 |
| Gastropoda | -0.010 | 0.009 | -1.039 | -0.028 | 0.009 | 0.299 |
| Hexacorallia | 0.105 | 0.080 | 1.318 | -0.051 | 0.262 | 0.187 |
| Katablepharidophyceae | -0.177 | 0.046 | -3.828 | -0.268 | -0.086 | <0.001 |
| Liliopsida | -0.039 | 0.006 | -6.529 | -0.050 | -0.027 | <0.001 |
| Litostomatea | -0.098 | 0.041 | -2.376 | -0.178 | -0.017 | 0.018 |
| Magnoliopsida | -0.053 | 0.006 | -8.338 | -0.065 | -0.041 | <0.001 |
| Malacostraca | 0.008 | 0.005 | 1.653 | -0.002 | 0.018 | 0.098 |
| Mamiellophyceae | 0.270 | 0.087 | 3.100 | 0.099 | 0.441 | 0.002 |
| Mediophyceae | -0.021 | 0.012 | -1.717 | -0.045 | 0.003 | 0.086 |
| not assigned | 0.001 | 0.010 | 0.114 | -0.018 | 0.020 | 0.909 |
| Oligotrichea | -0.026 | 0.025 | -1.039 | -0.074 | 0.023 | 0.299 |
| Ophiuroidae | 0.058 | 0.014 | 4.102 | 0.030 | 0.085 | <0.001 |
| Polychaeta | 0.013 | 0.005 | 2.564 | 0.003 | 0.022 | 0.010 |
| Polyplacophora | -0.093 | 0.033 | -2.831 | -0.157 | -0.029 | 0.005 |
| Pyramimonadophyceae | -0.026 | 0.018 | -1.455 | -0.061 | 0.009 | 0.146 |
| Raphidophyceae | 0.006 | 0.027 | 0.206 | -0.048 | 0.059 | 0.837 |
| Teleostei | -0.010 | 0.005 | -1.836 | -0.020 | 0.001 | 0.066 |
| Telonemea | -0.367 | 0.122 | -3.002 | -0.606 | -0.127 | 0.003 |
| Thecofilosea | 0.010 | 0.030 | 0.332 | -0.049 | 0.069 | 0.740 |
| Thecostraca | 0.020 | 0.022 | 0.898 | -0.023 | 0.062 | 0.369 |

#### **Table S6.** Meta-analysis results used to assign the winner and loser status on order level. The meta-analysis was run on the full dataset. MZB stands for macrozoobenthos. The subsets of the meta-analyses were grouped as follows: (i) plants, (ii) phytoplankton, (iii) fish, (iv) birds, (v) macrozoobenthos and zooplankton.

| **Order** | **Group** | **Estimate** | **Std. Error** | **z-value** | **CI (lower)** | **CI (upper)** | **p-value** |
| --- | --- | --- | --- | --- | --- | --- | --- |
| Alismatales | Plants | -0.014 | 0.018 | -0.787 | -0.050 | 0.021 | 0.431 |
| Asterales | Plants | -0.054 | 0.018 | -2.944 | -0.091 | -0.018 | 0.003 |
| Caryophyllales | Plants | -0.033 | 0.016 | -2.044 | -0.064 | -0.001 | 0.041 |
| Ericales | Plants | 0.159 | 0.061 | 2.603 | 0.039 | 0.279 | 0.009 |
| Fabales | Plants | -0.088 | 0.350 | -0.252 | -0.775 | 0.598 | 0.801 |
| Lamiales | Plants | -0.176 | 0.019 | -9.122 | -0.214 | -0.138 | <0.001 |
| Poales | Plants | -0.025 | 0.016 | -1.598 | -0.055 | 0.006 | 0.110 |
| Saxifragales | Plants | -0.133 | 0.022 | -6.045 | -0.176 | -0.090 | <0.001 |
| Amphidiniales | Phytoplankton | -0.044 | 0.070 | -0.630 | -0.180 | 0.093 | 0.529 |
| Anaulales | Phytoplankton | -0.007 | 0.016 | -0.454 | -0.037 | 0.023 | 0.650 |
| Bacillariales | Phytoplankton | -0.080 | 0.020 | -3.945 | -0.119 | -0.040 | <0.001 |
| Biddulphiales | Phytoplankton | -0.029 | 0.026 | -1.109 | -0.081 | 0.022 | 0.268 |
| Chaetocerotales | Phytoplankton | -0.026 | 0.014 | -1.868 | -0.054 | 0.001 | 0.062 |
| Chattonellales | Phytoplankton | 0.011 | 0.027 | 0.394 | -0.042 | 0.063 | 0.694 |
| Chlamydomonadales | Phytoplankton | -0.156 | 0.071 | -2.197 | -0.295 | -0.017 | 0.028 |
| Chromulinales | Phytoplankton | -0.354 | 0.120 | -2.959 | -0.588 | -0.120 | 0.003 |
| Chroococcales | Phytoplankton | -0.777 | 0.170 | -4.563 | -1.111 | -0.444 | <0.001 |
| Clorodendrales | Phytoplankton | -0.536 | 0.127 | -4.218 | -0.785 | -0.287 | <0.001 |
| Coscinodiscales | Phytoplankton | 0.041 | 0.017 | 2.444 | 0.008 | 0.073 | 0.015 |
| Cryomonadida | Phytoplankton | 0.070 | 0.224 | 0.311 | -0.370 | 0.509 | 0.756 |
| Cryptomonadales | Phytoplankton | -0.001 | 0.014 | -0.038 | -0.029 | 0.028 | 0.970 |
| Cymatosirales | Phytoplankton | 0.009 | 0.016 | 0.575 | -0.022 | 0.040 | 0.565 |
| Dictyochales | Phytoplankton | 0.082 | 0.188 | 0.436 | -0.286 | 0.450 | 0.663 |
| Ebriales | Phytoplankton | 0.009 | 0.030 | 0.306 | -0.050 | 0.068 | 0.759 |
| Eupodiscales | Phytoplankton | -0.005 | 0.013 | -0.374 | -0.030 | 0.020 | 0.709 |
| Eutreptiales | Phytoplankton | -0.009 | 0.016 | -0.563 | -0.041 | 0.023 | 0.573 |
| Gonyaulacales | Phytoplankton | -0.022 | 0.022 | -1.011 | -0.065 | 0.021 | 0.312 |
| Gymnodiniales | Phytoplankton | -0.032 | 0.015 | -2.083 | -0.063 | -0.002 | 0.037 |
| Hemiaulales | Phytoplankton | -0.021 | 0.025 | -0.820 | -0.070 | 0.029 | 0.412 |
| Katablepharidales | Phytoplankton | -0.169 | 0.046 | -3.659 | -0.259 | -0.078 | <0.001 |
| Lithodesmiales | Phytoplankton | 0.005 | 0.017 | 0.324 | -0.028 | 0.038 | 0.746 |
| Mamiellales | Phytoplankton | 0.277 | 0.087 | 3.170 | 0.106 | 0.448 | 0.002 |
| Melosirales | Phytoplankton | -0.116 | 0.072 | -1.604 | -0.257 | 0.026 | 0.109 |
| Naviculales | Phytoplankton | -0.003 | 0.018 | -0.141 | -0.038 | 0.033 | 0.888 |
| Noctilucales | Phytoplankton | 0.029 | 0.027 | 1.051 | -0.025 | 0.082 | 0.293 |
| Oligotrichida | Phytoplankton | -0.018 | 0.024 | -0.732 | -0.065 | 0.030 | 0.464 |
| Paraliales | Phytoplankton | -0.057 | 0.014 | -4.097 | -0.084 | -0.030 | <0.001 |
| Pedinellales | Phytoplankton | -0.087 | 0.060 | -1.443 | -0.206 | 0.031 | 0.149 |
| Peridiniales | Phytoplankton | -0.017 | 0.013 | -1.360 | -0.042 | 0.008 | 0.174 |
| Phaeocystales | Phytoplankton | 0.127 | 0.051 | 2.512 | 0.028 | 0.226 | 0.012 |
| Prorocentrales | Phytoplankton | -0.003 | 0.018 | -0.183 | -0.039 | 0.032 | 0.855 |
| Pyramimonadales | Phytoplankton | -0.019 | 0.017 | -1.091 | -0.053 | 0.015 | 0.275 |
| Pyrenomonadales | Phytoplankton | -0.334 | 0.039 | -8.555 | -0.410 | -0.257 | <0.001 |
| Rhaphoneidales | Phytoplankton | -0.066 | 0.022 | -3.087 | -0.109 | -0.024 | 0.002 |
| Rhaponeidales | Phytoplankton | -0.022 | 0.018 | -1.203 | -0.057 | 0.014 | 0.229 |
| Rhizosoleniales | Phytoplankton | -0.028 | 0.014 | -2.031 | -0.055 | -0.001 | 0.042 |
| Sphaeropleales | Phytoplankton | -0.011 | 0.025 | -0.454 | -0.060 | 0.038 | 0.650 |
| Stephanodiscales | Phytoplankton | -0.176 | 0.209 | -0.842 | -0.585 | 0.233 | 0.400 |
| Surirellales | Phytoplankton | -0.077 | 0.051 | -1.518 | -0.176 | 0.022 | 0.129 |
| Telonemida | Phytoplankton | -0.358 | 0.122 | -2.933 | -0.597 | -0.119 | 0.003 |
| Thalassionematales | Phytoplankton | -0.060 | 0.020 | -3.037 | -0.099 | -0.021 | 0.002 |
| Thalassiosirales | Phytoplankton | -0.057 | 0.015 | -3.854 | -0.086 | -0.028 | <0.001 |
| Thoracosphaerales | Phytoplankton | -0.008 | 0.025 | -0.312 | -0.057 | 0.042 | 0.755 |
| Torodiniales | Phytoplankton | -0.004 | 0.042 | -0.108 | -0.086 | 0.077 | 0.914 |
| Triceratiales | Phytoplankton | 0.025 | 0.047 | 0.538 | -0.067 | 0.118 | 0.591 |
| Anguilliformes | Fish | -0.051 | 0.018 | -2.877 | -0.086 | -0.016 | 0.004 |
| Beloniformes | Fish | -0.030 | 0.018 | -1.685 | -0.066 | 0.005 | 0.092 |
| Callionymiformes | Fish | -0.038 | 0.095 | -0.400 | -0.224 | 0.148 | 0.690 |
| Carangiformes | Fish | 0.082 | 0.020 | 4.150 | 0.044 | 0.121 | <0.001 |
| Clupeiformes | Fish | 0.012 | 0.006 | 1.877 | -0.001 | 0.024 | 0.061 |
| Gadiformes | Fish | -0.031 | 0.006 | -5.121 | -0.042 | -0.019 | <0.001 |
| Gobiiformes | Fish | -0.027 | 0.008 | -3.628 | -0.042 | -0.013 | <0.001 |
| Osmeriformes | Fish | -0.006 | 0.006 | -0.979 | -0.018 | 0.006 | 0.328 |
| Perciformes | Fish | -0.015 | 0.005 | -2.824 | -0.025 | -0.005 | 0.005 |
| Petromyzontiformes | Fish | -0.027 | 0.025 | -1.094 | -0.076 | 0.022 | 0.274 |
| Pleuronectiformes | Fish | -0.021 | 0.005 | -4.295 | -0.030 | -0.011 | <0.001 |
| Syngnathiformes | Fish | -0.028 | 0.008 | -3.547 | -0.043 | -0.012 | <0.001 |
| Anseriformes | Birds | 0.049 | 0.007 | 7.453 | 0.036 | 0.062 | <0.001 |
| Charadriiformes | Birds | 0.036 | 0.007 | 5.450 | 0.023 | 0.049 | <0.001 |
| Ciconiiformes | Birds | 0.153 | 0.007 | 21.772 | 0.140 | 0.167 | <0.001 |
| Pelecaniformes | Birds | 0.104 | 0.008 | 12.410 | 0.087 | 0.120 | <0.001 |
| Actiniaria | MZB | 0.109 | 0.080 | 1.365 | -0.048 | 0.266 | 0.172 |
| Adapedonta | MZB | 0.077 | 0.010 | 8.006 | 0.058 | 0.096 | <0.001 |
| Amphipoda | MZB | -0.018 | 0.006 | -3.029 | -0.030 | -0.006 | 0.002 |
| Balanomorpha | MZB | 0.004 | 0.022 | 0.203 | -0.038 | 0.047 | 0.839 |
| Calanoida | Zooplankton | -0.107 | 0.030 | -3.599 | -0.165 | -0.049 | <0.001 |
| Capitellida | MZB | -0.005 | 0.005 | -1.070 | -0.015 | 0.004 | 0.285 |
| Cardiida | MZB | -0.034 | 0.005 | -6.793 | -0.043 | -0.024 | <0.001 |
| Cephalaspidea | MZB | -0.093 | 0.019 | -4.944 | -0.130 | -0.056 | <0.001 |
| Chitonida | MZB | -0.093 | 0.033 | -2.822 | -0.158 | -0.028 | 0.005 |
| Cirratulida | MZB | 0.013 | 0.010 | 1.307 | -0.007 | 0.033 | 0.191 |
| Copelata | Zooplankton | -0.038 | 0.056 | -0.689 | -0.147 | 0.071 | 0.491 |
| Cumacea | MZB | 0.085 | 0.027 | 3.211 | 0.033 | 0.137 | 0.001 |
| Cyclopoida | MZB | 0.364 | 0.083 | 4.397 | 0.202 | 0.526 | <0.001 |
| Cyclotrichiida | Zooplankton | -0.131 | 0.043 | -3.075 | -0.215 | -0.048 | 0.002 |
| Decapoda | MZB | -0.007 | 0.005 | -1.506 | -0.017 | 0.002 | 0.132 |
| Forcipulatida | MZB | -0.078 | 0.079 | -0.991 | -0.232 | 0.076 | 0.322 |
| Galeommatida | MZB | 0.013 | 0.047 | 0.275 | -0.079 | 0.105 | 0.783 |
| Harpacticoida | Zooplankton | 0.170 | 0.093 | 1.823 | -0.013 | 0.353 | 0.068 |
| Isopoda | MZB | -0.101 | 0.065 | -1.558 | -0.229 | 0.026 | 0.119 |
| Littorinimorpha | MZB | -0.010 | 0.010 | -1.056 | -0.030 | 0.009 | 0.291 |
| Myida | MZB | -0.016 | 0.005 | -2.906 | -0.026 | -0.005 | 0.004 |
| Mysida | Zooplankton | -0.066 | 0.022 | -3.020 | -0.108 | -0.023 | 0.003 |
| Mytilida | MZB | -0.012 | 0.010 | -1.211 | -0.031 | 0.007 | 0.226 |
| Ophiurida | MZB | 0.034 | 0.017 | 2.058 | 0.002 | 0.067 | 0.040 |
| Ostreida | MZB | 0.247 | 0.056 | 4.384 | 0.136 | 0.357 | <0.001 |
| Phyllodocida | MZB | -0.003 | 0.005 | -0.625 | -0.013 | 0.007 | 0.532 |
| Sabellida | MZB | -0.038 | 0.097 | -0.395 | -0.228 | 0.152 | 0.693 |
| Spatangoida | MZB | -0.162 | 0.096 | -1.677 | -0.351 | 0.027 | 0.093 |
| Spionida | MZB. Zooplankton | -0.012 | 0.005 | -2.242 | -0.022 | -0.002 | 0.025 |
| Terebellida | MZB. Zooplankton | 0.024 | 0.007 | 3.644 | 0.011 | 0.037 | <0.001 |
| Tubificida | MZB | 0.031 | 0.007 | 4.593 | 0.018 | 0.045 | <0.001 |
| Veneroida | MZB | 0.060 | 0.142 | 0.422 | -0.218 | 0.337 | 0.673 |

#### **Table S7.** Meta-analysis results used to assign the winner and loser status on family level. The meta-analysis was run on the full dataset. MZB stands for macrozoobenthos. The subsets of the meta-analyses were grouped as follows: (i) plants, (ii) phytoplankton, (iii) fish, (iv) birds, (v) macrozoobenthos and zooplankton.

| **Family** | **Group** | **Estimate** | **Std. Error** | **z-value** | **CI (lower)** | **CI (upper)** | **p-value** |
| --- | --- | --- | --- | --- | --- | --- | --- |
| Amaranthaceae | Plants | -0.038 | 0.017 | -2.266 | -0.070 | -0.005 | 0.023 |
| Asteraceae | Plants | -0.019 | 0.020 | -0.969 | -0.058 | 0.019 | 0.332 |
| Caryophyllaceae | Plants | -0.063 | 0.035 | -1.805 | -0.132 | 0.005 | 0.071 |
| Compositae | Plants | -0.166 | 0.027 | -6.061 | -0.220 | -0.112 | <0.001 |
| Crassulaceae | Plants | -0.124 | 0.022 | -5.580 | -0.168 | -0.081 | <0.001 |
| Cyperaceae | Plants | -0.416 | 0.205 | -2.030 | -0.817 | -0.014 | 0.042 |
| Fabaceae | Plants | -0.079 | 0.350 | -0.227 | -0.766 | 0.607 | 0.821 |
| Juncaceae | Plants | -0.085 | 0.018 | -4.662 | -0.121 | -0.050 | <0.001 |
| Juncaginaceae | Plants | -0.044 | 0.020 | -2.254 | -0.083 | -0.006 | 0.024 |
| Plantaginaceae | Plants | -0.167 | 0.020 | -8.498 | -0.206 | -0.129 | <0.001 |
| Plumbaginaceae | Plants | -0.051 | 0.019 | -2.728 | -0.088 | -0.014 | 0.006 |
| Poaceae | Plants | -0.015 | 0.016 | -0.925 | -0.046 | 0.017 | 0.355 |
| Primulaceae | Plants | 0.155 | 0.061 | 2.529 | 0.035 | 0.275 | 0.011 |
| Zosteraceae | Plants | -0.021 | 0.051 | -0.411 | -0.122 | 0.080 | 0.681 |
| Actinomonadaceae | Phytoplankton | -0.088 | 0.060 | -1.452 | -0.206 | 0.031 | 0.146 |
| Amphidiniaceae | Phytoplankton | -0.047 | 0.070 | -0.677 | -0.183 | 0.089 | 0.499 |
| Anaulaceae | Phytoplankton | -0.007 | 0.016 | -0.448 | -0.037 | 0.024 | 0.654 |
| Asterionellopsidaceae | Phytoplankton | -0.067 | 0.022 | -3.088 | -0.109 | -0.024 | 0.002 |
| Bacillariaceae | Phytoplankton | -0.039 | 0.028 | -1.375 | -0.094 | 0.016 | 0.169 |
| Biddulphiaceae | Phytoplankton | -0.035 | 0.026 | -1.324 | -0.087 | 0.017 | 0.185 |
| Ceratiaceae | Phytoplankton | -0.023 | 0.022 | -1.061 | -0.066 | 0.020 | 0.288 |
| Chaetocerotaceae | Phytoplankton | -0.032 | 0.015 | -2.116 | -0.062 | -0.002 | 0.034 |
| Chlamydomonadaceae | Phytoplankton | -0.155 | 0.071 | -2.186 | -0.294 | -0.016 | 0.029 |
| Chlorodendraceae | Phytoplankton | -0.537 | 0.127 | -4.223 | -0.786 | -0.288 | <0.001 |
| Chroomonadacea | Phytoplankton | -0.306 | 0.078 | -3.945 | -0.458 | -0.154 | <0.001 |
| Coscinodiscaceae | Phytoplankton | 0.051 | 0.122 | 0.417 | -0.188 | 0.290 | 0.677 |
| Cymatosiraceae | Phytoplankton | 0.009 | 0.016 | 0.587 | -0.022 | 0.040 | 0.557 |
| Dictyochaceae | Phytoplankton | 0.079 | 0.188 | 0.423 | -0.289 | 0.448 | 0.672 |
| Dinobryaceae | Phytoplankton | -0.355 | 0.120 | -2.968 | -0.589 | -0.121 | 0.003 |
| Ebriaceae | Phytoplankton | 0.008 | 0.030 | 0.280 | -0.050 | 0.067 | 0.780 |
| Entomoneidaceae | Phytoplankton | -0.078 | 0.051 | -1.544 | -0.177 | 0.021 | 0.123 |
| Eupodiscaceae | Phytoplankton | -0.011 | 0.016 | -0.708 | -0.043 | 0.020 | 0.479 |
| Eutreptiaceae | Phytoplankton | -0.008 | 0.016 | -0.496 | -0.040 | 0.024 | 0.620 |
| Fibrocapsaceae | Phytoplankton | 0.011 | 0.027 | 0.413 | -0.041 | 0.064 | 0.679 |
| Geminigeraceae | Phytoplankton | -0.366 | 0.056 | -6.492 | -0.477 | -0.256 | <0.001 |
| Gymnodiniaceae | Phytoplankton | -0.040 | 0.018 | -2.286 | -0.074 | -0.006 | 0.022 |
| Heliopeltaceae | Phytoplankton | 0.039 | 0.017 | 2.252 | 0.005 | 0.072 | 0.024 |
| Hemiaulaceae | Phytoplankton | -0.023 | 0.025 | -0.904 | -0.072 | 0.027 | 0.366 |
| Hemidiscaceae | Phytoplankton | 0.059 | 0.049 | 1.200 | -0.037 | 0.156 | 0.230 |
| Hemiselmidaceae | Phytoplankton | -0.225 | 0.084 | -2.673 | -0.390 | -0.060 | 0.008 |
| Heterocapsaceae | Phytoplankton | -0.022 | 0.016 | -1.356 | -0.053 | 0.010 | 0.175 |
| Hyalodiscaceae | Phytoplankton | -0.626 | 0.230 | -2.723 | -1.076 | -0.175 | 0.006 |
| Katablepharidaceae | Phytoplankton | -0.168 | 0.046 | -3.654 | -0.259 | -0.078 | <0.001 |
| Kryptoperidiniaceae | Phytoplankton | -0.286 | 0.102 | -2.793 | -0.486 | -0.085 | 0.005 |
| Lauderiaceae | Phytoplankton | -0.016 | 0.037 | -0.436 | -0.088 | 0.056 | 0.663 |
| Leptocylindraceae | Phytoplankton | -0.013 | 0.019 | -0.682 | -0.051 | 0.025 | 0.495 |
| Lithodesmiaceae | Phytoplankton | 0.005 | 0.017 | 0.285 | -0.028 | 0.038 | 0.776 |
| Mamiellaceae | Phytoplankton | 0.278 | 0.087 | 3.190 | 0.107 | 0.449 | 0.001 |
| Melosiraceae | Phytoplankton | -0.060 | 0.076 | -0.785 | -0.208 | 0.089 | 0.432 |
| Naviculaceae | Phytoplankton | -0.003 | 0.018 | -0.168 | -0.039 | 0.033 | 0.866 |
| Noctilucaceae | Phytoplankton | 0.026 | 0.027 | 0.964 | -0.027 | 0.080 | 0.335 |
| Odontellaceae | Phytoplankton | 0.031 | 0.019 | 1.652 | -0.006 | 0.069 | 0.099 |
| Paraliaceae | Phytoplankton | -0.057 | 0.014 | -4.108 | -0.085 | -0.030 | <0.001 |
| Parodontellaceae | Phytoplankton | -0.013 | 0.014 | -0.949 | -0.041 | 0.014 | 0.343 |
| Phaeocystaceae | Phytoplankton | 0.127 | 0.051 | 2.509 | 0.028 | 0.226 | 0.012 |
| Pleurosigmataceae | Phytoplankton | 0.004 | 0.097 | 0.042 | -0.186 | 0.194 | 0.967 |
| Prorocentraceae | Phytoplankton | -0.004 | 0.018 | -0.220 | -0.040 | 0.032 | 0.826 |
| Protaspidae | Phytoplankton | 0.069 | 0.224 | 0.309 | -0.370 | 0.509 | 0.758 |
| Protoperidiniaceae | Phytoplankton | -0.018 | 0.013 | -1.322 | -0.044 | 0.009 | 0.186 |
| Pterospermataceae | Phytoplankton | -0.075 | 0.197 | -0.381 | -0.462 | 0.312 | 0.704 |
| Pyramimonadaceae | Phytoplankton | -0.018 | 0.017 | -1.018 | -0.052 | 0.016 | 0.309 |
| Pyrenomonadaceae | Phytoplankton | -0.471 | 0.116 | -4.078 | -0.698 | -0.245 | <0.001 |
| Rhaphoneidaceae | Phytoplankton | -0.021 | 0.018 | -1.151 | -0.056 | 0.015 | 0.250 |
| Rhizosoleniaceae | Phytoplankton | -0.030 | 0.014 | -2.129 | -0.057 | -0.002 | 0.033 |
| Scenedesmaceae | Phytoplankton | -0.011 | 0.025 | -0.450 | -0.060 | 0.038 | 0.652 |
| Skeletonemaceae | Phytoplankton | -0.010 | 0.033 | -0.304 | -0.075 | 0.054 | 0.761 |
| Stephanodiscaceae | Phytoplankton | -0.175 | 0.209 | -0.841 | -0.585 | 0.234 | 0.401 |
| Telonemidae | Phytoplankton | -0.359 | 0.122 | -2.943 | -0.599 | -0.120 | 0.003 |
| Thalassionemataceae | Phytoplankton | -0.060 | 0.020 | -3.044 | -0.099 | -0.022 | 0.002 |
| Thalassiosiraceae | Phytoplankton | -0.069 | 0.016 | -4.418 | -0.100 | -0.038 | <0.001 |
| Thoracosphaeraceae | Phytoplankton | -0.008 | 0.025 | -0.308 | -0.057 | 0.042 | 0.758 |
| Tontoniidae | Phytoplankton | -0.019 | 0.024 | -0.768 | -0.066 | 0.029 | 0.442 |
| Torodiniaceae | Phytoplankton | -0.003 | 0.042 | -0.067 | -0.084 | 0.079 | 0.947 |
| Triceratiaceae | Phytoplankton | 0.025 | 0.047 | 0.527 | -0.068 | 0.117 | 0.598 |
| Warnowiaceae | Phytoplankton | 0.042 | 0.073 | 0.575 | -0.102 | 0.186 | 0.565 |
| Agonidae | Fish | -0.067 | 0.012 | -5.774 | -0.090 | -0.044 | <0.001 |
| Alosidae | Fish | -0.052 | 0.030 | -1.729 | -0.112 | 0.007 | 0.083 |
| Ammodytidae | Fish | 0.076 | 0.011 | 6.902 | 0.054 | 0.098 | <0.001 |
| Anguillidae | Fish | -0.048 | 0.018 | -2.725 | -0.083 | -0.014 | 0.006 |
| Belonidae | Fish | -0.027 | 0.018 | -1.485 | -0.062 | 0.009 | 0.138 |
| Callionymidae | Fish | -0.040 | 0.095 | -0.422 | -0.226 | 0.146 | 0.673 |
| Carangidae | Fish | 0.083 | 0.020 | 4.165 | 0.044 | 0.122 | <0.001 |
| Clupeidae | Fish | 0.014 | 0.006 | 2.134 | 0.001 | 0.026 | 0.033 |
| Cottidae | Fish | -0.027 | 0.007 | -4.088 | -0.040 | -0.014 | <0.001 |
| Engraulidae | Fish | -0.081 | 0.096 | -0.845 | -0.269 | 0.107 | 0.398 |
| Gadidae | Fish | -0.046 | 0.007 | -6.780 | -0.059 | -0.032 | <0.001 |
| Gasterosteidae | Fish | -0.018 | 0.057 | -0.322 | -0.131 | 0.094 | 0.747 |
| Gobiidae | Fish | -0.029 | 0.008 | -3.786 | -0.044 | -0.014 | <0.001 |
| Liparidae | Fish | -0.021 | 0.019 | -1.146 | -0.058 | 0.015 | 0.251 |
| Lotidae | Fish | -0.001 | 0.008 | -0.094 | -0.017 | 0.016 | 0.925 |
| Osmeridae | Fish | -0.006 | 0.006 | -0.959 | -0.018 | 0.006 | 0.337 |
| Petromyzontidae | Fish | -0.023 | 0.025 | -0.931 | -0.072 | 0.026 | 0.352 |
| Pholidae | Fish | 0.039 | 0.013 | 3.058 | 0.014 | 0.064 | 0.002 |
| Pleuronectidae | Fish | -0.023 | 0.005 | -4.477 | -0.033 | -0.013 | <0.001 |
| Scophthalmidae | Fish | 0.029 | 0.018 | 1.579 | -0.007 | 0.065 | 0.114 |
| Soleidae | Fish | -0.021 | 0.006 | -3.288 | -0.033 | -0.008 | 0.001 |
| Syngnathidae | Fish | -0.028 | 0.008 | -3.592 | -0.043 | -0.013 | <0.001 |
| Trachinidae | Fish | -0.024 | 0.024 | -0.988 | -0.072 | 0.024 | 0.323 |
| Triglidae | Fish | 0.058 | 0.024 | 2.449 | 0.012 | 0.104 | 0.014 |
| Zoarcidae | Fish | -0.016 | 0.006 | -2.643 | -0.028 | -0.004 | 0.008 |
| Anatidae | Birds | 0.050 | 0.007 | 7.489 | 0.037 | 0.063 | <0.001 |
| Charadriidae | Birds | 0.038 | 0.007 | 5.696 | 0.025 | 0.051 | <0.001 |
| Laridae | Birds | 0.034 | 0.007 | 5.073 | 0.021 | 0.047 | <0.001 |
| Phalacrocoracidae | Birds | 0.105 | 0.008 | 12.494 | 0.088 | 0.121 | <0.001 |
| Recurvirostridae | Birds | 0.025 | 0.007 | 3.707 | 0.012 | 0.039 | <0.001 |
| Scolopacidae | Birds | 0.038 | 0.007 | 5.726 | 0.025 | 0.051 | <0.001 |
| Threskiornithidae | Birds | 0.154 | 0.007 | 21.709 | 0.140 | 0.168 | <0.001 |
| Ampharetidae | MZB | -0.113 | 0.039 | -2.889 | -0.189 | -0.036 | 0.004 |
| Arenicolidae | MZB | -0.009 | 0.005 | -1.813 | -0.019 | 0.001 | 0.070 |
| Asteriidae | MZB | -0.062 | 0.079 | -0.788 | -0.217 | 0.093 | 0.431 |
| Balanidae | MZB | 0.005 | 0.022 | 0.245 | -0.037 | 0.048 | 0.807 |
| Bathyporeiidae | MZB | -0.041 | 0.007 | -5.696 | -0.055 | -0.027 | <0.001 |
| Bodotriidae | MZB | 0.076 | 0.027 | 2.843 | 0.024 | 0.128 | 0.004 |
| Calyptraeidae | MZB | 0.024 | 0.094 | 0.258 | -0.160 | 0.208 | 0.797 |
| Capitellidae | MZB | 0.001 | 0.005 | 0.198 | -0.009 | 0.011 | 0.843 |
| Carcinidae | MZB | -0.012 | 0.005 | -2.266 | -0.022 | -0.002 | 0.023 |
| Cardiidae | MZB | -0.014 | 0.006 | -2.225 | -0.026 | -0.002 | 0.026 |
| Centropagidae | Zooplankton | -0.311 | 0.085 | -3.651 | -0.478 | -0.144 | <0.001 |
| Cirratulidae | MZB | 0.061 | 0.011 | 5.584 | 0.040 | 0.083 | <0.001 |
| Clausocalanidae | Zooplankton | -0.120 | 0.140 | -0.860 | -0.394 | 0.154 | 0.390 |
| Corophiidae | MZB | -0.011 | 0.009 | -1.208 | -0.028 | 0.007 | 0.227 |
| Crangonidae | MZB | -0.007 | 0.005 | -1.383 | -0.017 | 0.003 | 0.167 |
| Donacidae | MZB | 0.060 | 0.142 | 0.422 | -0.218 | 0.337 | 0.673 |
| Fabriciidae | MZB | -0.038 | 0.097 | -0.388 | -0.227 | 0.152 | 0.698 |
| Gammaridae | MZB | 0.019 | 0.011 | 1.641 | -0.004 | 0.041 | 0.101 |
| Hydrobiidae | MZB | -0.011 | 0.010 | -1.090 | -0.030 | 0.009 | 0.276 |
| Janiridae | MZB | -0.091 | 0.065 | -1.402 | -0.219 | 0.036 | 0.161 |
| Lasaeidae | MZB | 0.017 | 0.047 | 0.351 | -0.076 | 0.109 | 0.725 |
| Loveniidae | MZB | -0.162 | 0.097 | -1.676 | -0.351 | 0.027 | 0.094 |
| Magelonidae | MZB. Zooplankton | -0.045 | 0.009 | -5.182 | -0.062 | -0.028 | <0.001 |
| Mesodiniidae | Zooplankton | -0.131 | 0.043 | -3.076 | -0.214 | -0.048 | 0.002 |
| Myidae | MZB | -0.013 | 0.005 | -2.397 | -0.023 | -0.002 | 0.017 |
| Mysidae | Zooplankton | -0.071 | 0.022 | -3.245 | -0.113 | -0.028 | 0.001 |
| Mytilidae | MZB | -0.010 | 0.010 | -1.018 | -0.029 | 0.009 | 0.309 |
| Naididae | MZB | 0.032 | 0.007 | 4.659 | 0.019 | 0.045 | <0.001 |
| Nephtyidae | MZB | -0.052 | 0.006 | -9.371 | -0.063 | -0.041 | <0.001 |
| Nereididae | MZB | 0.010 | 0.005 | 1.871 | <0.001 | 0.020 | 0.061 |
| Oedicerotidae | MZB | 0.007 | 0.047 | 0.153 | -0.086 | 0.100 | 0.878 |
| Oikopleuridae | Zooplankton | -0.025 | 0.056 | -0.451 | -0.135 | 0.084 | 0.652 |
| Oithonidae | Zooplankton | 0.356 | 0.084 | 4.258 | 0.192 | 0.520 | <0.001 |
| Ophiuridae | MZB | 0.036 | 0.017 | 2.109 | 0.003 | 0.069 | 0.035 |
| Orbiniidae | MZB | 0.003 | 0.006 | 0.605 | -0.008 | 0.015 | 0.545 |
| Ostreidae | MZB | 0.257 | 0.056 | 4.558 | 0.146 | 0.367 | <0.001 |
| Paguridae | MZB | 0.013 | 0.012 | 1.146 | -0.010 | 0.036 | 0.252 |
| Paracalanidae | Zooplankton | -0.155 | 0.067 | -2.322 | -0.285 | -0.024 | 0.020 |
| Paraonidae | MZB | 0.005 | 0.010 | 0.513 | -0.014 | 0.025 | 0.608 |
| Pectinariidae | MZB | 0.126 | 0.044 | 2.890 | 0.041 | 0.212 | 0.004 |
| Pharidae | MZB | 0.069 | 0.010 | 7.140 | 0.050 | 0.088 | <0.001 |
| Phoronidae | MZB | -0.045 | 0.205 | -0.222 | -0.447 | 0.356 | 0.824 |
| Phyllodocidae | MZB | 0.013 | 0.005 | 2.524 | 0.003 | 0.023 | 0.012 |
| Polybiidae | MZB | 0.003 | 0.010 | 0.297 | -0.017 | 0.023 | 0.767 |
| Polynoidae | MZB | -0.031 | 0.007 | -4.565 | -0.045 | -0.018 | <0.001 |
| Pseudodiaptomidae | Zooplankton | 0.039 | 0.063 | 0.608 | -0.086 | 0.163 | 0.543 |
| Retusidae | MZB | -0.101 | 0.019 | -5.358 | -0.138 | -0.064 | <0.001 |
| Semelidae | MZB | 0.003 | 0.006 | 0.503 | -0.008 | 0.014 | 0.615 |
| Spionidae | MZB. Zooplankton | -0.015 | 0.005 | -2.803 | -0.026 | -0.005 | 0.005 |
| Syllidae | MZB | 0.135 | 0.070 | 1.935 | -0.002 | 0.271 | 0.053 |
| Tachidiidae | Zooplankton | 0.169 | 0.093 | 1.816 | -0.013 | 0.352 | 0.069 |
| Tellinidae | MZB | -0.050 | 0.005 | -9.825 | -0.060 | -0.040 | <0.001 |
| Temoridae | Zooplankton | -0.113 | 0.041 | -2.739 | -0.194 | -0.032 | 0.006 |
| Terebellidae | MZB. Zooplankton | 0.010 | 0.007 | 1.410 | -0.004 | 0.024 | 0.159 |
| Tonicellidae | MZB | -0.083 | 0.033 | -2.509 | -0.148 | -0.018 | 0.012 |
| Urothoidae | MZB | -0.009 | 0.012 | -0.727 | -0.032 | 0.015 | 0.467 |
| Varunidae | MZB | 0.286 | 0.046 | 6.170 | 0.195 | 0.377 | <0.001 |

#### **Table S8.** Meta-analysis results used to assign the winner and loser status on genus level. The meta-analysis was run on the full dataset. MZB stands for macrozoobenthos. The subsets of the meta-analyses were grouped as follows: (i) plants. (ii) phytoplankton. (iii) fish. (iv) birds. (v) macrozoobenthos and zooplankton.

| **Genus** | **Group** | **Estimate** | **Std. Error** | **z-value** | **CI (lower)** | **CI (upper)** | **p-value** |
| --- | --- | --- | --- | --- | --- | --- | --- |
| Agrostis | Plants | -0.364 | 0.087 | -4.183 | -0.534 | -0.193 | <0.001 |
| Arenaria | Plants | -0.564 | 0.243 | -2.321 | -1.039 | -0.088 | 0.020 |
| Armeria | Plants | 0.105 | 0.093 | 1.136 | -0.076 | 0.287 | 0.256 |
| Artemisia | Plants | 0.004 | 0.020 | 0.195 | -0.035 | 0.043 | 0.846 |
| Atriplex | Plants | -0.011 | 0.017 | -0.678 | -0.045 | 0.022 | 0.498 |
| Carex | Plants | -0.487 | 0.206 | -2.369 | -0.890 | -0.084 | 0.018 |
| Elymus | Plants | 0.124 | 0.018 | 6.957 | 0.089 | 0.159 | <0.001 |
| Festuca | Plants | -0.026 | 0.017 | -1.581 | -0.059 | 0.006 | 0.114 |
| Glaux | Plants | 0.163 | 0.062 | 2.627 | 0.041 | 0.284 | 0.009 |
| Honckenya | Plants | 0.003 | 0.072 | 0.042 | -0.137 | 0.143 | 0.967 |
| Juncus | Plants | -0.073 | 0.019 | -3.924 | -0.109 | -0.037 | <0.001 |
| Limonium | Plants | -0.054 | 0.019 | -2.829 | -0.091 | -0.017 | 0.005 |
| Linaria | Plants | 0.511 | 0.151 | 3.383 | 0.215 | 0.807 | 0.001 |
| Zostera | Plants | 0.038 | 0.073 | 0.521 | -0.105 | 0.182 | 0.602 |
| Plantago | Plants | -0.051 | 0.021 | -2.463 | -0.092 | -0.011 | 0.014 |
| Puccinellia | Plants | -0.095 | 0.028 | -3.408 | -0.150 | -0.041 | 0.001 |
| Sagina | Plants | 0.203 | 0.333 | 0.609 | -0.450 | 0.856 | 0.543 |
| Salicornia | Plants | -0.225 | 0.031 | -7.311 | -0.286 | -0.165 | <0.001 |
| Sedum | Plants | -0.127 | 0.022 | -5.637 | -0.171 | -0.083 | <0.001 |
| Spergularia | Plants | -0.064 | 0.039 | -1.621 | -0.141 | 0.013 | 0.105 |
| Sporobolus | Plants | -0.100 | 0.022 | -4.642 | -0.142 | -0.058 | <0.001 |
| Suaeda | Plants | -0.093 | 0.028 | -3.266 | -0.149 | -0.037 | 0.001 |
| Thinopyrum | Plants | -0.007 | 0.019 | -0.391 | -0.044 | 0.029 | 0.696 |
| Trifolium | Plants | -0.044 | 0.350 | -0.125 | -0.730 | 0.643 | 0.901 |
| Triglochin | Plants | -0.040 | 0.020 | -2.029 | -0.080 | -0.001 | 0.042 |
| Tripolium | Plants | -0.165 | 0.028 | -5.987 | -0.219 | -0.111 | <0.001 |
| Zostera | Plants | -0.080 | 0.073 | -1.094 | -0.222 | 0.063 | 0.274 |
| Actinocyclus | Phytoplankton | 0.062 | 0.049 | 1.259 | -0.035 | 0.159 | 0.208 |
| Actinoptychus | Phytoplankton | 0.038 | 0.017 | 2.270 | 0.005 | 0.071 | 0.023 |
| Amphidinium | Phytoplankton | -0.047 | 0.070 | -0.677 | -0.183 | 0.089 | 0.499 |
| Archaeperidinium | Phytoplankton | -0.667 | 0.555 | -1.202 | -1.754 | 0.420 | 0.229 |
| Asterionellopsis | Phytoplankton | -0.058 | 0.030 | -1.942 | -0.117 | 0.001 | 0.052 |
| Asteroplanus | Phytoplankton | -0.071 | 0.027 | -2.671 | -0.123 | -0.019 | 0.008 |
| Bacteriastrum | Phytoplankton | -0.044 | 0.042 | -1.048 | -0.127 | 0.039 | 0.295 |
| Brockmanniella | Phytoplankton | -0.046 | 0.035 | -1.333 | -0.114 | 0.022 | 0.183 |
| Cerataulina | Phytoplankton | -0.009 | 0.047 | -0.189 | -0.101 | 0.083 | 0.850 |
| Cerataulus | Phytoplankton | 0.082 | 0.041 | 2.000 | 0.002 | 0.162 | 0.045 |
| Chaetoceros | Phytoplankton | -0.028 | 0.015 | -1.823 | -0.058 | 0.002 | 0.068 |
| Chlamydomonas | Phytoplankton | -0.157 | 0.071 | -2.210 | -0.296 | -0.018 | 0.027 |
| Chroomonas | Phytoplankton | -0.227 | 0.084 | -2.702 | -0.392 | -0.062 | 0.007 |
| Coscinodiscus | Phytoplankton | 0.054 | 0.122 | 0.442 | -0.185 | 0.293 | 0.659 |
| Cryothecomonas | Phytoplankton | 0.070 | 0.224 | 0.310 | -0.370 | 0.509 | 0.756 |
| Cyclotella | Phytoplankton | -0.178 | 0.209 | -0.853 | -0.587 | 0.231 | 0.394 |
| Cylindrotheca | Phytoplankton | -0.096 | 0.040 | -2.385 | -0.174 | -0.017 | 0.017 |
| Cymbomonas | Phytoplankton | -0.332 | 0.270 | -1.230 | -0.861 | 0.197 | 0.219 |
| Dactyliosolen | Phytoplankton | 0.007 | 0.040 | 0.183 | -0.072 | 0.087 | 0.855 |
| Delphineis | Phytoplankton | -0.019 | 0.018 | -1.035 | -0.054 | 0.017 | 0.301 |
| Detonula | Phytoplankton | -0.182 | 0.044 | -4.103 | -0.268 | -0.095 | <0.001 |
| Dinobryon | Phytoplankton | -0.356 | 0.120 | -2.979 | -0.590 | -0.122 | 0.003 |
| Diplopsalis | Phytoplankton | -0.032 | 0.017 | -1.931 | -0.065 | <0.001 | 0.053 |
| Ditylum | Phytoplankton | 0.020 | 0.023 | 0.857 | -0.025 | 0.064 | 0.392 |
| Ebria | Phytoplankton | 0.009 | 0.030 | 0.304 | -0.050 | 0.068 | 0.761 |
| Entomoneis | Phytoplankton | -0.079 | 0.051 | -1.555 | -0.178 | 0.020 | 0.120 |
| Eucampia | Phytoplankton | -0.026 | 0.028 | -0.936 | -0.082 | 0.029 | 0.349 |
| Eunotogramma | Phytoplankton | -0.005 | 0.015 | -0.332 | -0.035 | 0.025 | 0.740 |
| Eutreptiella | Phytoplankton | -0.005 | 0.016 | -0.283 | -0.036 | 0.027 | 0.777 |
| Fibrocapsa | Phytoplankton | 0.010 | 0.027 | 0.378 | -0.042 | 0.062 | 0.706 |
| Guinardia | Phytoplankton | -0.048 | 0.018 | -2.625 | -0.083 | -0.012 | 0.009 |
| Gymnodinium | Phytoplankton | -0.097 | 0.089 | -1.086 | -0.272 | 0.078 | 0.277 |
| Gyrodinium | Phytoplankton | -0.036 | 0.017 | -2.045 | -0.070 | -0.001 | 0.041 |
| Gyrosigma | Phytoplankton | -0.011 | 0.020 | -0.579 | -0.050 | 0.027 | 0.562 |
| Hemiselmis | Phytoplankton | -0.309 | 0.078 | -3.985 | -0.461 | -0.157 | <0.001 |
| Heterocapsa | Phytoplankton | -0.019 | 0.016 | -1.191 | -0.049 | 0.012 | 0.234 |
| Katablepharis | Phytoplankton | -0.252 | 0.069 | -3.665 | -0.387 | -0.117 | <0.001 |
| Kryptoperidinium | Phytoplankton | -0.288 | 0.102 | -2.809 | -0.488 | -0.087 | 0.005 |
| Laboea | Phytoplankton | -0.020 | 0.024 | -0.822 | -0.067 | 0.027 | 0.411 |
| Lauderia | Phytoplankton | -0.016 | 0.037 | -0.427 | -0.088 | 0.056 | 0.669 |
| Lebouridinium | Phytoplankton | -0.020 | 0.021 | -0.928 | -0.062 | 0.022 | 0.353 |
| Lennoxia | Phytoplankton | 0.735 | 0.512 | 1.434 | -0.270 | 1.739 | 0.152 |
| Lepidodinium | Phytoplankton | -0.217 | 0.173 | -1.255 | -0.555 | 0.122 | 0.209 |
| Leptocylindrus | Phytoplankton | -0.043 | 0.026 | -1.653 | -0.094 | 0.008 | 0.098 |
| Leucocryptos | Phytoplankton | -0.109 | 0.060 | -1.817 | -0.227 | 0.009 | 0.069 |
| Lithodesmium | Phytoplankton | -0.005 | 0.019 | -0.274 | -0.043 | 0.033 | 0.784 |
| Mediopyxis | Phytoplankton | -0.341 | 0.067 | -5.071 | -0.472 | -0.209 | <0.001 |
| Melosira | Phytoplankton | -0.056 | 0.076 | -0.736 | -0.204 | 0.093 | 0.462 |
| Micromonas | Phytoplankton | 0.279 | 0.087 | 3.196 | 0.108 | 0.450 | 0.001 |
| Minutocellus | Phytoplankton | 0.038 | 0.040 | 0.945 | -0.041 | 0.116 | 0.344 |
| Navicula | Phytoplankton | 0.030 | 0.034 | 0.880 | -0.036 | 0.095 | 0.379 |
| Nematopsides | Phytoplankton | 0.043 | 0.073 | 0.588 | -0.101 | 0.187 | 0.557 |
| Neobrightwellia | Phytoplankton | -0.034 | 0.026 | -1.305 | -0.086 | 0.017 | 0.192 |
| Nitzschia | Phytoplankton | 0.030 | 0.182 | 0.164 | -0.327 | 0.387 | 0.870 |
| Noctiluca | Phytoplankton | 0.024 | 0.027 | 0.874 | -0.030 | 0.077 | 0.382 |
| Octactis | Phytoplankton | 0.083 | 0.188 | 0.439 | -0.286 | 0.451 | 0.660 |
| Odontella | Phytoplankton | 0.032 | 0.019 | 1.702 | -0.005 | 0.069 | 0.089 |
| Paralia | Phytoplankton | -0.057 | 0.014 | -4.158 | -0.083 | -0.030 | <0.001 |
| Peridiniella | Phytoplankton | 0.024 | 0.044 | 0.556 | -0.061 | 0.110 | 0.578 |
| Phaeocystis | Phytoplankton | 0.129 | 0.051 | 2.553 | 0.030 | 0.228 | 0.011 |
| Plagiogrammopsis | Phytoplankton | 0.015 | 0.016 | 0.931 | -0.017 | 0.048 | 0.352 |
| Plagioselmis | Phytoplankton | -0.466 | 0.127 | -3.667 | -0.716 | -0.217 | <0.001 |
| Pleurosigma | Phytoplankton | -0.005 | 0.097 | -0.047 | -0.195 | 0.186 | 0.963 |
| Podosira | Phytoplankton | -0.623 | 0.230 | -2.710 | -1.073 | -0.172 | 0.007 |
| Preperidinium | Phytoplankton | -0.001 | 0.027 | -0.046 | -0.054 | 0.051 | 0.963 |
| Prorocentrum | Phytoplankton | -0.003 | 0.018 | -0.151 | -0.038 | 0.033 | 0.880 |
| Protoperidinium | Phytoplankton | -0.007 | 0.015 | -0.453 | -0.037 | 0.023 | 0.651 |
| Pseudo-nitzschia | Phytoplankton | 0.007 | 0.037 | 0.180 | -0.066 | 0.080 | 0.857 |
| Pseudopedinella | Phytoplankton | -0.088 | 0.060 | -1.465 | -0.207 | 0.030 | 0.143 |
| Pterosperma | Phytoplankton | -0.080 | 0.197 | -0.405 | -0.466 | 0.307 | 0.686 |
| Pyramimonas | Phytoplankton | -0.015 | 0.017 | -0.864 | -0.048 | 0.019 | 0.388 |
| Ralfsiella | Phytoplankton | 0.022 | 0.047 | 0.469 | -0.070 | 0.115 | 0.639 |
| Rhaphoneis | Phytoplankton | 0.013 | 0.091 | 0.146 | -0.166 | 0.192 | 0.884 |
| Rhizosolenia | Phytoplankton | -0.003 | 0.020 | -0.169 | -0.043 | 0.036 | 0.866 |
| Rhodomonas | Phytoplankton | -0.473 | 0.116 | -4.095 | -0.699 | -0.247 | <0.001 |
| Scrippsiella | Phytoplankton | -0.008 | 0.025 | -0.305 | -0.057 | 0.042 | 0.760 |
| Skeletonema | Phytoplankton | -0.009 | 0.033 | -0.276 | -0.073 | 0.055 | 0.783 |
| Sundstroemia | Phytoplankton | -0.033 | 0.017 | -1.936 | -0.066 | <0.001 | 0.053 |
| Teleaulax | Phytoplankton | -0.344 | 0.062 | -5.518 | -0.467 | -0.222 | <0.001 |
| Telonema | Phytoplankton | -0.361 | 0.122 | -2.954 | -0.600 | -0.121 | 0.003 |
| Tetraselmis | Phytoplankton | -0.538 | 0.127 | -4.234 | -0.787 | -0.289 | <0.001 |
| Thalassionema | Phytoplankton | -0.060 | 0.020 | -3.078 | -0.099 | -0.022 | 0.002 |
| Thalassiosira | Phytoplankton | -0.062 | 0.016 | -3.946 | -0.092 | -0.031 | <0.001 |
| Torodinium | Phytoplankton | -0.002 | 0.042 | -0.056 | -0.084 | 0.079 | 0.955 |
| Trieres | Phytoplankton | -0.013 | 0.014 | -0.926 | -0.040 | 0.014 | 0.354 |
| Tripos | Phytoplankton | -0.020 | 0.022 | -0.945 | -0.063 | 0.022 | 0.345 |
| Zygoceros | Phytoplankton | -0.018 | 0.016 | -1.137 | -0.050 | 0.013 | 0.255 |
| Agonus | Fish | -0.071 | 0.012 | -5.977 | -0.094 | -0.048 | <0.001 |
| Alosa | Fish | -0.049 | 0.030 | -1.606 | -0.108 | 0.011 | 0.108 |
| Ammodytes | Fish | 0.083 | 0.012 | 6.699 | 0.059 | 0.108 | <0.001 |
| Anguilla | Fish | -0.049 | 0.018 | -2.742 | -0.084 | -0.014 | 0.006 |
| Belone | Fish | -0.017 | 0.018 | -0.953 | -0.053 | 0.018 | 0.341 |
| Callionymus | Fish | -0.048 | 0.095 | -0.507 | -0.234 | 0.138 | 0.612 |
| Chelidonichthys | Fish | 0.060 | 0.024 | 2.530 | 0.013 | 0.106 | 0.011 |
| Ciliata | Fish | -0.001 | 0.009 | -0.071 | -0.018 | 0.016 | 0.944 |
| Clupea | Fish | 0.021 | 0.007 | 3.038 | 0.008 | 0.035 | 0.002 |
| Echiichthys | Fish | -0.022 | 0.024 | -0.909 | -0.070 | 0.026 | 0.363 |
| Engraulis | Fish | -0.071 | 0.096 | -0.735 | -0.259 | 0.118 | 0.462 |
| Gadus | Fish | -0.070 | 0.009 | -7.864 | -0.087 | -0.052 | <0.001 |
| Gasterosteus | Fish | -0.027 | 0.057 | -0.463 | -0.139 | 0.086 | 0.644 |
| Hyperoplus | Fish | 0.047 | 0.022 | 2.201 | 0.005 | 0.090 | 0.028 |
| Lampetra | Fish | -0.013 | 0.025 | -0.505 | -0.062 | 0.037 | 0.614 |
| Limanda | Fish | -0.108 | 0.010 | -11.313 | -0.127 | -0.089 | <0.001 |
| Liparis | Fish | -0.019 | 0.019 | -1.019 | -0.056 | 0.018 | 0.308 |
| Merlangius | Fish | -0.026 | 0.010 | -2.639 | -0.045 | -0.007 | 0.008 |
| Microstomus | Fish | -0.285 | 0.048 | -5.937 | -0.378 | -0.191 | <0.001 |
| Myoxocephalus | Fish | -0.028 | 0.007 | -3.998 | -0.042 | -0.014 | <0.001 |
| Osmerus | Fish | -0.007 | 0.006 | -1.026 | -0.019 | 0.006 | 0.305 |
| Pholis | Fish | 0.037 | 0.013 | 2.869 | 0.012 | 0.063 | 0.004 |
| Platichthys | Fish | 0.022 | 0.007 | 3.282 | 0.009 | 0.034 | 0.001 |
| Pleuronectes | Fish | -0.034 | 0.006 | -5.753 | -0.046 | -0.023 | <0.001 |
| Pomatoschistus | Fish | -0.032 | 0.008 | -4.090 | -0.048 | -0.017 | <0.001 |
| Scophthalmus | Fish | 0.029 | 0.018 | 1.557 | -0.007 | 0.065 | 0.119 |
| Solea | Fish | -0.021 | 0.007 | -3.142 | -0.034 | -0.008 | 0.002 |
| Sprattus | Fish | -0.051 | 0.013 | -3.913 | -0.077 | -0.026 | <0.001 |
| Syngnathus | Fish | -0.032 | 0.008 | -3.976 | -0.048 | -0.016 | <0.001 |
| Trachurus | Fish | 0.083 | 0.020 | 4.162 | 0.044 | 0.122 | <0.001 |
| Trisopterus | Fish | -0.049 | 0.014 | -3.575 | -0.076 | -0.022 | <0.001 |
| Zoarces | Fish | -0.017 | 0.006 | -2.663 | -0.030 | -0.005 | 0.008 |
| Anas | Birds | 0.046 | 0.007 | 6.970 | 0.033 | 0.059 | <0.001 |
| Anser | Birds | 0.115 | 0.007 | 16.671 | 0.102 | 0.129 | <0.001 |
| Arenaria | Birds | 0.047 | 0.007 | 6.779 | 0.033 | 0.061 | <0.001 |
| Aythya | Birds | -0.059 | 0.090 | -0.651 | -0.235 | 0.118 | 0.515 |
| Branta | Birds | 0.060 | 0.007 | 9.105 | 0.047 | 0.073 | <0.001 |
| Calidris | Birds | 0.045 | 0.007 | 6.717 | 0.032 | 0.057 | <0.001 |
| Charadrius | Birds | 0.051 | 0.007 | 7.781 | 0.038 | 0.064 | <0.001 |
| Chlidonias | Birds | 0.268 | 0.037 | 7.197 | 0.195 | 0.341 | <0.001 |
| Cygnus | Birds | 0.064 | 0.017 | 3.869 | 0.032 | 0.097 | <0.001 |
| Gelochelidon | Birds | 0.018 | 0.009 | 2.131 | 0.001 | 0.035 | 0.033 |
| Haematopus | Birds | 0.032 | 0.007 | 4.894 | 0.019 | 0.045 | <0.001 |
| Larus | Birds | 0.042 | 0.006 | 6.557 | 0.030 | 0.055 | <0.001 |
| Limosa | Birds | 0.047 | 0.007 | 7.159 | 0.034 | 0.060 | <0.001 |
| Mareca | Birds | 0.041 | 0.007 | 6.021 | 0.028 | 0.055 | <0.001 |
| Melanitta | Birds | -0.008 | 0.049 | -0.172 | -0.104 | 0.087 | 0.863 |
| Numenius | Birds | 0.052 | 0.007 | 7.941 | 0.039 | 0.065 | <0.001 |
| Phalacrocorax | Birds | 0.110 | 0.008 | 13.314 | 0.094 | 0.126 | <0.001 |
| Philomachus | Birds | 0.016 | 0.009 | 1.664 | -0.003 | 0.034 | 0.096 |
| Platalea | Birds | 0.163 | 0.007 | 23.577 | 0.150 | 0.177 | <0.001 |
| Pluvialis | Birds | 0.053 | 0.007 | 8.037 | 0.040 | 0.066 | <0.001 |
| Recurvirostra | Birds | 0.034 | 0.007 | 5.100 | 0.021 | 0.047 | <0.001 |
| Somateria | Birds | 0.024 | 0.020 | 1.220 | -0.015 | 0.063 | 0.222 |
| Spatula | Birds | 0.074 | 0.007 | 10.608 | 0.061 | 0.088 | <0.001 |
| Sterna | Birds | 0.045 | 0.007 | 6.636 | 0.032 | 0.059 | <0.001 |
| Sternula | Birds | -0.002 | 0.007 | -0.309 | -0.016 | 0.011 | 0.757 |
| Tadorna | Birds | 0.048 | 0.007 | 7.303 | 0.035 | 0.060 | <0.001 |
| Tringa | Birds | 0.038 | 0.006 | 5.788 | 0.025 | 0.050 | <0.001 |
| Vanellus | Birds | 0.065 | 0.007 | 8.863 | 0.051 | 0.080 | <0.001 |
| Abra | MZB | 0.064 | 0.014 | 4.485 | 0.036 | 0.092 | <0.001 |
| Alitta | MZB | 0.158 | 0.017 | 9.309 | 0.125 | 0.191 | <0.001 |
| Ampharete | MZB | -0.104 | 0.039 | -2.656 | -0.180 | -0.027 | 0.008 |
| Aphelochaeta | MZB | 0.039 | 0.070 | 0.560 | -0.098 | 0.176 | 0.576 |
| Arenicola | MZB | -0.004 | 0.006 | -0.792 | -0.015 | 0.007 | 0.428 |
| Aricidea | MZB | 0.009 | 0.010 | 0.906 | -0.011 | 0.030 | 0.365 |
| Asterias | MZB | -0.071 | 0.079 | -0.897 | -0.225 | 0.084 | 0.370 |
| Balanus | MZB | 0.012 | 0.022 | 0.563 | -0.031 | 0.055 | 0.573 |
| Baltidrilus | MZB | -0.099 | 0.015 | -6.662 | -0.128 | -0.070 | <0.001 |
| Bathyporeia | MZB | -0.039 | 0.008 | -5.108 | -0.054 | -0.024 | <0.001 |
| Bodotria | MZB | 0.082 | 0.027 | 3.045 | 0.029 | 0.134 | 0.002 |
| Bylgides | MZB | -0.027 | 0.007 | -3.748 | -0.041 | -0.013 | <0.001 |
| Capitella | MZB | -0.013 | 0.012 | -1.100 | -0.036 | 0.010 | 0.272 |
| Carcinus | MZB | -0.008 | 0.006 | -1.490 | -0.019 | 0.003 | 0.136 |
| Centropages | Zooplankton | -0.301 | 0.086 | -3.513 | -0.469 | -0.133 | <0.001 |
| Cerastoderma | MZB | -0.009 | 0.006 | -1.312 | -0.021 | 0.004 | 0.190 |
| Corophium | MZB | -0.005 | 0.009 | -0.521 | -0.022 | 0.013 | 0.603 |
| Crangon | MZB | -0.003 | 0.005 | -0.540 | -0.014 | 0.008 | 0.589 |
| Crepidula | MZB | 0.037 | 0.094 | 0.391 | -0.147 | 0.221 | 0.696 |
| Donax | MZB | 0.066 | 0.142 | 0.463 | -0.212 | 0.343 | 0.643 |
| Echinocarium | MZB | -0.156 | 0.097 | -1.613 | -0.345 | 0.033 | 0.107 |
| Ensis | MZB | 0.071 | 0.010 | 7.208 | 0.052 | 0.091 | <0.001 |
| Eteone | MZB | 0.030 | 0.006 | 5.209 | 0.019 | 0.041 | <0.001 |
| Eunereis | MZB | -0.017 | 0.097 | -0.180 | -0.207 | 0.172 | 0.857 |
| Euterpina | Zooplankton | 0.178 | 0.094 | 1.906 | -0.005 | 0.362 | 0.057 |
| Fabulina | MZB | 0.101 | 0.066 | 1.534 | -0.028 | 0.230 | 0.125 |
| Gammarus | MZB | 0.022 | 0.012 | 1.945 | <0.001 | 0.045 | 0.052 |
| Gastrosaccus | MZB | -0.073 | 0.070 | -1.053 | -0.210 | 0.063 | 0.292 |
| Gattyana | MZB | 0.057 | 0.079 | 0.719 | -0.098 | 0.212 | 0.472 |
| Hediste | MZB | 0.013 | 0.006 | 2.217 | 0.001 | 0.024 | 0.027 |
| Hemigrapsus | MZB | 0.297 | 0.047 | 6.397 | 0.206 | 0.389 | <0.001 |
| Heteromastus | MZB | 0.007 | 0.006 | 1.181 | -0.005 | 0.018 | 0.238 |
| Jaera | MZB | -0.080 | 0.065 | -1.232 | -0.208 | 0.047 | 0.218 |
| Kurtiella | MZB | 0.019 | 0.052 | 0.359 | -0.084 | 0.121 | 0.719 |
| Lagis | MZB | 0.132 | 0.044 | 3.006 | 0.046 | 0.218 | 0.003 |
| Lanice | MZB. Zooplankton | 0.014 | 0.007 | 1.847 | -0.001 | 0.028 | 0.065 |
| Lepidochitona | MZB | -0.072 | 0.033 | -2.168 | -0.137 | -0.007 | 0.030 |
| Limnodrilus | MZB | -0.391 | 0.044 | -8.895 | -0.478 | -0.305 | <0.001 |
| Liocarcinus | MZB | 0.003 | 0.010 | 0.254 | -0.017 | 0.023 | 0.799 |
| Macoma | MZB | -0.047 | 0.006 | -8.404 | -0.058 | -0.036 | <0.001 |
| Macomangulus | MZB | -0.021 | 0.012 | -1.833 | -0.044 | 0.001 | 0.067 |
| Magallana | MZB | 0.268 | 0.056 | 4.748 | 0.157 | 0.378 | <0.001 |
| Magelona | MZB. Zooplankton | -0.042 | 0.009 | -4.724 | -0.060 | -0.025 | <0.001 |
| Malmgrenia | MZB | 0.088 | 0.074 | 1.195 | -0.057 | 0.233 | 0.232 |
| Manayunkia | MZB | -0.025 | 0.097 | -0.257 | -0.215 | 0.165 | 0.797 |
| Marenzelleria | MZB | 0.120 | 0.011 | 11.276 | 0.100 | 0.141 | <0.001 |
| Mesodinium | Zooplankton | -0.131 | 0.043 | -3.024 | -0.216 | -0.046 | 0.002 |
| Mesopodopsis | Zooplankton | -0.014 | 0.049 | -0.291 | -0.110 | 0.081 | 0.771 |
| Mya | MZB | -0.007 | 0.006 | -1.279 | -0.019 | 0.004 | 0.201 |
| Myrianida | MZB | 0.139 | 0.070 | 2.002 | 0.003 | 0.276 | 0.045 |
| Mytilus | MZB | -0.004 | 0.010 | -0.376 | -0.024 | 0.016 | 0.707 |
| Neomysis | MZB | 0.007 | 0.049 | 0.146 | -0.089 | 0.104 | 0.884 |
| Nephtys | MZB | -0.048 | 0.006 | -8.074 | -0.060 | -0.037 | <0.001 |
| Oikopleura | Zooplankton | -0.018 | 0.057 | -0.310 | -0.129 | 0.094 | 0.757 |
| Oithona | Zooplankton | 0.367 | 0.084 | 4.351 | 0.201 | 0.532 | <0.001 |
| Ophiura | MZB | 0.037 | 0.017 | 2.177 | 0.004 | 0.071 | 0.029 |
| Paracalanus | Zooplankton | -0.144 | 0.068 | -2.127 | -0.277 | -0.011 | 0.033 |
| Paranais | MZB | -0.178 | 0.038 | -4.659 | -0.252 | -0.103 | <0.001 |
| Peringia | MZB | -0.007 | 0.010 | -0.649 | -0.027 | 0.013 | 0.517 |
| Phoronis | MZB | -0.041 | 0.205 | -0.199 | -0.442 | 0.361 | 0.843 |
| Phyllodoce | MZB | -0.019 | 0.006 | -2.925 | -0.031 | -0.006 | 0.003 |
| Polydora | MZB. Zooplankton | 0.009 | 0.021 | 0.452 | -0.032 | 0.051 | 0.651 |
| Pontocrates | MZB | 0.013 | 0.048 | 0.280 | -0.080 | 0.107 | 0.780 |
| Pseudocalanus | Zooplankton | -0.106 | 0.140 | -0.755 | -0.380 | 0.169 | 0.450 |
| Pseudodiaptomus | Zooplankton | 0.044 | 0.065 | 0.674 | -0.083 | 0.170 | 0.500 |
| Pygospio | MZB | 0.011 | 0.008 | 1.393 | -0.004 | 0.026 | 0.164 |
| Retusa | MZB | -0.097 | 0.019 | -5.093 | -0.135 | -0.060 | <0.001 |
| Schistomysis | MZB | -0.099 | 0.028 | -3.481 | -0.155 | -0.043 | <0.001 |
| Scolelepis | MZB | -0.038 | 0.006 | -5.958 | -0.051 | -0.026 | <0.001 |
| Scoloplos | MZB | 0.007 | 0.006 | 1.170 | -0.005 | 0.019 | 0.242 |
| Scrobicularia | MZB | 0.006 | 0.006 | 0.961 | -0.006 | 0.018 | 0.337 |
| Spio | MZB | 0.016 | 0.012 | 1.336 | -0.007 | 0.039 | 0.181 |
| Spiophanes | MZB | -0.018 | 0.009 | -1.956 | -0.036 | <0.001 | 0.050 |
| Streblospio | MZB | -0.093 | 0.027 | -3.397 | -0.147 | -0.039 | 0.001 |
| Tellimya | MZB | 0.040 | 0.110 | 0.367 | -0.175 | 0.256 | 0.714 |
| Temora | Zooplankton | -0.104 | 0.043 | -2.414 | -0.188 | -0.020 | 0.016 |
| Tharyx | MZB | 0.069 | 0.011 | 6.072 | 0.047 | 0.091 | <0.001 |
| Tubificoides | MZB | 0.058 | 0.008 | 7.641 | 0.043 | 0.073 | <0.001 |
| Urothoe | MZB | -0.001 | 0.012 | -0.060 | -0.024 | 0.023 | 0.952 |

#### **Table S9**.  List of PANGAEA data sources for stownet fish data counts in the Eastfrisian Wadden Sea.

| Year | DOI |
| --- | --- |
| 1993 | https://doi.pangaea.de/10.1594/PANGAEA.762284 |
| 1993 | https://doi.pangaea.de/10.1594/PANGAEA.762094 |
| 1993 | https://doi.pangaea.de/10.1594/PANGAEA.762297 |
| 1993 | https://doi.pangaea.de/10.1594/PANGAEA.762302 |
| 1993 | https://doi.pangaea.de/10.1594/PANGAEA.762292 |
| 1993 | https://doi.pangaea.de/10.1594/PANGAEA.762275 |
| 1993 | https://doi.pangaea.de/10.1594/PANGAEA.762280 |
| 1993 | https://doi.pangaea.de/10.1594/PANGAEA.762217 |
| 1993 | https://doi.pangaea.de/10.1594/PANGAEA.762290 |
| 1993 | https://doi.pangaea.de/10.1594/PANGAEA.762294 |
| 1993 | https://doi.pangaea.de/10.1594/PANGAEA.762287 |
| 1993 | https://doi.pangaea.de/10.1594/PANGAEA.762300 |
| 1993 | https://doi.pangaea.de/10.1594/PANGAEA.762237 |
| 1994 | https://doi.pangaea.de/10.1594/PANGAEA.762281 |
| 1994 | https://doi.pangaea.de/10.1594/PANGAEA.762285 |
| 1994 | https://doi.pangaea.de/10.1594/PANGAEA.762276 |
| 1994 | https://doi.pangaea.de/10.1594/PANGAEA.762218 |
| 1994 | https://doi.pangaea.de/10.1594/PANGAEA.762288 |
| 1994 | https://doi.pangaea.de/10.1594/PANGAEA.762291 |
| 1994 | https://doi.pangaea.de/10.1594/PANGAEA.762293 |
| 1994 | https://doi.pangaea.de/10.1594/PANGAEA.762296 |
| 1995 | https://doi.pangaea.de/10.1594/PANGAEA.762239 |
| 1995 | https://doi.pangaea.de/10.1594/PANGAEA.762219 |
| 1995 | https://doi.pangaea.de/10.1594/PANGAEA.762289 |
| 1995 | https://doi.pangaea.de/10.1594/PANGAEA.762282 |
| 1995 | https://doi.pangaea.de/10.1594/PANGAEA.762277 |
| 1995 | https://doi.pangaea.de/10.1594/PANGAEA.762286 |
| 1996 | https://doi.pangaea.de/10.1594/PANGAEA.762278 |
| 1996 | https://doi.pangaea.de/10.1594/PANGAEA.762240 |
| 1996 | https://doi.pangaea.de/10.1594/PANGAEA.762220 |
| 1996 | https://doi.pangaea.de/10.1594/PANGAEA.762283 |
| 1997 | https://doi.pangaea.de/10.1594/PANGAEA.762241 |
| 1997 | https://doi.pangaea.de/10.1594/PANGAEA.762221 |
| 1998 | https://doi.pangaea.de/10.1594/PANGAEA.762242 |
| 1998 | https://doi.pangaea.de/10.1594/PANGAEA.762279 |
| 1998 | https://doi.pangaea.de/10.1594/PANGAEA.762222 |
| 1999 | https://doi.pangaea.de/10.1594/PANGAEA.762243 |
| 1999 | https://doi.pangaea.de/10.1594/PANGAEA.762223 |
| 2000 | https://doi.pangaea.de/10.1594/PANGAEA.762206 |
| 2001 | https://doi.pangaea.de/10.1594/PANGAEA.762207 |
| 2001 | https://doi.pangaea.de/10.1594/PANGAEA.762225 |
| 2002 | https://doi.pangaea.de/10.1594/PANGAEA.762226 |
| 2002 | https://doi.pangaea.de/10.1594/PANGAEA.762208 |
| 2003 | https://doi.pangaea.de/10.1594/PANGAEA.762246 |
| 2003 | https://doi.pangaea.de/10.1594/PANGAEA.762209 |
| 2003 | https://doi.pangaea.de/10.1594/PANGAEA.762227 |
| 2004 | https://doi.pangaea.de/10.1594/PANGAEA.762212 |
| 2004 | https://doi.pangaea.de/10.1594/PANGAEA.762228 |
| 2005 | https://doi.pangaea.de/10.1594/PANGAEA.762229 |
| 2005 | https://doi.pangaea.de/10.1594/PANGAEA.762213 |
| 2006 | https://doi.pangaea.de/10.1594/PANGAEA.762214 |
| 2006 | https://doi.pangaea.de/10.1594/PANGAEA.762230 |
| 2007 | https://doi.pangaea.de/10.1594/PANGAEA.762215 |
| 2007 | https://doi.pangaea.de/10.1594/PANGAEA.762235 |
